# Evolutionary Conservation and Diversification of Puf RNA Binding Proteins and Their mRNA Targets

**DOI:** 10.1371/journal.pbio.1002307

**Published:** 2015-11-20

**Authors:** Gregory J. Hogan, Patrick O. Brown, Daniel Herschlag

**Affiliations:** 1 Department of Biochemistry, Stanford University School of Medicine, Stanford, California, United States of America; 2 Howard Hughes Medical Institute, Stanford University School of Medicine, Stanford, California, United States of America; 3 Department of Chemistry, Stanford University, Stanford, California, United States of America; 4 Department of Chemical Engineering, Stanford University, Stanford, California, United States of America; 5 ChEM-H Institute, Stanford University, Stanford, California, United States of America; Case Western Reserve University, UNITED STATES

## Abstract

Reprogramming of a gene’s expression pattern by acquisition and loss of sequences recognized by specific regulatory RNA binding proteins may be a major mechanism in the evolution of biological regulatory programs. We identified that RNA targets of Puf3 orthologs have been conserved over 100–500 million years of evolution in five eukaryotic lineages. Focusing on Puf proteins and their targets across 80 fungi, we constructed a parsimonious model for their evolutionary history. This model entails extensive and coordinated changes in the Puf targets as well as changes in the number of Puf genes and alterations of RNA binding specificity including that: 1) Binding of Puf3 to more than 200 RNAs whose protein products are predominantly involved in the production and organization of mitochondrial complexes predates the origin of budding yeasts and filamentous fungi and was maintained for 500 million years, throughout the evolution of budding yeast. 2) In filamentous fungi, remarkably, more than 150 of the ancestral Puf3 targets were gained by Puf4, with one lineage maintaining both Puf3 and Puf4 as regulators and a sister lineage losing Puf3 as a regulator of these RNAs. The decrease in gene expression of these mRNAs upon deletion of Puf4 in filamentous fungi (*N*. *crassa*) in contrast to the increase upon Puf3 deletion in budding yeast (*S*. *cerevisiae*) suggests that the output of the RNA regulatory network is different with Puf4 in filamentous fungi than with Puf3 in budding yeast. 3) The coregulated Puf4 target set in filamentous fungi expanded to include mitochondrial genes involved in the tricarboxylic acid (TCA) cycle and other nuclear-encoded RNAs with mitochondrial function not bound by Puf3 in budding yeast, observations that provide additional evidence for substantial rewiring of post-transcriptional regulation. 4) Puf3 also expanded and diversified its targets in filamentous fungi, gaining interactions with the mRNAs encoding the mitochondrial electron transport chain (ETC) complex I as well as hundreds of other mRNAs with nonmitochondrial functions. The many concerted and conserved changes in the RNA targets of Puf proteins strongly support an extensive role of RNA binding proteins in coordinating gene expression, as originally proposed by Keene. Rewiring of Puf-coordinated mRNA targets and transcriptional control of the same genes occurred at different points in evolution, suggesting that there have been distinct adaptations via RNA binding proteins and transcription factors. The changes in Puf targets and in the Puf proteins indicate an integral involvement of RNA binding proteins and their RNA targets in the adaptation, reprogramming, and function of gene expression.

## Abstract 2

A map of the evolutionary history of Puf proteins and their RNA targets shows that reprogramming of global gene expression programs via adaptive mutations that affect protein-RNA interactions is an important source of biological diversity.

## Author Summary

We set out to trace the evolutionary history of an RNA binding protein and how its interactions with targets change over evolution. Identifying this natural history is a step toward understanding the critical differences between organisms and how gene expression programs are rewired during evolution. Using bioinformatics and experimental approaches, we broadly surveyed the evolution of binding targets of a particular family of RNA binding proteins—the Puf proteins, whose protein sequences and target RNA sequences are relatively well-characterized—across 99 eukaryotic species. We found five groups of species in which targets have been conserved for at least 100 million years and then took advantage of genome sequences from a large number of fungal species to deeply investigate the conservation and changes in Puf proteins and their RNA targets. Our analyses identified multiple and extensive reconfigurations during the natural history of fungi and suggest that RNA binding proteins and their RNA targets are profoundly involved in evolutionary reprogramming of gene expression and help define distinct programs unique to each organism. Continuing to uncover the natural history of RNA binding proteins and their interactions will provide a unique window into the gene expression programs of present day species and point to new ways to engineer gene expression programs.

## Introduction

The phenotypic diversity of life on earth results not only from differences in the proteins encoded by each genome but, perhaps even more, from differences in the programs that specify where, when, under what conditions, and at what levels these proteins are expressed. A grand challenge in biology is to understand these gene expression programs. Uncovering the similarities in and differences between gene expression programs in related organisms can help reveal fundamental properties of these programs, how they have evolved, how they may be wired and rewired, and ultimately how they can be engineered.

The seminal step in gene expression and the focus of much current effort is the initiation of transcription through transcription factors that bind in proximity to genes and regulate the timing and magnitude of RNA synthesis (see [1–7] for reviews). Each transcription factor regulates a set of genes, numbering a few to thousands, specified by short DNA sequences that are in proximity to those genes and are recognized by that transcription factor. One major mechanism for diversification of gene expression programs is the loss or gain of regulation by individual transcription factors, due to mutations that, respectively, disrupt or create the proximal recognition sequences (see [8–13] for reviews). The binding specificity, regulation, and targets of a transcription factor tend to be conserved over a short evolutionary timescale, but each of these properties has changed over evolution, allowing the regulatory roles of orthologous transcription factors to diverge and diversify.

Evolutionary changes in regulation at the next level of gene expression are virtually unexplored. After transcription, each messenger RNA (mRNA) undergoes a functional odyssey and can be regulated at steps that include splicing, transport, localization, translation, and decay [14]. RNA binding proteins function in each step, and each mRNA interacts with many RNA binding proteins over its lifetime [15–22]. Each RNA binding protein can recognize a few to thousands of mRNAs, and the target sets of each individual RNA binding protein often share functional themes, encoding proteins involved in a particular biological process or localized to the same part of the cell [15,23–37]. These effects can be described in terms of a model originally referred to as the “RNA operon” model in which RNA binding proteins bind to and coordinate the regulation of mRNAs encoding functionally or cytotopically related proteins [18,20,21].

We set out to trace the evolutionary history of an RNA binding protein and how its interactions with targets change over evolution. Identifying this natural history is a step toward understanding the critical differences between organisms, how evolution has progressed, why these differences have arisen, and how gene expression programs are “wired.”

**Fig 1:**
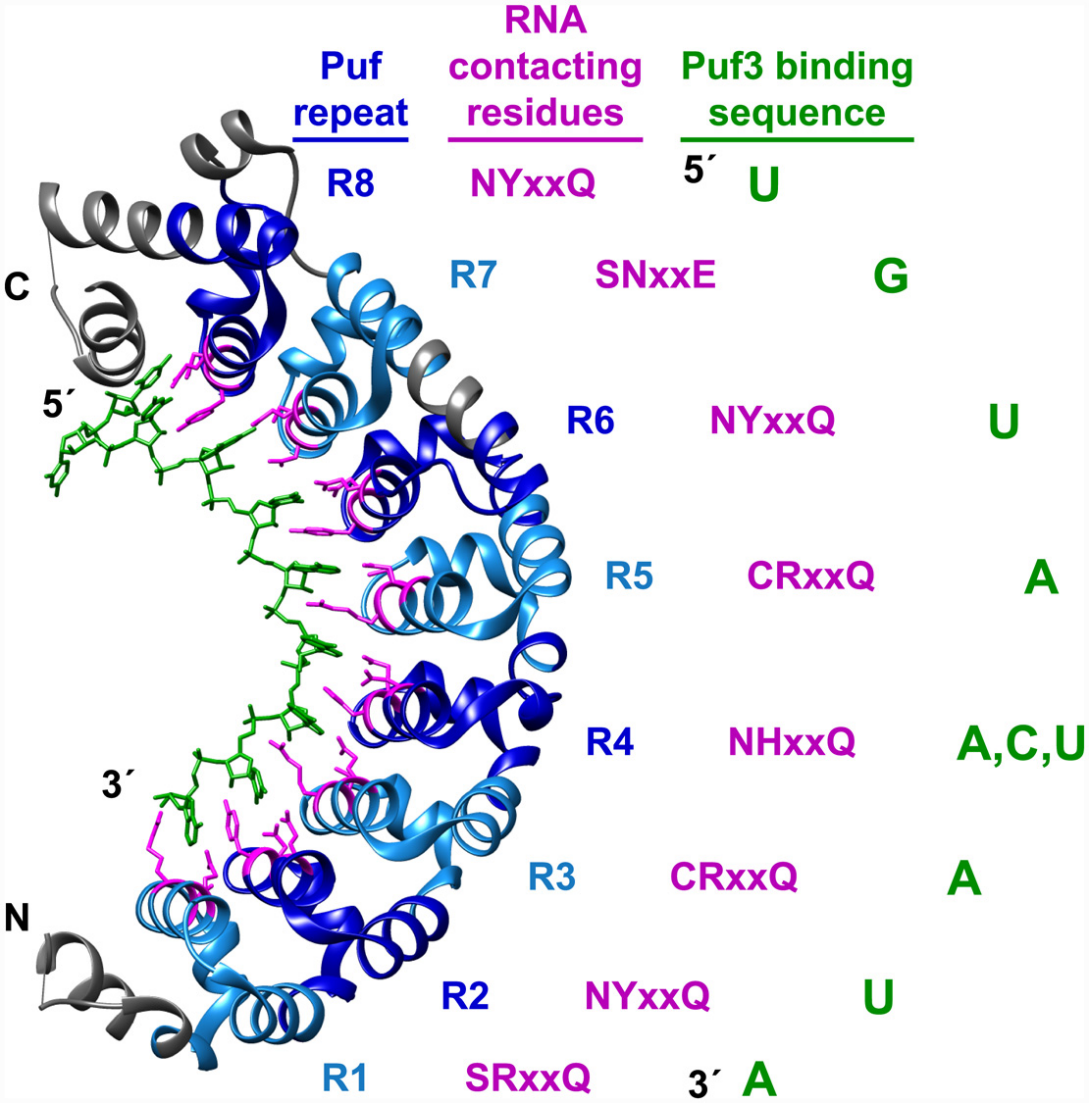
Puf3 recognition of RNA. *S*. *cerevisiae* Puf3 structure displayed as a ribbon model from PDB (Protein Data Bank) entry 3k49 [49]. The RNA molecule with sequence CCUGUAAAUA is indicated in green. Puf repeats are highlighted in alternating light and dark blue. The Puf proteins with the same RNA-base-contacting amino acids as Puf3 (pink) that have been studied have a conserved RNA binding specificity [24,25,27,29,50,51]; the Puf proteins with different RNA-base-contacting amino acids have different RNA binding specificities [25,31,51–55]. The conserved RNA binding sequence is eight nucleotides, containing UGUA at the 5' end, flexibility for A, C, or U at the 5th position, and AUA at the 3' end. Mutating the RNA-interacting amino acids alters RNA binding specificity and can be done to make predictable changes in sequence specificity [51,56–59]. *S*. *cerevisiae* Puf3 contains additional residues that contact a nucleotide upstream of the core binding sequence, but these interactions are not conserved across eukaryotes.

We chose to investigate the Puf (Pumilio–Fem-3-binding factor) family of RNA binding proteins, taking particular advantage of the relatively well-understood relationship between Puf protein sequences and the specific RNA sequences they recognize (Fig 1). Puf proteins are found in most, if not all, eukaryotes [38–40] and have been implicated in regulating the decay, translation, and localization of distinct sets of functionally related RNA targets [38,41–43]. For example, in *Saccharomyces cerevisiae* Puf3 binds and regulates hundreds of distinct RNAs transcribed from the nuclear genome that, almost without exception, encode for proteins localized to the mitochondrion [25]. Puf3 promotes localization of its target mRNAs to the periphery of mitochondria [44–46] and can repress the expression of these mRNAs by promoting their decay [25,47,48]. Puf3 recognizes a specific sequence element usually found in the 3' untranslated region (3' UTR) of its targets (Fig 1) [25].

*S*. *cerevisiae* Puf3 and its orthologs in *Drosophila melanogaster* (Pumilio) and *Homo sapiens* (Pum1 and Pum2) recognize nearly identical RNA sequence motifs, but they bind to distinct sets of mRNAs that encode proteins with distinct functional themes [24,25,27,29]. Fewer than 20% of the targets of the Puf3 orthologs in humans and flies are themselves orthologs [24,29], and the functional themes of their mRNA targets in flies and humans starkly contrast with those for yeast Puf3 [24,27,29]. Thus, the mRNA targets of Puf3 orthologs have diverged since humans, flies, and yeast shared ancestors. Nevertheless, bioinformatics studies have suggested that Puf targets are conserved over short timescales, underscoring the importance of these distinct interactions [60–65].

We first systematically investigated the conservation and divergence of the RNA targets that are likely to be recognized by orthologs of *S*. *cerevisiae* Puf3 in diverse eukaryotes. We then focused in detail on the larger family of Puf RNA binding proteins and their RNA targets in fungi, as the many sequenced fungal genomes provide the power to identify major and minor evolutionary changes in the repertoires of Puf proteins, their binding specificities, and their RNA targets. The numerous and often concerted changes in this single family of proteins and their RNA targets provide strong corroborative evidence for the role of coordinated protein binding to sets of related mRNAs in organizing gene expression [18,20,21]. The observed extensive evolutionary changes suggest that changes in RNA binding proteins and their interacting mRNAs are an important source of biological diversification and specialization; studies of these changes across evolutionary time may provide a powerful complement to traditional deep investigations of specific model organisms.

## Results and Discussion

### Evolutionary Interplay between Puf3 and Its RNA Targets

We searched for orthologs of *S*. *cerevisiae* Puf3 in 99 diverse eukaryotes (S1 Text, S1 Fig, S1 Table, Materials and Methods) and used the identified orthologs to determine the conservation of features important for RNA binding specificity. Puf3 is a canonical Puf protein containing eight Puf repeats [39,40,66,67] that together fold to form a characteristic crescent shape with an RNA binding interface on the inner side (Fig 1) [54,55,59,68–73]. Three amino acid residues within each Puf repeat typically contact an RNA base directly and are important determinants of RNA binding specificity (Fig 1 legend and references [49,54,55,59,68–73]).

The observations that Puf3 orthologs have a distinctly conserved pocket around the bound RNA and that the residues that determine RNA binding specificity are especially conserved suggest that orthologs of Puf3 recognize the same RNA sequence motifs (S2 Text, S2 Fig). This inference is consistent with experimental results from Puf3 orthologs in diverse eukaryotes [24,25,27,29,50,51]. We used this insight to infer, by analysis of RNA sequences, the extent to which the RNA targets of Puf3 are conserved.

#### RNA target sets of Puf3 orthologs are distinct and conserved in five eukaryotic lineages

We investigated the conservation and divergence in the sets of orthologous RNA recognized by Puf3 orthologs in diverse eukaryotes by evaluating the frequency with which orthologous RNAs contained a 3' UTR sequence that is recognized by the Puf3 protein family (i.e., UGUA[ACU]AUA). When a larger than expected fraction of the orthologous transcripts contained Puf3 recognition elements, relative to a null model (see Materials and Methods), we inferred that those targets were conserved from a common ancestor.

To measure the conservation of targets between each pair of 99 eukaryote species, we applied a network-level approach similar to that implemented by the program Fastcompare [63,74,75]. We first determined orthologous sequence sets for each pair of species and then determined the number of ortholog pairs that both contained a putative Puf3 binding site. This number was then compared to the number expected by chance, given the frequency of sequences with putative Puf3 binding sites in each species (using the hypergeometric test, Materials and Methods). To control for the extent of sequence similarity expected for each set of two species, the result with the Puf3 motif was compared to results from all permutations of this motif under a model that the permutated motifs are neutral with respect to natural selection (S3 Fig).

**Fig 2:**
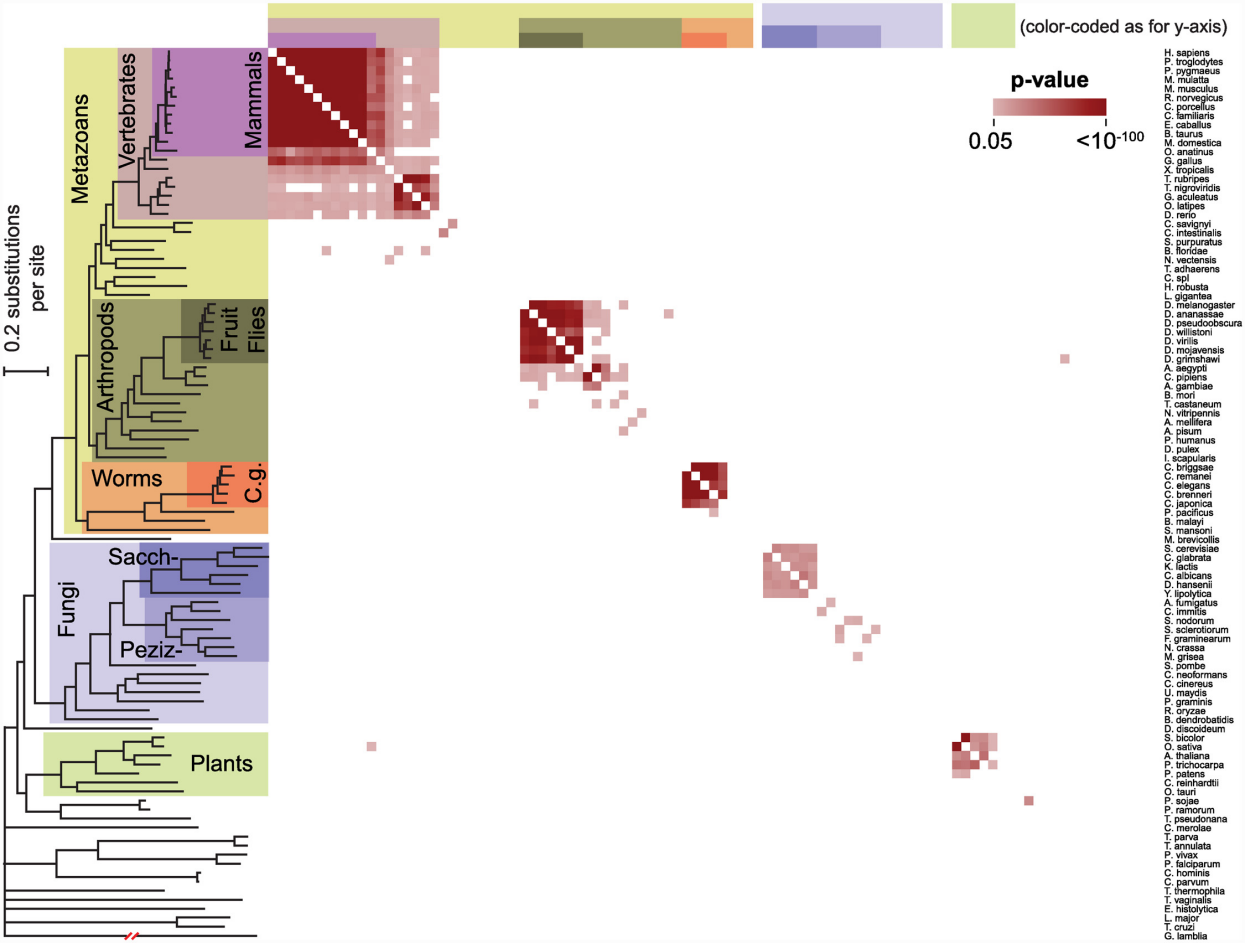
Conservation of Puf3 RNA targets across eukaryotes. A heatmap displaying whether the Puf3 binding sequence UGUA[ACU]AUA is shared by orthologs in a pair of species. A significant *p*-value is displayed as a colored square and was determined by the hypergeometric test. Color is used only if the Puf3 motif ranks in the top 1% relative to all motif permutations (Materials and Methods). (The heatmap is not fully symmetrical since the orthology assignments are not necessarily reciprocal.) The phylogeny shown was inferred using a maximum-likelihood approach from a concatenated alignment of 53 protein sequences (Materials and Methods). "Sacch-" refers to Saccharomycotina fungi, "Peziz-" to Pezizomycotina fungi, and "C.g." to the *Caenorhabditis* genus of worms. The break noted in red corresponds to 0.5 substitutions per site in the branch to *Giardia lamblia*. *p*-Values and ranks of the Puf3 motif against its permutations can be found in S9 Dataset.

We found evidence for conservation of Puf3 targets within each of five taxonomic groups (Fig 2): (1) vertebrates; (2) fruit flies and mosquitoes; (3) *Caenorhabditis* worms; (4) budding yeasts of Saccharomycotina; (5) and land plants. The most recent common ancestor of each of these five groups lived approximately 500, 250, 100, 300, and 470 million years ago, respectively [76–79].

Despite evidence for conservation within the five groups noted above, we found no evidence for conservation of the Puf3 regulatory program or a subset of that program between any pair of the five groups (Fig 2). No pairwise comparisons between the groups were statistically significant.

The common ancestor of all of these groups presumably had a Puf3 protein and a single set of RNA targets. These findings suggest divergence of the Puf3-mediated regulatory programs prior to establishment of the five lineages, despite the strong conservation of Puf3’s RNA-sequence specificity. The subsequent conservation of distinct targets within lineages strongly suggests significant selective pressure operating to maintain Puf3’s interactions and thus its regulatory roles and provides additional evidence for distinct roles of Puf3 orthologs in different organisms and lineages. In addition, our estimates of the timing of major changes in Puf3's RNA targets can be compared to the inferred timing of changes in other aspects of the gene expression programs to better understand the changes in gene regulation through evolution and the interplay of gene regulatory elements in the gene expression programs unique to each species.

### Puf Proteins and Their RNA Targets in Fungi

The diversity of the fungal kingdom is a result of more than one billion years of evolution [77], and the many available sequenced genomes and their relatively low complexity render fungi accessible and powerful for evolutionary studies. Here we synthesize the sequence data with biochemical and functional data to build a model of the evolution of Puf proteins and their targets in fungi.

#### Evolutionary reprogramming of post-transcriptional regulation: concerted changes in Puf3 targets

Puf3 in fungi provides a starting point for dissecting how target sets of an RNA binding protein diversify over evolution. As noted above, the *S*. *cerevisiae* Puf3 protein binds to more than 200 mRNAs [25], nearly all of which encode proteins that function in the mitochondrion and in particular act in mitochondrial organization, biogenesis, and translation [25,44]. We and others have noted a general conservation of these Puf3 targets in Saccharomycotina (Fig 2) [62,64]. The analysis in the preceding section suggested that the predicted Puf3 targets in the sister Pezizomycotina lineage do not share a detectable similarity with the Saccharomycotina Puf3 targets for the species studied (Fig 2), as did a previous less extensive analysis [60]. A more detailed analysis (below) leads us to a model for the nature and timing of these and other evolutionary changes.

#### Puf3 orthologs in Saccharomycotina and early-diverging Pezizomycotina species bind a common set of RNAs

**Fig 3:**
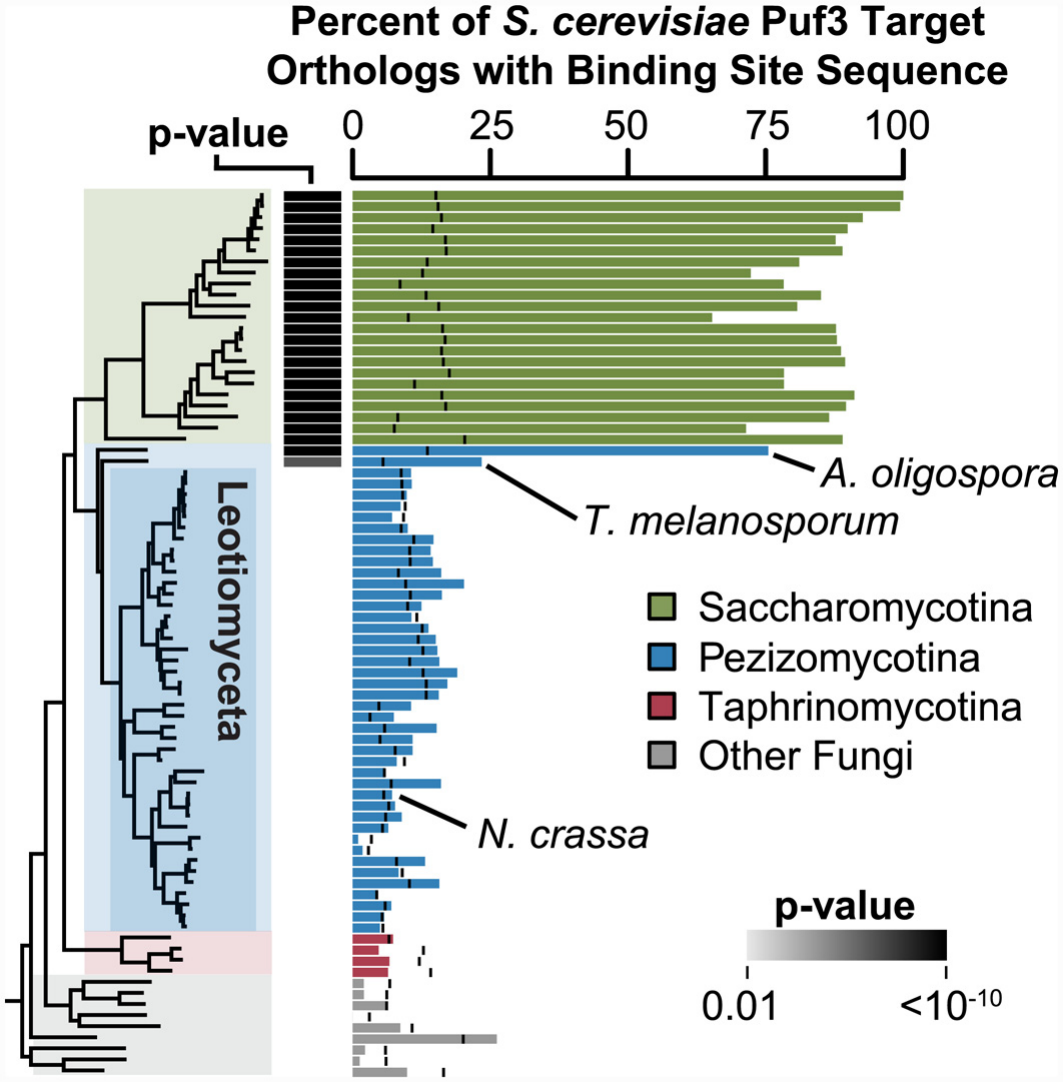
Conservation of *S*. *cerevisiae* Puf3 targets in other fungi. The percent of orthologs of *S*. *cerevisiae* Puf3 targets (identified by Gerber et al. [25]) that have a match to the Puf3 motif UGUA[ACU]AUA in the 3' UTRs in each species is shown as a bar plot. A black vertical bar marks the background frequency of the Puf3 motif for each species. The 3' UTR was defined as the 500 nucleotides downstream of the stop codon. Only orthologs of *S*. *cerevisiae* Puf3 targets that have a Puf3 motif match in 3' UTR were used (*n* = 176) so that *S*. *cerevisiae* S288C is set at 100%. A significant *p*-value (gray or black box) indicates that Puf3 motif matches are enriched in orthologs of *S*. *cerevisiae* Puf3 targets relative to all other orthologs. *p*-Values were computed using Fisher's exact test and are Bonferroni corrected for multiple hypothesis testing by multiplication by 80, the number of species tested. The phylogeny represents the evolutionary relationships of the fungi, inferred with a maximum-likelihood approach from a concatenated alignment of 20 protein sequences (Materials and Methods). Species names can be found in S19 Fig Percentages and *p*-values can be found in S1 Dataset.

We sought to identify which fungi have a Puf3 protein that binds mRNA targets orthologous to the mRNA targets of *S*. *cerevisiae* Puf3, using sequence data from 80 fungi, including 23 from Saccharomycotina, 44 from Pezizomycotina, and 13 from other fungi (Materials and Methods). Of the 210 *S*. *cerevisiae* Puf3 mRNA targets identified experimentally [25], 176 (84%) contain a match to the Puf3 motif in the 500 nucleotides downstream of the stop codon, which presumably includes all or nearly all of the 3’ UTR [80]. We tracked conservation of Puf3 binding to RNAs orthologous to these 176 *S*. *cerevisiae* Puf3 targets, using the Puf3 motif as the insignia of Puf3 binding targets and the same operational definition of 3’ UTRs (Fig 3). Matches to the Puf3 motif are also found, albeit rarely, in the 3' UTRs of mRNAs not experimentally identified as Puf3 targets. To control for the background frequency of the presumptive Puf3 binding site in nontarget RNAs, we tested whether the enrichment of Puf3 motif matches in orthologs of Puf3 targets exceeded their overall frequency in all 3' UTRs for that species (Fig 3). In all species in the Saccharomycotina subphylum, matches to the motif recognized by Puf3 are enriched in orthologs of *S*. *cerevisiae* Puf3 targets (*p* < 10^−50^, Fig 3), consistent with our results above and with previous results that traced the conservation of Puf3 targets to the ancestor of Saccharomycotina [62,64].

These comparisons also identified two species from the neighboring Pezizomycotina subphylum in which the Puf3 recognition element was significantly enriched in the orthologs of *S*. *cerevisiae* Puf3 targets (Fig 3, *p* = 10^−42^ and 10^−7^). *Arthrobotrys oligospora* and *Tuber melanosporum* were the earliest to diverge from the remainder of the Pezizomycotina species analyzed herein (hereafter Leotiomyceta; see S19 Fig for phylogeny with species names).

The phylogenetic relationships of these fungi suggest a parsimonious evolutionary model in which the regulatory program embodied by Puf3 and its RNA targets in *S*. *cerevisiae* has been conserved since the Saccharomycotina and Pezizomycotina fungi diverged from their common ancestor, which is estimated to have occurred 500 million years ago [77,78,81]. Our results provide strong evidence that the regulation of mitochondrial protein transcripts by Puf3 is not unique to Saccharomycotina, in contrast to the conclusion from previous work [62]. As described below (see “Evolutionary Transition of the Regulation of a Large Set of Mitochondria-Related Genes from Puf3 to Puf4"), analysis of additional early-diverging Pezizomycotina species provided further evidence for this timing and additional insight into this apparent regulatory reprogramming. An alternative parsimonious model is discussed in S14 Text.

#### Leotiomyceta Puf3 shares a conserved binding specificity with *S*. *cerevisiae* Puf3 but interacts with a functionally distinct set of RNAs

The RNAs with putative Puf3 binding sites in the remaining 42 Leotiomyceta species (i.e., the Pezizomycotina species other than *A*. *oligospora* and *T*. *melanosporum*) have little in common with the Puf3 targets in *S*. *cerevisiae* (Fig 3). Two models could explain this divergence: Leotiomyceta Puf3 proteins changed their RNA sequence specificity and maintain the same targets as *S*. *cerevisiae* Puf3, or the Leotiomyceta Puf3 proteins retained their RNA sequence specificity but the sequences recognized by Puf3 were lost in the original RNA targets and acquired by a distinct new set of RNAs.

**Fig 4:**
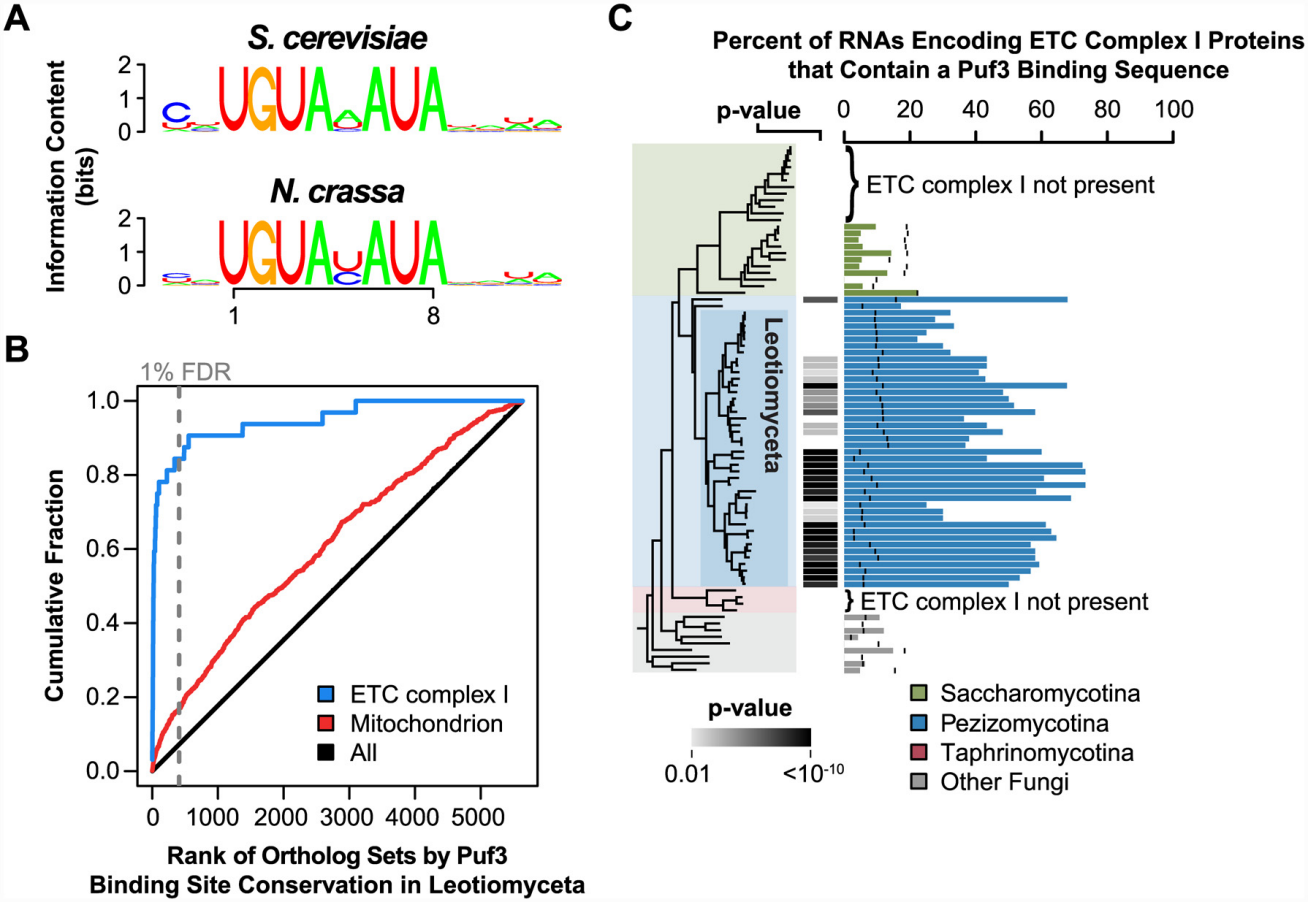
Binding specificity and targets of Leotiomyceta Puf3. (A) Motifs identified by the motif discovery program FIRE [82] within the 3' UTRs of *S*. *cerevisiae* mRNAs associated with *S*. *cerevisiae* Puf3 or the ectopically expressed *N*. *crassa* Puf3. FIRE identified eight nucleotide motifs, and these motifs were extended to display any preferences for flanking nucleotides. A match to the Puf3 motif UGUA[ACU]AUA is found in the 3' UTRs of 241 out of 392 (61%) of *S*. *cerevisiae* Puf3 targets and 83 out of 250 (33%) of mRNAs associated with *N*. *crassa* Puf3. The motif frequency across all 3' UTRs is 11%. Position frequency matrices can be found in S5 Dataset. (B) Cumulative distributions based on the ranked conservation score for Puf3 sites across Leotiomyceta species. The gray dashed line represents the 1% false discovery rate (FDR) cutoff for ortholog sets within which Puf3 sites are considered conserved. *N*. *crassa* annotations were used to define mitochondrion and electron transport chain (ETC) complex I groups [83]. Annotations and conservation scores can be found in S7 Dataset. (C) The percent of 3' UTRs of transcripts encoding components of ETC complex I that have a Puf3 binding site sequence. A black vertical bar marks the background frequency of the Puf3 motif for each species. A significant *p*-value indicates that Puf3 motif matches are enriched in complex I encoding RNAs relative to all other transcripts. The *p*-value was computed using Fisher's exact test and is corrected for multiple hypothesis testing. The multi-subunit, transmembrane complex I was independently lost in several fungal lineages, and species lacking this complex are noted. Percentages and *p*-values can be found in S1 Dataset.

The conservation of the critical RNA recognition residues in Puf3 proteins from all eukaryotes, described above (S2 Fig, S2 Text), including the extended set of 80 fungi that we have analyzed in greater depth (S5 Fig), argues against the first model in which Puf3 proteins in the Leotiomyceta have evolved a novel sequence specificity. Nevertheless, we tested this model by experimentally determining the binding specificity of Puf3 from the Pezizomycotina species *Neurospora crassa*. We ectopically expressed *N*. *crassa* Puf3 protein fused to a tandem affinity purification tag (TAP-tag) in a *S*. *cerevisiae* strain missing endogenous Puf proteins, Puf1-5 (derived from 5Δ*pufs* strain [47]), and we identified the RNAs bound by *N*. *crassa* Puf3 (Materials and Methods). Sequence analysis of the RNAs associated with *N*. *crassa* Puf3 identified a uniquely enriched motif strongly matching the eight-nucleotide motif preferred by *S*. *cerevisiae* Puf3 (Fig 4A, S6 Fig, S3 Text). Outside of this core eight-nucleotide motif, the *N*. *crassa* Puf3 motif lacks the modest preference of *S*. *cerevisiae* Puf3 for a cytosine residue two nucleotides upstream of the UGUA [25,49]. Comparative analysis of the conserved Puf3 targets in Saccharomycotina suggests that this preference was acquired within the Saccharomycotina lineage (S7 Fig), possibly to reduce competition with other Puf proteins (see S16 Text).

As the Saccharomycotina Puf3 target orthologs in Leotiomyceta species lack the canonical Puf3 recognition element (Fig 3) and Puf3 in Leotiomyceta has maintained its sequence specificity (Fig 4), Leotiomyceta Puf3 proteins then presumably recognize a different set or sets of RNAs that acquired the Puf3 recognition sequence through evolution. In the following section we describe these putative Leotiomyceta Puf3 targets.

#### RNA targets of Leotiomyceta Puf3 are involved in distinct mitochondrial and non-mitochondrial functions

We used sequence comparisons and conservation in 42 Leotiomyceta species to infer the RNAs that are bound by Leotiomyceta Puf3 proteins. First, we had to identify orthologous sets of Leotiomyceta genes. We anchored this search with the *N*. *crassa* genome because it is the most thoroughly annotated among the Leotiomyceta genomes. To do this we carried out pairwise sequence comparisons between *N*. *crassa* and each of the other genomes to identify orthologous genes (i.e., each *N*. *crassa* protein and its orthologs across the other Leotiomyceta species). We then searched the 3' UTRs of each orthologous gene set for matches to the Puf3 motif UGUA[ACU]AUA, defining a score for Puf3 binding site conservation that reflects the prevalence of 3' UTRs with Puf3 binding sites and accounts for the relatedness of the species (Materials and Methods). A false discovery rate (FDR) for each ortholog set was obtained from the rank of its calculated conservation score relative to the conservation scores from 100 permuted Puf3 motifs. For comparison, we also identified a set of conserved Puf3 targets in Saccharomycotina fungi defined relative to *S*. *cerevisiae* RNAs, and we refer to this set as Saccharomycotina Puf3 targets or ancestral Puf3 targets (*n* = 276, ≤1% FDR; S4 Text provides further discussion and additional evidence that members of this conserved target set that were not identified as targets experimentally are indeed Puf3 targets).

Puf3 recognition sites were significantly conserved (≤1% FDR) in the 3' UTRs of 409 ortholog sets in the Leotiomyceta species. The identity and functional themes in this set of putative Leotiomyceta Puf3 targets have multiple and profound differences relative to the Puf3 targets in Saccharomycotina. Whereas the vast majority of Saccharomycotina Puf3 targets have mitochondrial functions (256 of 276 targets, 93%), only about one-fourth of the conserved Puf3 target RNAs in Leotiomyceta encode mitochondrial proteins (113 targets, according to *N*. *crassa* annotation from [83]). Thus, the Leotiomyceta have nearly 300 inferred Puf3 targets that function in non-mitochondrial processes, in contrast to the near universal mitochondrial annotation of the Saccharomycotina targets. Furthermore, although enrichment of RNAs with mitochondrial functions among the Leotiomyceta Puf3 targets was highly significant (Fig 4B, odds-ratio = 3.9, *p* = 10^−25^ by Fisher's exact test), and the overlap with Saccharomycotina Puf3 targets was also significant (13%, odds-ratio = 3.2, *p* = 10^−6^ by Fisher's exact test), the Leotiomyceta Puf3 target set included only 26 of the 202 Saccharomycotina Puf3 targets that have orthologs in *N*. *crassa* and included 87 mitochondrial targets not observed in Saccharomycotina.

To understand the distinctions between mitochondrial targets of Puf3 in Leotiomyceta and Saccharomycotina, we determined within which of the 36 functional categories of mitochondrial genes [83] the Leotiomyceta Puf3 mRNA targets fall. We found remarkable enrichment for components of a particular mitochondrial protein complex, the ETC complex I; 27 of the 33 RNAs encoding ETC complex I subunits contained conserved Puf3 binding sequences (Fig 4B, odds-ratio = 107, *p* = 10^−30^ by Fisher's exact test). The frequency with which Puf3 binding sites were found in ETC complex I RNAs was significantly enriched in 33 out of 44 Pezizomycotina species, including the "basal" species *A*. *oligospora* (Fig 4C). In the remaining 11 species, Puf3 sites still occurred more frequently than expected by chance in ETC complex I RNAs (i.e., odds-ratio > 1 for all 11 species and *p* = 0.001 by two-sided binomial test). These data suggest that in the common ancestor of all Pezizomycotina species analyzed here the Puf3 ortholog bound RNAs encoding ETC complex I components.

The Leotiomyceta Puf3 targets were also significantly enriched for other mitochondrial categories not enriched in the Saccharomycotina targets: genes involved in amino acid metabolism (odds-ratio = 4.8, *p* = 0.005) and those categorized as "other" under import and biogenesis (odds-ratio = 4.8, *p* = 0.005) (S5 Table contains results for all mitochondrial subsets; *p*-values were Bonferroni corrected for testing of 36 subsets).

As the Leotiomyceta Puf3 targets contained 296 targets not annotated as mitochondrial, we searched for other themes among these genes using the annotations of *S*. *cerevisiae* orthologs. Despite the large number of targets, we found only a modest enrichment for the broad category of "membrane" (odds-ratio = 2.3, *p* = 0.0001 after Bonferroni correction for testing all gene ontology [GO] categories) whereas the majority of targets do not connect to a common, known functional theme. Understanding the selective advantages conferred by the conserved interactions with Puf3, in this large set of genes without known functional commonalities, is an important challenge (see Summary and Implications).

#### Evolutionary reprogramming of post-transcriptional regulation between Puf proteins: Pezizomycotina Puf4 binds >150 mRNAs orthologous to Puf3 targets in Saccharomycotina

The Saccharomycotina Puf3 targets encode particular mitochondrial proteins involved in multiple aspects of mitochondrial organization and biogenesis. The results presented above lead us to a parsimonious model in which the ancestral Puf3 gained these RNAs as targets in an ancestor to Saccharomycotina and Pezizomycotina and subsequently lost its interaction with these RNAs in an ancestor of Leotiomyceta species. We explored what happened to the post-transcriptional regulation of this set of related RNAs in the Leotiomyceta species, and specifically whether the coordinated regulation of these mRNAs might have been preserved via interactions with an alternative RNA binding protein or whether the coregulation of these RNAs was lost or reconfigured.

**Fig 5:**
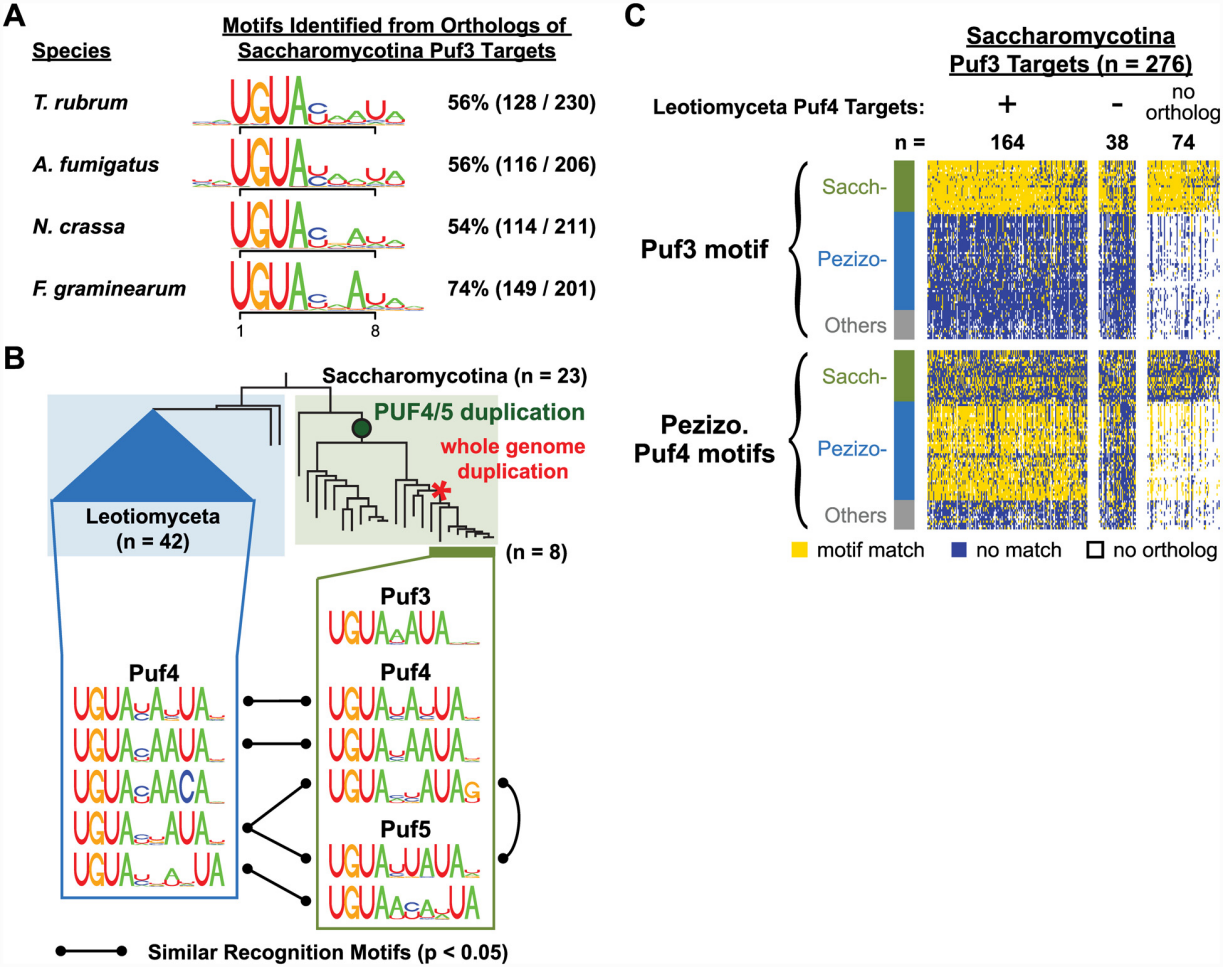
Evidence for Pezizomycotina Puf4 binding to RNA orthologs of Saccharomycotina Puf3 targets. (A) Motifs identified by the motif discovery program REFINE [61] using the 3' UTRs of orthologs of conserved Saccharomycotina Puf3 targets. Representatives spanning the Leotiomyceta lineage are shown. Numbers on the right indicate how many orthologous RNAs of Saccharomycotina Puf3 targets contain a match to the identified motif. All significant motifs found across the 80 fungi are displayed in S8 Fig, a summary of significance testing can be found in S11 Table, and position frequency matrices can be found in S5 Dataset. (B) Characterization and comparison of Puf binding specificity in Leotiomyceta and Saccharomycotina. Saccharomycotina Puf motifs were identified from *S*. *cerevisiae* Puf targets [25] and their orthologs in the seven closely related species up to *S*. *castelli*. The Pezizomycotina Puf motifs were identified from orthologs of conserved Saccharomycotina Puf3 targets in Leotiomyceta species. Our approach involved first identifying ten nucleotide sequences (containing UGUA at the 5' end and six other nucleotides) that help discriminate a Puf protein's RNA targets from its nontargets. These informative sequences were then clustered and assigned into groups (Materials and Methods). A black line between motifs indicates similarity in the non-UGUA positions as identified by MotifComparison [84]. For simplicity, a motif can only make one connection to another protein’s collection of motifs, and the motifs with higher similarity were given priority. Position frequency matrices can be found in S5 Dataset. (C) Heatmaps showing the prevalence of RNAs containing sequences recognized by Puf3 or Pezizomycotina Puf4. Each row represents results from one species, and each column represents an ortholog set. Ortholog sets for conserved Saccharomycotina Puf3 targets are shown and divided into groups based on whether the ortholog set is among the Leotiomyceta Puf4 targets. The third group contains Saccharomycotina Puf3 targets that did not have an ortholog in *N*. *crassa*. Complete motif search results can be found in S8 Dataset.

If another protein were to maintain coordinated post-transcriptional regulation of these RNAs, a distinct sequence element corresponding to the binding site of this hypothetical regulator might be a shared feature of these RNAs, discoverable via bioinformatic analysis. We therefore applied the motif finding program REFINE [61] to the Leotiomyceta orthologs of the Saccharomycotina Puf3 targets. We identified a motif in all Leotiomyceta species that was similar to, but distinct from, the Puf3 motif (see Fig 5A for representative motifs; all significant motifs are shown in S8 Fig). The tetranucleotide UGUA at the 5' end of the enriched sequence is a characteristic feature of sequences recognized by Puf family proteins [25,51], but the motif differs from the Puf3 motif in containing an extra nucleotide between the UGUA and the 3' end UA, resulting in a motif nine nucleotides in length instead of eight as with Puf3's motif.

The predicted Puf binding motif resembles the sequence recognized by *S*. *cerevisiae* Puf4. Previous work suggested Puf4 in Pezizomycotina species binds a small but significant fraction of RNAs encoding mitochondrial proteins [60]. This previous work assumed that the binding specificity of Puf4 in Pezizomycotina species is the same as that of *S*. *cerevisiae* Puf4 [60], but two observations suggested that this assumption may not hold. First, sequences matching the motifs that we identified from de novo comparative sequence analysis are found in the majority of the Leotiomyceta orthologs of the Saccharomycotina Puf3 targets (Fig 5A), whereas the previous work assuming a conserved Puf4 recognition motif found putative Puf4 binding sites in only ~20% of these RNAs. Second, Pezizomycotina Puf4 is orthologous to both *S*. *cerevisiae* Puf4 and Puf5, yet *S*. *cerevisiae* Puf4 and Puf5 binding specificities are distinct. Puf4 and Puf5 resulted from a gene duplication that we have dated to an early ancestor of nearly all Saccharomycotina species (S17 Text), and Puf4 and Puf5 in Saccharomycotina could have experienced changes in RNA sequence recognition subsequent to this duplication, rendering Saccharomycotina Puf4 recognition partially distinct from that of Pezizomycotina Puf4. Indeed, the following analyses provide evidence for such distinctions in binding specificities.

We carried out additional bioinformatic analyses to learn more about the RNA binding specificity of Pezizomycotina Puf4. Because Puf proteins can recognize RNA through multiple binding modes that are not best represented by a single motif [51,53,85], we developed a procedure that identifies enriched ten-nucleotide sequences that start with the canonical UGUA and collapses the sequences into a collection of motifs, instead of just one motif, that together represent the RNA binding specificity (Materials and Methods). This procedure yielded five motifs for Pezizomycotina Puf4 that represent sequences enriched within the 3’ UTRs of Pezizomycotina orthologs of Saccharomycotina Puf3 targets (Fig 5B and S11 Fig). Applying this procedure to characterize *S*. *cerevisiae* Puf3, Puf4, and Puf5 specificity yielded one motif for Puf3 and multiple motifs for Puf4 and Puf5 (Fig 5B and S11 Fig). Each of the motifs identified for the *S*. *cerevisiae* Puf3, Puf4, or Puf5 protein is supported by previous experimental data [51,85], demonstrating that our procedure can reveal RNA interaction information that would otherwise be obscured by a single motif representation.

Each of the five Pezizomycotina Puf4 motifs identified using our approach above shares a significant similarity with motifs recognized by *S*. *cerevisiae* Puf4 and/or Puf5, which are both orthologs of Pezizomycotina Puf4 (Fig 5B). In contrast, apart from the canonical UGUA core element, none of the motifs matched the *S*. *cerevisiae* (or *N*. *crassa* (Fig 4A)) Puf3 motif. Thus, this comparison provides evidence that Pezizomycotina Puf4 recognizes, at a minimum, a large subset of the ancestral Puf3 targets. This comparison also suggests that *S*. *cerevisiae* Puf4 and Puf5 specificity each became restricted to recognize distinct sequences after the gene duplication (S16 Text).

With reasonable confidence in our assignment of sequence motifs recognized by the Pezizomycotina Puf4 orthologs, based on the analyses described above and in S12 Fig, we wanted to determine the subset of former Puf3 targets co-opted by Puf4. To accomplish this, we defined a set of conserved Leotiomyceta Puf4 targets using the above motif criteria (*n* = 605, ≤1% FDR, Materials and Methods), and we compared it to the conserved Saccharomycotina Puf3 targets (Fig 5C). Of the 276 Saccharomycotina Puf3 targets, 202 have an ortholog in *N*. *crassa*, and 164 (81%) of those with orthologs are conserved as Puf4 targets in Leotiomyceta (Fig 5C). These results indicate that the Puf4 ortholog in Leotiomyceta binds a majority of the RNAs that, in Saccharomycotina, are bound by Puf3 and suggest that these RNAs may have been reprogrammed as a large set.

#### Evolutionary transition of the regulation of a large set of mitochondria-related genes from Puf3 to Puf4

To better reconstruct the evolutionary history of the reprogramming of Puf3 and Puf4 targets that accompanied the divergence of the Saccharomycotina and Pezizomycotina lineages, we looked specifically for species that might represent an intermediate state.

**Fig 6:**
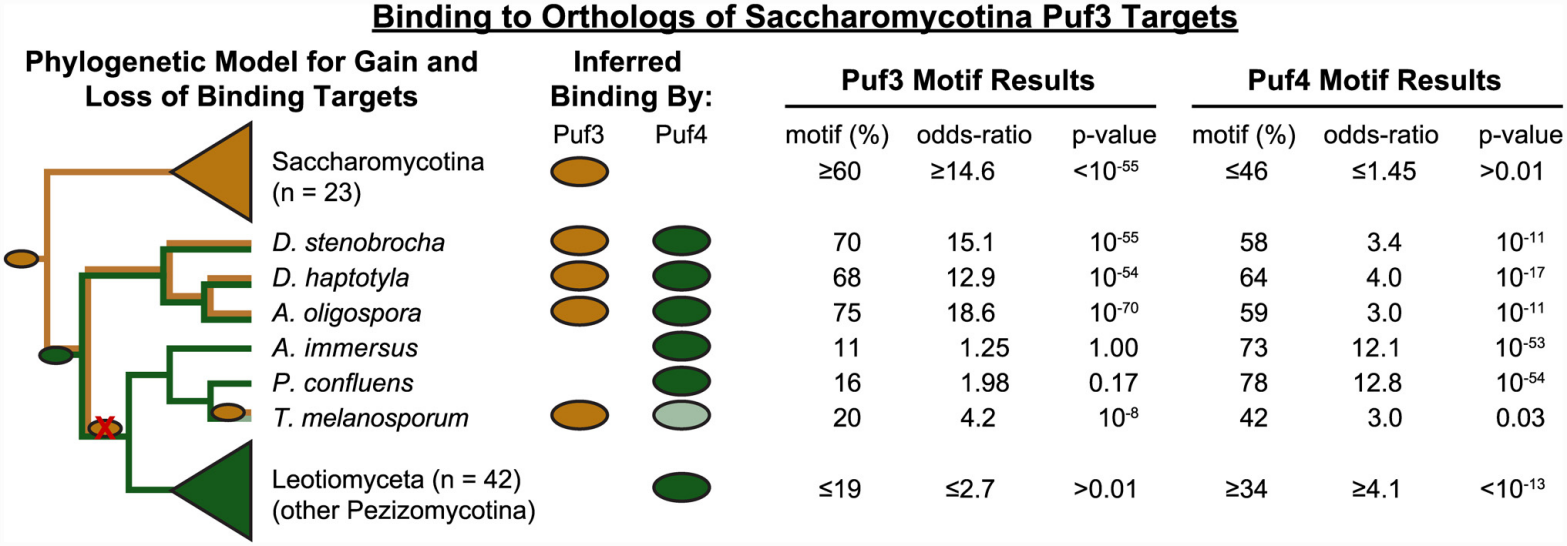
Inferring when Puf4 acquired RNA targets related to Saccharomycotina Puf3 targets. Prevalence of putative Puf3 and Puf4 binding sites in the orthologs of Saccharomycotina Puf3 targets in early diverging Pezizomycotina species. A putative binding site is inferred based on the presence of sequences matching the Puf3 or the Pezizomycotina Puf4 motif. Listed under motif results is the percentage of RNAs with a binding site sequence in the 3' UTR. The odds-ratio represents the enrichment of orthologs of Saccharomycotina Puf3 target RNAs containing a binding site sequence compared to the prevalence in all other RNAs. The *p*-value was computed using Fisher's exact test and was Bonferroni corrected. A colored oval in the "Inferred Binding By" column indicates significant enrichment of Puf3 or Puf4 sites in each species. The light green oval for Puf4 indicates borderline significance. The cladogram on the left represents a parsimonious model with color along a branch indicating the binding of Puf3 (gold) or Puf4 (green) to the orthologs of Saccharomycotina Puf3 targets (see S21 Fig for an alternative model). A colored oval within the cladogram denotes a gain in binding, and a red X over an oval denotes a loss of binding. Literature results were used to derive the relationships of species within Orbiliomycetes [86,87] and Pezizomycetes [88–91].

We tested each sequenced fungal genome for enrichment of the Pezizomycotina Puf4 motif in the 3' UTRs of RNAs orthologous to Saccharomycotina Puf3 targets. As expected from our previous results, matches to the Pezizomycotina Puf4 motif were enriched in all Leotiomyceta species, and the Puf4 motif was not enriched in the Puf3 target RNAs in Saccharomycotina species (S14 Fig). Surprisingly, both Puf3 and Puf4 motifs were enriched in RNAs related to Saccharomycotina Puf3 targets in the basal species *A*. *oligospora* (Fig 6 and S14 Fig). This overlap suggests that regulation by Puf3 and Puf4 is not mutually exclusive.

To clarify the history of Puf-RNA interaction changes, we analyzed genome and protein sequences for four additional species in early diverging classes, two within Orbiliomycetes and two within Pezizomycetes. This allowed us to assess the full set of sequences for these classes available when our analyses commenced. In the Orbiliomycetes species *Drechslerella stenobrocha* and *Dactylellin*a *haptotyla*, as in *A*. *oligospora*, putative binding sites for both Puf3 and Puf4 were enriched in the RNAs orthologous to Saccharomycotina Puf3 targets (Fig 6). However, in the Pezizomycetes species, *Ascobolus immersus* and *Pyronema confluens*, in contrast to what we found for *T*. *melanosporum*, only Puf4 sites, and not Puf3 sites, were significantly enriched in RNAs orthologous to Saccharomycotina Puf3 targets, suggesting still additional complexity in the natural history of this regulatory program in Pezizomycetes (Fig 6).

The prevailing consensus phylogenetic model has the Orbiliomycetes branching prior to the Pezizomycetes [88,92–94]. Mapping our results onto this framework leads to the conclusion that acquisition of Puf4 regulation of some of the Saccharomycotina Puf3 targets preceded the loss of Puf3 regulation of this gene set. This model implies that the loss of Puf3 regulation of this set of genes occurred within the Pezizomycotina lineage (Fig 6). Other models are possible, but all models require a complex sequence of evolutionary events affecting interactions of Puf3 and Puf4 with this RNA target set (S21 Fig, S14 Text).

#### Puf3 and Puf4 confer distinct selective advantages in Orbiliomycetes

Two general mechanisms can account for evolutionary changes: drift and selective pressure. For the Puf protein targets described above, sequence changes in a common set of at least 164 mRNAs switched them from interacting with one Puf protein to another. Under a model of neutral drift, Puf3 and Puf4 are predicted to have redundant advantages in the regulation of the conserved targets. For a model involving selection, Puf4’s function with respect to the conserved targets would be distinct from Puf3’s function (i.e., Puf3 and Puf4 provide distinguishable selective advantages in the regulation of the common mRNAs).

The models of drift and selection for evolutionary changes make distinct predictions for how Puf3 and Puf4 binding sites have evolved and how the binding sites are distributed among the conserved targets in Orbiliomycetes. Under a model of drift wherein Puf3 and Puf4 are redundant, the fitness cost of losing a Puf3 site from a given RNA would be reduced (and its likelihood thereby increased) by the presence of a Puf4 site in that same RNA, and vice versa. Under a model in which Puf3 and Puf4 provide completely independent functions, the fitness cost, and thus the probability of observing the loss of a binding site for one of these factors in a given RNA, should be independent of the presence or absence of binding sites for the other factor in the same RNA. We inferred the rates of binding site gain and loss across target 3' UTRs in Orbiliomycetes and found that the rate of Puf3 binding site gain or loss was not different when a Puf4 binding site was already present, and vice versa, counter to the prediction for neutral drift (S11 Text, S16 Fig).

Extending the above predictions for how binding sites evolve, a model for redundancy between Puf3 and Puf4 also predicts that fewer of the conserved targets will contain binding sites for both Puf3 and Puf4 than expected by their prevalence. When formalized in a statistical model, Puf3 and Puf4 binding sites will display a negative interaction (i.e., using Puf3 and Puf4 sites to predict which RNAs are conserved targets will not be additive, see model 3 in Table 1). This model can be compared to an alternative model in which Puf3 and Puf4 provide independent advantages in the regulation of these mRNAs; the statistical model formalized from this evolutionary model does not include an interaction term (see model 2 in Table 1). We used stepwise logistic regression to find the most parsimonious model that effectively explains which mRNAs are conserved targets. We compared the fits to the models separately for each Orbiliomycetes species (*n* = 3), and we found that the best model for all three Orbiliomycetes species was one in which Puf3 and Puf4 independently contributed to the prediction of which mRNAs are conserved targets (Table 1). In other words, the additional parameter invoking dependence between Puf3 and Puf4 sites did not significantly help account for the data (Table 1).

**Table 1:** Summary of stepwise logistic regression tests for dependence between Puf3 and Puf4 in Orbiliomycetes.

Species	Model^a^	ΔX^2^	*p*-Value	Variable	β (SE)	Odds-ratio [95% CI]
***A*. *oligospora***	0			Constant	−3.5 (0.083)***	
	1	336	3.70E-75	Constant	−5 (0.19)***	
				Puf3 Motif	3.3 (0.21)***	28 [19, 43]
	**2**	**30.6**	**3.10E-08**	**Constant**	**−5.4 (0.21)** ***	
				**Puf3 Motif**	**3.2 (0.21)** ***	**25 [17, 39]**
				**Puf4 Motif**	**0.98 (0.18)** ***	**2.7 [1.9, 3.8]**
	3	1.63	0.2	Constant	−5.2 (0.25)***	
				Puf3 Motif	2.9 (0.3)***	19 [11, 35]
				Puf4 Motif	0.55 (0.38)	1.7 [0.8, 3.7]
				Puf3 Motif x Puf4 Motif	0.55 (0.44)	1.7 [0.74, 4.2]
***D*. *stenobrocha***	0			Constant	−3.5 (0.088)***	
	1	302	1.47E-67	Constant	−5 (0.2)***	
				Puf3 Motif	3.3 (0.23)***	28 [18, 45]
	**2**	**19.6**	**9.60E-06**	**Constant**	**−5.3 (0.22)** ***	
				**Puf3 Motif**	**3.2 (0.23)** ***	**24 [16, 39]**
				**Puf4 Motif**	**0.83 (0.19)** ***	**2.3 [1.6, 3.4]**
	3	0.0247	0.88	Constant	−5.3 (0.27)***	
				Puf3 Motif	3.2 (0.32)***	23 [13, 45]
				Puf4 Motif	0.78 (0.4)	2.2 [0.96, 4.8]
				Puf3 Motif x Puf4 Motif	0.072 (0.46)	1.1 [0.44, 2.7]
***Dactyl*. *haptotyla***	0			Constant	−3.5 (0.083)***	
	1	260	1.37E-58	Constant	−4.7 (0.16)***	
				Puf3 Motif	2.9 (0.19)***	17 [12, 25]
	**2**	**48.4**	**3.39E-12**	**Constant**	**−5.2 (0.19)** ***	
				**Puf3 Motif**	**2.6 (0.19)** ***	**14 [9.7, 21]**
				**Puf4 Motif**	**1.3 (0.19)** ***	**3.5 [2.4, 5.1]**
	3	2.62	0.11	Constant	−5 (0.22)***	
				Puf3 Motif	2.3 (0.3)***	9.6 [5.3, 17]
				Puf4 Motif	0.84 (0.32)*	2.3 [1.2, 4.3]
				Puf3 Motif x Puf4 Motif	0.64 (0.4)	1.9 [0.87, 4.2]

We used stepwise logistic regression to find the most parsimonious model that effectively explains which mRNAs are conserved targets. We used the presence or absence of a Puf binding sequence (“Puf3 Motif” or “Puf4 motif”) to predict the outcome of being an ancestral Puf3 target (defined here as the intersection of Saccharomycotina Puf3 targets with Leotiomyceta Puf4 or Puf3 targets). The term "(Puf3 Motif x Puf4 Motif)" in model 3 represents a statistical interaction that accounts for a dependence between Puf3 and Puf4. Modeling was performed with the R function glm() with family = “binomial.” The accepted model is highlighted in bold for each species, which is model 2 (~Constant + Puf3 Motif + Puf4 Motif) in all three species.

^a^ 0: ~ Constant, 1: ~ Constant + Puf3 Motif, 2: ~ Constant + Puf3 Motif + Puf4 Motif, 3: ~ Constant + Puf3 Motif + Puf4 Motif + (Puf3 Motif x Puf4 Motif)

*** *p* < 0.0001

* *p* < 0.01

To summarize, a large set of RNAs that share common functional characteristics appear to be regulatory targets of Puf3 in the Saccharomycotina lineage and targets of Puf4 in the sister lineage Pezizomycotina. In the earliest branch of the Pezizomycotina lineage, these RNAs are targets of both Puf3 and Puf4. The binding sequences of Puf3 and Puf4 appear to have conferred independent selective advantages during the divergence of the Orbiliomycetes lineage, suggesting that the proteins mediate distinct regulatory programs.

#### Differential change in RNA abundance upon Puf4 protein loss suggests that the regulatory logic of Puf4 network in *N*. *crassa* is different from the logic of the Puf3 network in *S*. *cerevisiae*

The distribution of Puf3 and Puf4 binding sites in Orbiliomycetes suggests that Puf evolution was subjected to a change in selection. The distinct advantage of Puf4 could have been gained in the ancestor of Pezizomycotina, thereby also affecting Puf evolution in Leotiomyceta, or it could have been gained specifically in Orbiliomycetes. As Puf3 binding sites have largely been lost in the conserved targets in Leotiomyceta, Puf3 appears to have lost its selective advantage in their regulation. This regulation could have been replaced by a functionally redundant Puf4 or replaced by Puf4 with a separate function.

The simplest drift model for conversion to Puf4 targets in Leotiomyceta predicts that Puf4 would have the same affect on these targets as Puf3 after takeover. *S*. *cerevisiae* Puf3 mediates the decay of its target RNAs, in part by recruiting the CCR4-NOT deadenylase complex [95,96]. In an *S*. *cerevisiae* Puf3 knockout, the abundance of Puf3 target RNAs is higher than in wild-type cells with Puf3 present [25,97]. If Puf3’s mRNA decay function has been conserved and shared with Puf3 in ancestor of Pezizomycotina, then Puf4 is predicted to share this function under a model of redundancy.

**Fig 7:**
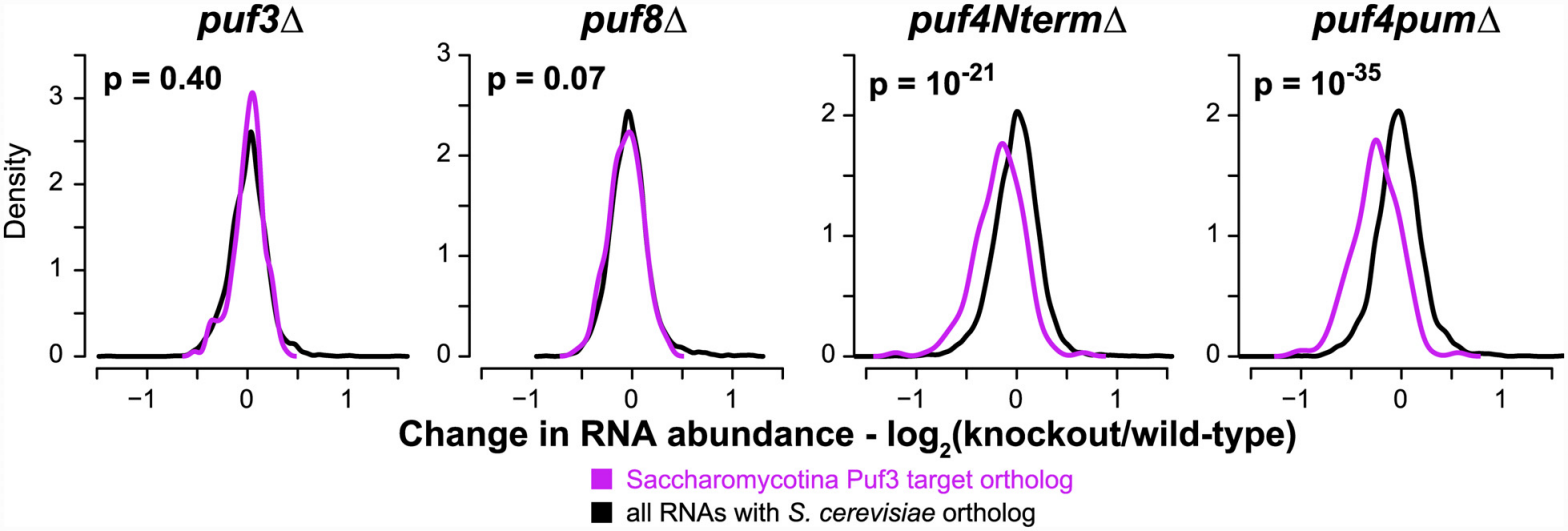
Gene expression profiling of Puf mutants in *N*. *crassa*. Density plots displaying changes in RNA abundance for orthologs of Saccharomycotina Puf3 targets (purple) or orthologs of any *S*. *cerevisiae* protein (black). The *p*-values were computed using the two-sided Wilcoxon test. Note that the partial gene knockouts of Puf4 each exhibited a significant growth defect of ~5% relative to a wild-type strain, signifying a functional defect despite part of the gene remaining in each strain (S13 Fig, S7 Table). Gene expression data can be found in S10 Table.

To probe Puf4's regulation of the orthologs of Saccharomycotina Puf3 targets, we performed gene expression profiling in Puf knockouts in *N*. *crassa* using DNA microarrays. We profiled strains with partial gene knockouts of Puf4, one strain with the C-terminus encompassing the Puf RNA binding domain removed (*puf4pumΔ*) and another with the sequence coding for the N-terminus removed (*puf4NtermΔ*); strains with gene knockouts for Puf3 or Puf8 were used as controls. (Puf8 appears to have been derived from a Puf3 duplication in a common ancestor of Pezizomycotina and Saccharomycotina, and is predicted to recognize sequences containing UGUA; see S5 Text) RNA was isolated from *N*. *crassa* strains growing as vegetative mycelia and compared to RNA isolated from a wild-type strain grown in parallel. In the Puf3 and Puf8 knockouts, there was no significant change in the relative abundance of RNAs orthologous to Saccharomycotina Puf3 targets (Fig 7). However, in each of the Puf4 mutant strains, the relative abundance of these RNAs was selectively altered (Fig 7). This collective change provides additional strong evidence that, in Pezizomycotina, Puf4 regulates the set of RNAs related to the Saccharomycotina Puf3 targets. Importantly, mutation of Puf4 in *N*. *crassa* led to a decrease in the abundance of these RNAs, in contrast to the increase observed when Puf3 is knocked out in *S*. *cerevisiae* [25,97].

Although the detailed working of the regulatory networks remains to be elucidated, the opposite effects on RNA target levels indicate that the evolutionary rewiring of targets of Puf3 and Puf4 was accompanied by significant change in the logic of the regulatory program. The simplest model for the timing of this change is that Puf4 gained its distinct regulatory advantage in the ancestor of Pezizomycotina.

The changes in regulators of the conserved Puf mRNA targets were further accompanied by diversification in the RNAs that Puf3 binds (e.g., addition of ETC I targets) and in the RNAs that Puf4 binds (see next section), perhaps altering the coordination of mitochondrial regulation.

#### Additional events in the evolution of Pufs and their RNA targets in fungi

**Fig 8:**
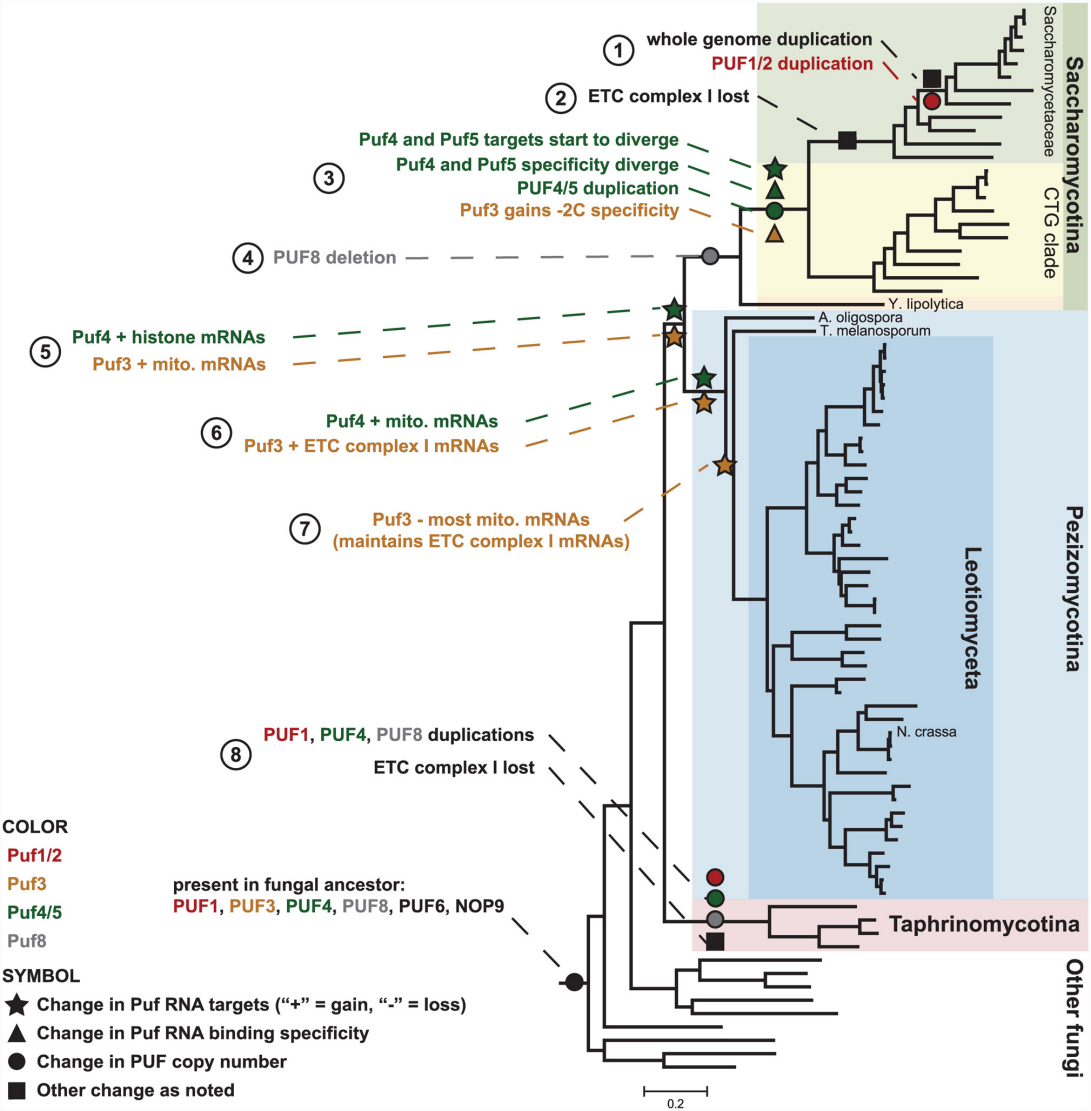
Phylogenetic model for changes in Puf proteins and their RNA interactions throughout fungal evolution. Symbols indicate the type of change, and colors link the indicated event to a particular Puf protein.

The changes in Puf3 and its mRNA targets that we document above are only a subset of the changes that have occurred for Puf proteins in fungi. Fig 8 summarizes a model for events in the evolution of Puf proteins and their targets in fungi, as derived from our analyses and experiments. This figure highlights how gene expression programs diversified as the result of changes in Puf proteins and their mRNAs targets.

Our investigation of Puf3 uncovered links to Puf4. In Pezizomycotina, Puf4 binds hundreds of RNAs distinct from the conserved Saccharomycotina Puf3 targets. We describe these distinctions in S6 Text and S23 Fig, and we describe a speculative model for the transition from Puf3 to Puf4 in S14 Text.

In S16 Text, we document the history of acquisition and loss of Puf genes in fungi. We also relate changes in Puf genes to changes in the regulatory specificity of Puf proteins in fungal evolution.

In S7 Text, we further explore the natural history of Puf4 and its paralog Puf5 in Saccharomycotina. We provide evidence that mRNA targets and binding specificity diverged after the Puf4/Puf5 duplication (S7 Text, S24 Fig, S16 Text). Puf4 and Puf5 may have maintained functionally distinct subsets of the ancestral Puf4 targets (S9 Text). Yet, despite divergence in binding specificity and substantial changes in targets, Puf4 and Puf5 also bind to a small, common set of mRNAs that include those encoding histone proteins. The interactions between the Puf4 protein and RNAs encoding histone proteins appear to have been conserved through much or all of fungal evolution, dating back 750 million years or more, even while many other changes were occurring in Puf4 targets (S8 Text, S24 Fig). It is possible that Puf4 (and other RNA binding proteins) serves multiple functions within the same organism, even organisms as simple as fungi. It is also possible that individual RNA binding proteins serve to functionally connect different RNAs with different functions (see Summary and Implications).

### Summary and Implications

The rewiring of gene expression programs plays a major role in evolution and adaption of new species. Considerable effort has been dedicated to analyzing evolutionary changes in transcription factors and in their targets (see [8–13] for reviews), but far less is known about rewiring at the level of RNA and its binding proteins. We surveyed the evolutionary changes in one family of RNA binding proteins and their cognate recognition elements, broadly across eukaryotes and more deeply within fungi (Figs 2 and 8).

Our evidence points to the existence of mRNA targets of Puf proteins that have been maintained for hundreds of millions of years (Figs 2 and 8). Overlaid on this conservation are numerous and remarkable changes in the number of Puf proteins, their specificity, their regulatory output, and their targets. The substantial changes in Puf proteins and targets over evolution followed by long periods of high conservation together underscore the importance of these protein–RNA interactions for organismal adaptation and fitness. Puf proteins represent only ~1% of all RNA binding proteins [15], but similar rewiring of interactions between RNA binding proteins and their targets has likely been a pervasive adaptive strategy throughout evolution.

The highly conserved binding specificity of Pufs suggests that the conserved interactions between each protein and its many mRNA targets place a large constraint on binding specificity. A change in binding specificity thus marks a period of innovation in the gene regulatory program. In the time following Puf4 duplication in Saccharomycotina, the binding specificity of the paralogs (Puf4 and Puf5) became restricted with respect to the ancestral specificity and diverged with respect to each other (Figs 5B and 8 #3). Analogous binding and catalytic promiscuity has been proposed to have been present in ancestral enzymes that later duplicated and specialized [98–103]. Our phylogenetic studies and evolutionary model suggest specificity changes, potential physical origins (S15 Text), and support the idea that aspects of the evolution of RNA binding proteins and their targets proceeded via early promiscuous binding proteins that later underwent gene duplication and subdivision of the ancestral RNA recognition.

The observations that the conserved RNA targets of each Puf protein share functional themes and that a set of functionally-related RNA targets can switch in concert from specific interactions with one RNA-binding protein to another, provide strong support for the notion that RNA binding proteins play an important biological role in organizing and coordinating aspects of gene expression [18,20,21]. Concerted evolutionary changes in mRNAs encoding mitochondrial organization and biogenesis proteins involved hundreds of RNA sequences, placing the same set of orthologous genes in distinct fungal lineages under the regulation of Puf3, Puf4, or both proteins. The evolutionary history of changes in their post-transcriptional regulation, suggested by this analysis, provides strong evidence for the fitness advantage of coordinating the regulation of distinct sets of genes and may harbor clues to the selective pressures that led to changes in the regulatory program.

Whereas essentially all of the inferred RNA targets of Puf3 in Saccharomycotina are transcribed from nuclear genes encoding proteins with mitochondrial functions, not every ortholog of each gene we identified as encoding a Puf3 target in the Saccharomycotina contains a recognizable Puf3 binding site. It is possible that the fitness advantage (or disadvantage) conferred by Puf3 regulation of each of the individual genes in this set is often small enough to allow for considerable genetic drift within the lineage. The evolutionary plasticity that this would allow might help account for the distinct but overlapping functional and cytotopic themes shared by the targets of a given Puf protein in distinct species and lineages.

Although Saccharomycotina Puf3 is essentially monogamous in its relationship to RNAs with mitochondrial functions and has served as a “poster child” for RNA binding protein-based coordination of gene expression, the targets of other Puf proteins are functionally and cytotopically more promiscuous. For example, Saccharomycotina Puf4 binds RNAs encoding histone and nucleolar proteins, while Pezizomycotina Puf4 binds RNAs encoding histone and mitochondrial proteins. The RNA targets of Leotiomyceta Puf4 also encompass a broader array of cellular functions relative to the Saccharomycotina Puf3 targets, including targets with roles in energy metabolism (through the ETC and TCA cycle) and the proteasome. We do not know whether these multiple themes arise because RNA binding proteins help coordinate and integrate cell status and signals between different systems or whether they represent multiple uses of the same protein for independent functions [104–106]. It is also possible that limitations in our understanding of and ability to identify biological function could account for our inability to map mRNA targets to function in a 1:1 fashion.

Evolutionary changes in regulatory RNA–protein interactions are likely to have many similarities to the changes observed in the evolution of transcriptional control (S12 Table). By comparing the changes in transcriptional regulation (as reflected by gain or loss of specific promoter elements) and post-transcriptional regulation (as reflected by gain or loss of Puf-protein recognition elements in the corresponding transcripts) in sets of functionally related genes that share features of both transcriptional regulation and putative Puf-protein regulation, we found that the timing and likely the consequences of evolutionary changes at these two levels of regulation of a common set of genes can be distinct (S13 Text). RNA–protein interactions can thus provide an additional and independently evolvable infrastructure by which global gene expression networks can be orchestrated and reconfigured to generate phenotypic diversity.

By using systematic investigation of evolutionary changes in gene expression programs to enrich the pictures of these programs acquired from years of detailed studies of “representative” model organisms, we found compelling evidence for dramatic changes in the gene expression program at the level of RNA–RNA binding protein interactions during fungal evolution. Mapping evolutionary changes in post-transcriptional regulation can provide new insights into the makeup, logic, and malleability of gene expression programs, and may contribute to our ability to engineer new phenotypes by rewriting or de novo design of post-transcriptional programs.

## Materials and Methods

### Retrieving Data for Species Represented in the InParanoid Database

Protein sequence files and SQL tables containing ortholog information were downloaded from InParanoid [107] (version 7.0, http://inparanoid.sbc.su.se/). Genome sequences for each species were downloaded in July 2010 from the sources listed in S3 Table.

### Identifying Puf Proteins Based on Similarity to Known Puf Proteins

We used a two-step BLASTP search to identify putative Puf proteins in each species. A custom BLAST database was created for each species' protein sequences using makeblastdb (part of the blast+ package from NCBI). In the first step, the sequences of the Pum domains of *S*. *cerevisiae* Pufs 1–6 (Puf1:557–913, Puf2:511–872, Puf3:513–871, Puf4:539–888, Puf5:188–596, Puf6:133–483) and the complete protein sequence of *S*. *cerevisiae* Nop9 were used as a query to search for similar protein sequences in each species using blastp (NCBI BLAST version 2.2.23 [108–110]), using an E-value cutoff of 10^−5^. Sequences identified in the first step were then used to search for additional Puf proteins in a second step, also with an E-value cutoff of 10^−5^. In the second step only the parts of the protein sequence identified in the first step as having significant similarity to *S*. *cerevisiae* Pum domains were used. If more than one of the query sequences from the first step was similar to a searched sequence, the similar sequence of longest length was kept.

Results from the first round yielded near-complete coverage of known Pufs from *Caenorhabditis elegans*, *A*. *thaliana*, and *O*. *sativa* (12/12, 24/26, and 17/19, respectively) [38,111–113]. The second round yielded one more known Puf from *A*. *thaliana* and two from *O*. *sativa*. Additionally, putative Pufs in these organisms were found in both rounds (one from the first round, two from the second). Two of the three additional putative Pufs contained one or more Puf repeats according to the SMART annotation tool [114,115], suggesting these hits are real Puf proteins. As our next step was to classify Puf proteins, we aimed for high coverage at the expense of a small fraction of false positives.

### Classifying Puf Proteins as Orthologs to *S*. *cerevisiae* Puf Proteins or *N*. *crassa* Puf8

We classified Puf proteins as orthologs to each of the *S*. *cerevisiae* Puf proteins or to *N*. *crassa* Puf8, a previously uncharacterized Puf that we identified and named. We chose *S*. *cerevisiae* because of our focus on fungi in this work, and the results suggest that *S*. *cerevisiae* Pufs well represent the diversity of Puf proteins found across eukaryotes, with the exception of *N*. *crassa* Puf8, which our initial phylogenetic analysis suggested was deleted in an ancestor of *S*. *cerevisiae*. More than 90% of the eukaryotic Pufs and 98% of the fungal Pufs were classified as orthologs to *S*. *cerevisiae* Pufs or *N*. *crassa* Puf8.

We classified Puf proteins based on a combination of information: reciprocal best BLAST hits, the pattern of amino acids predicted to contact RNA bases within each Puf repeat, and phylogenetic analysis. For reciprocal best BLAST, we checked each Puf against *S*. *cerevisiae* and *N*. *crassa* Pufs. A protein was tentatively assigned as an ortholog if it was a reciprocal best BLAST hit to at least one *S*. *cerevisiae* or *N*. *crassa* Puf protein, and the reciprocal best BLAST hit did not disagree between the *S*. *cerevisiae* Puf and its *N*. *crassa* ortholog.

A Puf protein was also tentatively assigned as an ortholog to *S*. *cerevisiae* Puf1/Puf2, Puf3, Puf4/Puf5, or *N*. *crassa* Puf8 based on predicted RNA-contacting amino acids. RNA-contacting amino acids are highly conserved but are different in distantly related Pufs. The *S*. *cerevisiae* Puf1 and Puf2 have similar RNA-contacting amino acids, and those in *S*. *cerevisiae* Puf4 and Puf5 are identical to each other so this type of classification cannot distinguish between these two proteins. Outside of these two pairs, the RNA-contacting amino acids are sufficiently different to allow this classification. We performed this classification manually and note any differences between the protein and its tentatively assigned ortholog with respect to these amino acids in S1 and S2 Tables.

Puf proteins were assigned a final ortholog if the BLAST-based classification or the RNA contact classification identified a tentative ortholog and so long as the assignment from the two classification methods did not disagree. Any Pufs not assignable by these criteria were subject to a phylogenetic analysis. Protein sequences for *S*. *cerevisiae* Pufs, *N*. *crassa* Pufs, and the unassigned Pufs were aligned as a group using MUSCLE [116,117] in Geneious (using default settings). Columns with more than 50% gaps were stripped, and a maximum likelihood tree was built using PhyML [118,119] implemented through Geneious (WAG substitution model, 8 substitution rate categories, best of NNI [Nearest Neighbor Interchange] and SPR [Subtree Pruning and Regrafting] search). Many of the remaining Pufs were classified based on this tree (S1 and S2 Tables). In some cases, we referred back to the pattern of RNA-contacting amino acids to inform our decision (see notes in column “unknownGroup_ML tree” in S1 and S2 Tables)

The relationship of a group of Puf proteins from worms, including *C*. *elegans* Fbf-1 and Fbf-2, remained ambiguous. This relationship was resolved by considering which Pufs were likely present in the ancestor of these species. These worm Pufs tend to have eight predicted Puf repeats and are closest to Puf3 and Puf4 among *S*. *cerevisiae* Pufs. We inferred that the Puf4 gene was deleted in an ancestor to metazoans and the choanoflagellate *Monosiga brevicollis* and therefore could not be orthologous to these worm Pufs. In contrast, Puf3 is inferred to be present in the ancestor of these worms, and we had already identified other Puf3 orthologs in these species. We assigned the worm Pufs as orthologs to Puf3 under a model that Puf3 underwent several duplications (duplication of Puf3 and duplication of duplicates) along the worm lineage with subsequent divergence of many of the duplicates.

### Multiple Sequence Alignments, Calculation of Percent Identity, and Definition of Puf Repeats

For S2 and S5 Figs, protein sequences were aligned using MUSCLE [116,117], as implemented through the program Geneious and using default settings. For calculating percent identity of residues, all columns containing gaps in *S*. *cerevisiae* Puf3 were removed. Percent identity was calculated as the percent of residues matching the most abundant residue within each column of the alignment. Puf repeats were defined using the SMART annotation tool [114,115]. The *S*. *cerevisiae* Puf3 repeats are residues 538–573, 574–609, 610–645, 646–681, 682–717, 718–752, 760–795, and 809–844. The multiple sequence alignments and calculated percent identities are presented in S2 Dataset.

### Extracting 3' UTR Sequences for Species Found in the InParanoid (v7) Database and Fungi Species

Protein sequences were mapped back to the respective genome to identify coding sequence boundaries using standalone BLAT v34 [120] (with parameters–q = prot–t = dnax). BLAT output was processed to identify for each query the hit with the smallest discrepancy (defined as the smallest difference between query and match lengths). We assessed overall performance by calculating the average percent discrepancy and average coverage for the best hits. The median across all InParanoid species for average coverage was 99.8%, and the average discrepancy was 0.2%. Eighty of the InParanoid species had proteins mapping back to the genome with an average coverage >99% and a discrepancy <1%. *G*. *gallus* had the lowest average coverage (90.6%), and *G*. *lamblia* had the highest average discrepancy (12.5%). All 80 fungi had an average coverage of >99% and a discrepancy of <1% (median: 99.9% coverage, 0.1% discrepancy). The 500 nucleotides downstream (3' on the coding strand) of each best BLAT hit were extracted as the 3' UTR.

### Testing Puf3/Pumilio Target Conservation across Eukaryotes

We used a custom Perl script analogous to Fastcompare [63,74,75] to search for the Puf3 motif in orthologous sequence sets of two species, yielding a 2 x 2 contingency table of the number of sets that have a motif match in both species, in only one of the species, or in neither of the species. We searched 3' UTRs of orthologs identified by InParanoid in 99 eukaryote species [107]. The significance of ortholog sets that both have motif matches was computed by the hypergeometric test. To control for sequence similarity expected between closely related species, we repeated the search using permutations of the Puf3 motif (e.g., UA[ACU]AUAGU) and used the hypergeometric *p*-value as a score to rank the Puf3 motif against all of its permutations (*n* = 1119). We report a *p*-value if the overlap between two species for the Puf3 motif is significant after correcting the hypergeometric *p*-value for multiple hypothesis testing (*p* < 0.05 after Bonferroni correction) and if the Puf3 motif is ranked in the top 1% (i.e., empirical *p* < 0.01 for comparison against all permutations).

### Inferred Eukaryote Phylogeny

Phylogenetic trees were inferred using methods similar to those used previously [121–123]. To identify proteins whose sequence has preserved the underlying phylogenetic signal, we searched for proteins that contained an ortholog to a human protein in at least 90 of the 99 species investigated herein, and that within each species contained at most two orthologs to a human protein (1:1 or 2:1 orthologs); we identified a total of 53 sets of proteins meeting this criteria, and within each set, most species only had one ortholog for each human protein used (1:1 orthologs). Each set of orthologs was multiply aligned using standalone MUSCLE [116,117] (version 3.8.31 with default settings). The alignments were concatenated, and during the concatenation process, we kept only the first ortholog encountered for each species and added a sequence of gaps where an ortholog was not found. Columns containing more than 5% gaps were removed, yielding a final alignment with 27,239 columns. A tree was inferred by maximum likelihood using standalone PhyML [118,119] (version 20120412, parameters -d aa -b 1 -m WAG -o tlr -s SPR—n_starts 10 -v e -c 8). For the phylogeny displayed, the descendants of a node were collapsed if a branch length from the ancestor node to one of the descendant nodes (i.e., the internode distance) was greater than 0.65. The branches that were collapsed largely reflect uncertainty in the relationship of species diverging earliest within eukaryotes and uncertainty about the root of the tree. The final phylogeny displayed generally agrees with the literature consensus, and points of disagreement did not affect our conclusions. For example, *N*. *vectensis*, *T*. *adhaerens*, *Capitella sp*. *I*, *H*. *robusta*, and *L*. *gigantea* are proposed to be basal metazoan species in the literature consensus, and the worms (nematode, trematode) are proposed to be grouped with the insects to the exclusion of vertebrates. The final phylogeny with species names can be found in S20 Fig. The multiple sequence alignment and a newick-formatted tree can be found in S3 Dataset.

### Inferred Fungal Phylogeny

For fungi we identified 20 sets of proteins that across all species were 1:1 ortholog to an *S*. *cerevisiae* protein. We allowed *A*. *macrogynus* to have multiple orthologs to each *S*. *cerevisiae* protein because its genome contains many duplicated genes. Each set of orthologs was multiply aligned using standalone MUSCLE [116,117] (version 3.8.31 with default settings). The alignments were concatenated, and during the concatenation process, we kept only the first *A*. *macrogynus* sequence encountered. Columns containing gaps were removed, yielding a final alignment with 4,251 columns. An initial maximum likelihood tree was inferred using standalone PhyML [118,119] (version 20120412, parameters -d aa -b 100 -m WAG -o tlr -s SPR—n_starts 10 -v e).

The initial fungi phylogeny placed *A*. *oligospora* (a species within Orbiliomycetes) and *T*. *melanosporum* (a species within Pezizomycetes) together. We suspected that this was a long-branch artifact, as it disagreed with previous studies that used a higher sampling of species within Orbiliomycetes and Pezizomycetes [88,92–94]. The previous studies placed Orbiliomycetes and Pezizomycetes as separate lineages that diverged the earliest within Pezizomycotina. Nevertheless, one study [88] disagreed with others [92–94] in terms of which lineage is most basal (i.e., earliest diverging). We chose to constrain the topology to place Orbiliomycetes (*A*. *oligospora*) as the most basal lineage followed by Pezizomycetes (*T*. *melanosporum*) then the rest of Pezizomycotina. This order is consistent with two of the three studies that inferred phylogenies using multiple gene sequences [92,94] and the study using the "ultrastructure" character of different species [93]. The alternative topologies (the one from the literature and our unconstrained topology) lead to models in which an additional loss event is required to account for the Puf3 pattern and thus would alter details of our models but not the overall conclusions drawn (S21 Fig).

We constrained the tree topology and optimized the branch lengths and rate parameters using PhyML (with parameter -o lr). The resulting tree was rooted between the species within Chyridiomycota (*A*. *macrogynus*, *B*. *dendrobatidis*, *S*. *punctatus*) and all other fungi, but this root should be viewed as a hypothesis. The final phylogeny used for fungi contains discrepancies with previously published trees, but the discrepancies occur at parts of the tree where the literature itself is inconsistent. As the alternative topologies would not affect our conclusions, we did not attempt to resolve these discrepancies. The multiple sequence alignment and a newick-formatted tree can be found in S3 Dataset.

### Retrieving Sequence Data and Identifying Orthologs in Fungi

Protein and genome sequence data were retrieved from the sources listed in S4 Table. We used InParanoid v4.1 [107,124–126] (default settings with no outgroup species) to identify orthologs of *S*. *cerevisiae* or *N*. *crassa* proteins in each of the other fungi. Tables containing orthologs can be found in S4 Dataset.

### *N*. *crassa* Strains and Linear Race Growth Assays

*N*. *crassa* strains were obtained from the Fungal Genetics Stock Center [127]. Strains were the wild-type *N*. *crassa* 74-OR23-1VA (FGSC #2489) [128] and knockout strains of the gene NCU06199.2 (PUF1, FGSC #13194), NCU06511.2 (PUF3, FGSC #13380), NCU01774.2 (part of PUF4 removes N-terminus of protein, FGSC #14089), NCU01775.2 (part of PUF4 removes Pumilio domain, FGSC #14547), NCU01760.2 (PUF8, FGSC #15499), or NCU06199.2 (PUF1, FGSC #13194 [129]. The Puf4 gene was originally annotated as two separate genes, so the *Neurospora* knockout collection had a separate knockout strain for each of the original annotated genes. One strain has a deletion of the sequence encoding the 5' portion of the mRNA including the predicted natural start codon. The other deletion strain is missing the sequence encoding the 3' end of the mRNA, including the sequence that encodes the Pumilio RNA binding domain and the natural translation stop codon. The knockout strains were homokaryons and of mating-type A. Strains were preserved long-term by resuspending conidia in sterile 7% milk, mixing with an equal volume of 50% glycerol, and storing at −80°C.

Agar "race" tubes were prepared in 25 mL pipets (Falcon 352575). Pipets were filled with 13 mL of autoclaved medium containing 1X Vogel's Medium, 1.5% agar (BD Difco 214530), and 2% of a carbon source (sucrose, glucose, maltose, or glycerol). Medium was allowed to solidify on a flat surface.

Each *N*. *crassa* strain was streaked onto 3 mL agar slants made with Vogel's Medium with 2% sucrose and grown for 7–10 days at room temperature with constant exposure to indoor light. Conidia were obtained by adding 1 mL of water to each slant, vortexing, and extracting the liquid. Resuspended conidia (20 μL) were used to inoculate a race tube through the hole made at the top of the pipet using a heated needle. Tubes were incubated at 37°C in the dark for 24 h to allow the strains to reach a maximal growth rate, and then measurements were taken twice daily until mycelium growth neared the end of the tube. Growth rates were calculated as a weighted average of the rates obtained between every two measurements, where the weight is the fraction of time elapsed between two given measurements. The calculated rates from this approach displayed lower variability than those calculated from linear regression. Measurements were obtained from two replicates for sucrose and maltose conditions, four for glycerol, and five for glucose. Statistical significance was assessed by the two-sided *t* test.

### Gene Expression Profiling in *N*. *crassa*

Conidia were extracted in water from *N*. *crassa* strains streaked onto 3 mL agar slants made with Vogel's Medium with 2% sucrose and grown for 7–10 days at room temperature with constant exposure to indoor light. An estimate of conidia concentration was made by taking a sample, diluting 1:40 into water, and measuring the optical density at 530 nm. An OD_530_ of 0.25 was found to correspond to approximately 10^8^ conidia/mL in the undiluted sample. Conidia were added to a final concentration of 10^6^ conidia per mL into 25 mL of Vogel's Medium with 2% glucose as the carbon source. Cultures were shaken at 200 rpm in a 30°C incubator with lights on. After 8 h ~100% of cells exhibited hyphal growth with most having germ tube lengths between 50 and 400 μm. At this point, mycelia were collected by vacuum filtration. Material was scraped from the filter and placed into tubes containing 0.5 mL of buffer AE (50 mM sodium acetate, 10 mM EDTA), 33.3 μL of 25% SDS, and 0.5 mL of acid phenol:chloroform pH 4.5 (Ambion AM9720) then inverted to mix and flash frozen in liquid nitrogen.

RNA was isolated by hot acid phenol/chloroform extraction. Samples were placed at 65°C in a thermomixer shaking at 1,400 rpm for 10 min, vortexed for 10 s, then placed back in the thermomixer for another 5 min. Samples were cooled on ice for 5 min, then spun at 12,000 rpm in a microcentrifuge for 15 min. The aqueous phase was extracted and placed into a 2 mL phase-lock gel tube. Two more extractions with acid phenol:chlorofom were performed, followed by an extraction with chloroform. RNA was precipitated by adding one-tenth volume of 3 M sodium acetate, mixing, and then adding 1 volume of isopropanol. Samples were mixed and placed at −20°C for at least an hour. Samples were spun for 20 min at top speed in a microcentrifuge. The RNA pellet was washed with ice-cold 75% ethanol then air dried for 10 min before being resuspended in 100 μL of water. RNA yields were ~250 μg, and 15 μg of RNA were used in each reverse transcription reaction (see "Sample processing for microarrays").

### Sample Processing for Microarrays

RNA was reverse transcribed in the presence of 5-(3-Aminoallyl)-dUTP. Sample RNA (filled with water to 13.8 μL) was mixed with 1 μL of control RNA (Ambion AM1780) and 2 μL of N9 and dT20VN primers (each at 2.5 μg/μL). This mixture was heated to 70°C for 2 min then cooled to 4°C. Six microliters of 5x 1st Strand Buffer (Invitrogen 18080–085), 1.2 μL of 25x dNTP/aminoallyl-dUTP mix (Ambion AM8439), 3 μL of 0.1 M DTT, 1 μL SuperaseIn (Ambion AM2696), and 2 μL of Superscript III (Invitrogen 18080–085) was added as a 13.2 μL master mix, and reverse transcription performed at 42°C for 2 h. RNA was then hydrolyzed by addition of 15 μL of 1 M NaOH and heating to 70°C for 15 min. The sample was neutralized by addition of 15 μL of 1 M HCl and 10 μL of sodium acetate pH 5.2. cDNA was purified using the Qiagen MinElute kit and eluted from the column with 20 μL of 10 mM sodium phosphate, pH 8.5.

Experimental sample cDNA was labeled with Cy5 dye while cDNA made from the wild-type strain was labeled with Cy3 dye (GE Healthcare Life Science RPN5661). A tube of NHS-monoester Cy dye was resuspended in 60 μL of DMSO, and 20 μL was used for each sample to be labeled. Coupling was performed at room temperature in the dark for 1–2 h. The labeling reaction was quenched by addition of 9 μL of 3 M hydroxylamine and incubation for 15 min. Labeled cDNA was purified using the Qiagen MinElute kit.

Labeled cDNA from experimental and wild-type samples were mixed (27 μL total) along with 6 μL of 20X SSC (1X SSC is 150 mM NaCl, 15 mM sodium citrate at pH 7.0), 2 μL of Qiagen buffer EB (10 mM Tris-HCl, pH 8.5), 3 μL of 10 μg/μL polyA RNA (Sigma P4303), 1 μL of 1 M Hepes-NaOH, pH 7.0, and 1 μL of 10% SDS. This 40 μL probe mixture was heated at 95°C for 2 min then centrifuged for 5 min.

### Oligonucleotide Microarrays and Their Post-processing

*N*. *crassa* microarrays were obtained from the Fungal Genetics Stock Center [127] and were printed on aminosilane-coated glass as part of the *Neurospora* functional genomics project [130]. *S*. *cerevisiae* microarrays were obtained from the Stanford Functional Genomics Facility and were printed on epoxysilane-coated glass.

Arrays were postprocessed on the day of hybridization. Arrays were rehydrated by placing slides face down over 50 mL of 0.5X SSC in a humidity chamber (Sigma H6644) for 30 min and were then snap dried on a 70–80°C inverted heat block for 5 s.

For *N*. *crassa* arrays, the DNA was crosslinked to the slide using 600 millijoules of UV energy in a Stratalinker. The aminosilane surface was blocked by incubation for 35 min in a solution of 5X SSC, 1% SDS, and 1% w/v of Blocking Reagent (Roche 11096176001) at 60°C. Arrays were washed twice in water for 2 minutes at room temperature then dried by centrifugation.

For *S*. *cerevisiae* arrays, the epoxysilane surface of the slides was blocked by incubation in a solution of 1 M Tris-HCl, pH 9.0, 100 mM ethanolamine, and 0.1% SDS for 20 min at 50°C. Arrays were washed twice in high-quality water for 1 min then dried by centrifugation.

### Microarray Hybridization, Washing, Scanning, and Data Extraction

Probe mixture containing labeled cDNA was hybridized to postprocessed microarrays using the MAUI hybridization system (BioMicro) at 65°C for ~16 h. The MAUI mixer was removed from the microarray while submerged in a warm solution of 2X SSC and 0.01% SDS. The array was then placed in a 2X SSC solution at room temperature until all arrays were ready for washing. Arrays were washed with 2X SSC and 0.05% SDS at 65°C for 5 min with agitation, then at room temperature with agitation in 2X SSC for 1 minute, another 2X SSC wash for 2 min, 1X SSC for 2 min, and 0.2X SSC for 2 min. The arrays were dried by centrifugation in a low-ozone environment.

Microarrays were scanned using an AxonScanner 4000B and GenePix 6.0 software (Molecular Devices). PMT levels were set to maximize signal in each channel and only saturate a few spots. Spots were located using the GenePix software with some manual adjustment and flagging. Spots were then auto-flagged as bad if they met any of the following criteria: greater than 10% of the spot pixels were saturated in either channel, the spot contained 12 pixels or less, the R^2^ for the fit between Cy5 (red) and Cy3 (green) pixel intensities was less than 0.6, or if in either channel the signal intensity minus the local background was less than three times the standard deviation of the local background.

Array data were exported in a GenePix Results file (.gpr) and further processed and analyzed within the R statistical environment. After data were loaded into R, a spot was filtered if signal intensity was not 2-fold over background in both channels for *N*. *crassa* gene expression experiments or 1.5-fold over background for *S*. *cerevisiae* affinity purifications. For features passing flagging and filtering, we calculated the ratio between the experiment and reference channels as log_2_(red signal − red background) / (green signal − green background)). Log_2_ ratio data for each experiment were mean centered and then replicate spots on the array were averaged.

The microarray data can be found in S9 and S10 Tables and also have been submitted to the Gene Expression Omnibus (GEO) under the accession number GSE50997.

### Yeast and Plasmid Construction

*HIS4* was amplified by PCR from the *S*. *cerevisiae* strain BY4741 and used to replace *his4-539* by homologous recombination in the 5Δ*pufs* strain [47] (named yRP1253 or yWO24, genotype is MAT*α*, *his4-539*, *leu2-3*,*112*, *lys2*, *trp1-1*, *ura3-52*, *cup1*::*LEU2/PM*, *puf1*::*Neo* ^*r*^, *puf2*::*TRP1*, *puf3*::*Neo* ^*r*^, *puf4*::*LYS2*, *puf5*::*URA3*). Transformation was performed using the lithium acetate method. Transformants were selected by growth on SD − His (synthetic defined without histidine) plates, yielding 5Δ*pufs his4-539*::*HIS4* (named GHY001). *HIS3* in this strain was then replaced with *HPH*, which confers resistance to hygromycin B. *HPH* with flanking *HIS3* homologous arms was produced by fusion PCR [131]. Transformants were selected on YPD plates containing 500 μg/mL hygromycin B. Correct integration was tested by restreaking colonies on another YPD + hygromycin plate (+ control) and a SD − His plate (−control). This transformation produced 5Δ*pufs his4-539*::*HIS4 his3*::*HPH* (named GHY002).

The *N*. *crassa PUF3* coding sequence (NCU06511) was made by gene synthesis (GenScript) and placed into pUC57. The TAP-tag [132], which includes two copies of the IgG binding domain of *Staphylococcus aureus* protein A, was added in-frame to the 3' end of the *N*. *crassa PUF3* gene by fusion PCR. The PCR product, which included XbaI and SmaI restriction enzyme sites on its ends, was digested and ligated into the yeast expression vector p413ADH (ATCC 87669) [133] at the XbaI and SmaI sites. The ligation reaction mixture was used to transform *Escherichia coli*, and p413ADH plasmid containing PUF3-TAP was isolated. This plasmid was then used to transform GHY002. Transformants were selected on SD − His plates, and protein expression was verified by western blot.

### Affinity Purification of Puf Proteins in *S*. *cerevisiae*

Affinity purifications were performed in parallel and in triplicate. GHY002 not expressing TAP tag protein (used as a "mock"), GHY002 expressing *N*. *crassa* Puf3-TAP protein, and *S*. *cerevisiae* Puf3-TAP [132] (derivative of BY4741, Thermo Scientific YSC1177) were grown as 250 mL cultures in SD − His (+His for GHY002 alone) media to midlog phase (OD_600_ of 0.6–0.9). Cells were collected by centrifugation at 5,000 xg, and cell pellets were chilled on ice. The cell pellet was washed twice in 5 mL of ice-cold buffer A (50 mM Hepes-KOH pH 8.0, 140 mM KCl, 1.8 mM MgCl_2_, 0.1% NP-40 alternative, and 0.2 mg/mL heparin). The cell pellet was resuspended in 0.5 mL of buffer B (buffer A plus 1 μg/mL pepstatin and leupeptin, 2.5 μg/mL aprotinin, 1 mM PMSF, 0.5 mM DTT, and 100 units/mL Murine RNase Inhibitor (NEB M0314)). Cells were lysed using 0.65 mL of glass beads (Biospec 11079105) and a Beadbeater (Biospec) in four 1 min cycles with 1 min on ice between cycles. Beads were removed by centrifugation at 1,000 xg, and the lysate was cleared by centrifugation at 8,000 xg for 5 min at 4°C. The supernatant was extracted, and the total protein concentration was adjusted to 15 mg/mL by dilution with buffer B.

Magnetic beads were prepared for use in Protein A purification. Rabbit IgG (Calbiochem 401590) was made free of detectable RNase activity by spin column purification (Sartorius VS-ARAMAXIK) then biotinylated (Pierce 21329) and bound to Dynabeads MyOne Streptavidin C1 magnetic beads (Invitrogen 65002). Biotinylated-IgG (100 μg) and 250 μL of magnetic beads were used for each affinity purification.

Lysate (1 mL at 15 mg/mL) was added to beads after its buffer was removed. Lysate and beads were mixed for 2 h at 4°C. Depleted supernatant (100 μL) was saved for reference RNA. The beads were washed 1x with 1.5 mL of buffer B for 15 min and 3x with 1.5 mL of buffer C (buffer B plus 10% glycerol) for 15 min at 4°C then resuspended in 300 μL of buffer C and flash frozen in liquid nitrogen.

RNA was isolated from affinity purification samples and the depleted supernatants, 35 μL of 10% SDS (1% final) and 7 μL of 0.5 M EDTA (10mM final) was added to each sample, and samples were adjusted to 350 μL total with water. RNA was purified by successive extractions with hot acid phenol:chloroform, phenol:chloroform, and chloroform alone. For the depleted supernatants, RNA from 100 μL of the final aqueous phase was then purified using Qiagen RNeasy columns by adding one-tenth volume sodium acetate, mixing with 5 volumes of Qiagen buffer PB, loading onto the column, washing with buffer PE, and eluting off the column with 50 μL of warm water. For the affinity purification samples, RNA from the aqueous phase was isopropanol precipitated, washed with 75% ethanol, dried, and resuspended in 20 μL of water.

Two-thirds of the affinity purification sample RNA (~0.5–2 μg) was used for reverse transcription; 12 μL (~8–10 μg) of depleted supernatant ("reference") RNA was used (see "Sample processing for microarrays").

### Motif Searches Using REFINE or FIRE

We used the programs FIRE [82] and REFINE [15,61] to search for enriched sequence patterns in sets of 3' UTRs. For each search, 3' UTRs for a given set of transcripts were compared to a background set of 3' UTRs (e.g., 3’ UTRs of orthologs to *S*. *cerevisiae* proteins). FIRE (v1.1) was run with parameters (—exptype = discrete—dodna = 0—seqlen_rna = 500—nodups = 1—kungapped = 6—gap = 0–4). REFINE (v0.1), which uses dust and MEME (v4.7.0) [134–137], was run with defaults except the parameter for the minimum number of significant k-mer sites for target sequences to be kept was set to 1 (CT = 1). The background mononucleotide frequencies used in MEME were calculated from the complete set of 3' UTRs sequences used as input. For the IP data, the *S*. *cerevisiae* 3' UTRs of targets of *S*. *cerevisiae* Puf3 or *N*. *crassa* Puf3 were searched using both REFINE and FIRE. Both programs returned motifs that resembled the canonical Puf3 recognition element; the results from FIRE are displayed in Fig 4A as its motifs had lower *p*-values than REFINE based on the hypergeometric test. For other searches, only REFINE was used. Files containing position frequencies (PFM) for each motif can be found in S5 Dataset.

The statistical significance of motifs returned by REFINE was compared to results generated by shuffling the target assignment of input sequences and running the motif search. One hundred permutations were performed for each real motif search, and the hypergeometric *p*-value of the top motif from each permutation was compared to the motifs found from real data. A motif was considered significant if its *p*-value was lower than all *p*-values found from permutations (i.e., *p* < 0.01). A summary of the permutation results can be found in S11 Table.

### GO Term Searches and Correction for Multiple Hypothesis Testing

GO term searches were performed using "GOstats" [138] within Bioconductor [139] in R and using annotations from the *S*. *cerevisiae* database in "org.Sc.sgd.db". For each comparison we used set *S*. *cerevisiae* genes that had orthologs in the respective species as a background (i.e., used only genes with data). *p*-Values from GO term searches were Bonferroni corrected for multiple hypothesis testing by multiplying the *p*-value by 7,097, which represents the number of GO terms within molecular function, biological process, and cellular component. The results of all GO term searches can be found in S6 Dataset. *p*-Values in these files have not been Bonferroni corrected.

### Characterizing Puf Binding Specificity Using a Series of Motifs

We identified a series of motifs that represent each Puf protein’s RNA binding specificity using the following three steps: identifying the top informative UGUA-based 10mers (i.e., 10mers that discriminate putative targets from nontargets), identifying a cutoff for informative 10mers to keep, and clustering these 10mers into groups.

To identify informative UGUA-based 10mers, we first selected an in-group and out-group. For the *Saccharomyces* Pufs, the in-group consisted of experimentally identified *S*. *cerevisiae* Puf targets and its orthologs in post-WGD species (*S*. *cerevisiae*-RM11-1, *S*. *paradoxus*, *S*. *mikatae*, *S*. *kudriavzevii*, *S*. *bayanus*, *S*. *castellii*, *C*. *glabrata*). For Pezizomycotina Puf4, the in-group consisted of orthologs of conserved Saccharomycotina Puf3 targets in all Pezizomycotina species except *A*. *oligospora* and *T*. *melanosporum*. The out-group consisted of orthologs that were not in the in-group. We searched the 3' UTRs with every possible UGUA-based 10mer (UGUANNNNNN) and tallied how many 3' UTRs in the in-group did or did not have a match and how many in the out-group did or did not have a match. From the resulting 2 x 2 contingency table, we calculated the mutual information for each 10mer, which is a measure of how much information the presence or absence of a 10mer match contributes to the classification of the two groups.

$$Mutual\;Information=\underset{i,j}{\sum^{}}p_{i,j}\times {\text{log}}_{2}\frac{p_{i,j}}{p_{i}\;\times \;p_{j}}$$ (1)

*p*_*i*,*j*_ represents the joint probability while *p*_*i*_ and *p*_*j*_ represent the marginal probabilities. The 10mer with highest mutual information was kept. We repeated the search to identify 250 total 10mers, and after each round we masked 3' UTRs that had already been accounted for by a kept 10mer.

To identify a cutoff for informative 10mers, the 10mers were ranked based on which round of the search they were found, and we calculated the local slope (centered window of 11 points) of the false positive rate (FPR) against the true positive rate (TPR) as in a ROC (Receiver Operator Characteristic) curve. The cutoff used was the first point before which the local slope drops below one (FPR > TPR). For the Pezizomycotina Puf4 search, the local slope took a sharp decline around the 100th 10mer but did not fall below one until the 137th 10mer, so we used a more stringent cutoff to include only the top 100 10mers.

We then identified subsets of informative 10mers that shared a common sequence pattern. To group 10mers based on similarity to each other, we made a network where a node represents a 10mer and an edge represents 10mers that have a Hamming distance of one (one substitution between two 10mers). This network was visualized in Cytoscape 2.7.0 [140] and organized using the yFiles Organic layout. From the network display, we manually placed the 10mers into groups if a sequence pattern was shared with nearby nodes (S11 Fig). Although this was a manual procedure, we clearly identified the unifying pattern within each cluster of 10mers (S11 Fig).

### Representing and Comparing Puf Binding Specificity

For comparing motifs between Puf proteins, we represented each Puf protein’s specificity as a sequence logo [141] based on the number of matches to each 10mer in that group. We only used matches to in-group sequences and matches found as part of the iterative search. For calculating conservation scores, we derived a regular expression to represent each group. A nucleotide was included at a position in the regular expression if it was found in more than 10% of the sequences. The regular expressions are TGTA[ACT]ATA (Puf3), TGTA[ACT]A[ACT]TA or TGTA[ACT][ACT]ATA (Saccharomycotina Puf4), TGTA[AT][CT][AT][AT]TA or TGTA[CGT]TATA (Saccharomycotina Puf5), and TGTA[ACT]A.TA or TGTA[ACT].ATA or TGTA[CT]AACA or TGTA[ACT].[AT].TA (Pezizomycotina Puf4). In a regular expression, a period (.) permits any nucleotide to be present at that position, and a position within brackets (e.g., [AT]) permits the nucleotides indicated. For the Puf4 regular expressions, the group that required A at positions 6 and 7 (TGTA[ACT]AATA) was split into the groups that required A at only one of positions (TGTA[ACT]A.TA or TGTA[ACT].ATA). Files containing the results of each motif search can be found in S7 Dataset.

To statistically assess the similarity between motifs, we used position frequency matrices from the six positions downstream of UGUA as input to MotifComparison (*p*-BLiC, 100 shuffles, no shift permitted between matrices) in the MotifSuite [84]. MotifComparison calculated the *p*-value of the Bayesian Likelihood 2-Component (BLiC) score [142], and we considered two motifs to be similar if *p* < 0.05.

Motifs are displayed as sequence logos [141], and images were made using a version of the seqLogo package in R that was modified to accommodate U in the logos.

### Calculating Conservation Scores and Identifying Significantly Conserved Ortholog Sets

We calculated a conservation score to represent the prevalence of 3' UTRs that have a match to a given motif within a set of orthologs (e.g., an *S*. *cerevisiae* protein and its orthologs in Saccharomycotina species). For each set of orthologs, we first assigned each species a presence (1) call if the 3' UTR had a motif match or an absence (0) call if it did not. Species without an ortholog were removed from the tree. If a species had more than one ortholog, we searched the 3' UTRs of all of them and assigned a presence call if any had a motif match. The conservation score (CS) for the ancestor of A and B is defined as follows:

$$CS_{A,B}=P_{A}\times BL_{A}+P_{B}\times BL_{B}$$ (2)

The conservation score is the weighted sum of branch lengths over which matches to the motif (i.e., putative binding sites) are inferred to be present. This score helps to control for the uneven sampling of species within each phylogeny. The weight is the proportion (*P*) of the branch length (*BL*) over which a motif match is present. *BL*_*A*_ and *BL*_*B*_ represent the sum of branch lengths from the ancestor to the respective descendant(s). The proportion (*P*) is defined as follows:

$$P_{A}=\{\begin{array}{cc} 1, & if\;descendant\;is\;leaf\;and\;motif\;match\;present\\ 0, & if\;descendant\;is\;leaf\;and\;motif\;match\;absent\\ \frac{CS_{A1,A2}}{BL_{A1}+BL_{A2}}, & if\;descendant\;is\;internal\;node \end{array}$$ (3)

*A1* and *A2* represent the descendants of *A*. We assumed no change in state along each branch from descendant to ancestor if the descendant is an extant species (terminal node or leaf). If the immediate descendant is an internal node, we assumed that the proportion of the branch length spent with a motif match present between an ancestor and the immediate descendant is the same proportion that the descendants of that descendant spent with a motif match present. The conservation score was calculated recursively upwards from the terminal nodes.

To estimate FDRs, we calculated conservation scores (CS) using 100 permuted motifs (*pm*) and calculated the FDR for a given ortholog set *A* and real motif (*rm*) as follows:

$$FDR\left(A,rm\right)=\frac{\frac{1}{100}{\Sigma^{}}_{pm=1}^{100}{\Sigma^{}}_{i=1}^{n}\{\begin{array}{ll} 1, & if\;CS\left(i,pm\right)\geq CS\left(A,rm\right)\\ 0, & if\;CS\left(i,pm\right)<CS\left(A,rm\right) \end{array}}{{\Sigma^{}}_{i=1}^{n}\{\begin{array}{ll} 1, & if\;CS\left(i,rm\right)\geq CS\left(A,rm\right)\\ 0, & if\;CS\left(i,rm\right)<CS\left(A,rm\right) \end{array}}$$ (4)

where *i* represents a single ortholog set and *n* represents the total number of ortholog sets. The numerator represents the average number of ortholog sets with a conservation score from a permuted motif that are greater than or equal to the conservation score for ortholog set *A* from the real motif. The denominator represents the number of ortholog sets with a conservation score from the real motif that are greater than or equal to the conservation score for ortholog set *A* from the real motif. We permuted the position of the motifs, thereby maintaining the redundant information when a regular expression has more than one motif. For example, a regular expression with two motifs TGTAC and TGTAT could result in a permutation with CATGT and TATGT, where for instance the G in position 2 of both motifs is now moved to position 5 in the permuted motifs. Files containing calculated conservation scores and FDRs are in S8 Dataset.

Our definition of conserved targets has the potential to identify novel targets of Puf proteins. In S4 Text, we provide strong support for the identification of over 100 novel targets for Puf3, Puf4, and Puf5 in *S*. *cerevisiae*.

### Testing Conservation of Puf4 and Puf5 Binding to Histone Transcripts

Multiple genes often encode each core histone, and as our conservation analysis only reports one number per species, we wanted to test whether Puf4 and Puf5 sites were enriched in the ortholog set of each histone while accounting for the number of 3' UTRs we searched. We collected the orthologs of each *S*. *cerevisiae* histone protein and collapsed these into a set based on the histone type (Hta1 and Hta2 for H2A, Htb1 and Htb2 for H2B, Hht1 and Hht2 for H3, Hhf1, and Hhf2 for H4, Htz1 for H2A.Z). For each type of histone, we extracted and searched the 3' UTRs for a match to the appropriate Puf motif (Saccharomycotina Puf4, Saccharomycotina Puf5, or Pezizomycotina Puf4). A species was assigned a presence (1) or absence (0) call if at least one of the 3' UTRs for a given histone type had a motif match. For each lineage and histone type tested, we calculated a conservation score (See "Calculating conservation scores and identifying significantly conserved ortholog sets"). For a null distribution, we calculated conservation scores from searches using permuted versions of the Puf motif. Comparison of conservation scores from the permuted motifs to the conservation score using the real motif yielded an empirical *p*-value. We did not test a histone type and lineage if the maximum conservation score possible could not yield a significant *p*-value (i.e., if the test is statistically underpowered).

### Data Processing, Statistics, and Data Visualization

Custom Perl or R scripts were written for data processing, statistical testing, and data visualization as needed. We used Bioperl [143] for some input and output operations and for traversing phylogenetic trees. We used the R functions fisher.test() to calculate a *p*-value for Fisher's exact test and also report an odds-ratio, t.test() to calculate its *p*-value, phyper() to calculate a *p*-value for the hypergeometric test, fisher.exact() in the exact 2 x 2 package [144] to calculate confidence intervals for the odds-ratio, and binom.test() to calculate a *p*-value for the binomial test. Hypergeometric test *p*-values reported herein were calculated from the one-tailed test. Fisher's exact test is a two-tailed version of the hypergeometric test, and for reference can be calculated as follows:

$$p=\frac{\left(\begin{array}{c} a+b\\ b \end{array}\right)\left(\begin{array}{c} c+d\\ c \end{array}\right)}{\left(\begin{array}{c} n\\ a+c \end{array}\right)}\;=\frac{\left(a+b\right)!\;\left(c+d\right)!\;\left(a+c\right)!\;\left(b+d\right)!}{a!b!c!d!n!}$$ (5)

where *a*, *b*, *c*, and *d* represent the cells in a 2 x 2 contingency table, *n* is the sum of all cells, and $\left(\begin{array}{c} a+b\\ b \end{array}\right)$ represents a binomial coefficient. A binomial coefficient $\left(\begin{array}{c} n\\ k \end{array}\right)$ can be expressed as $\frac{n!}{k!\left(n-k\right)!}$.

## Supporting Information

S1 Dataset *p*-Values and fraction conserved to accompany Figs 3, 4C, and S14 Fig. (ZIP)

S2 Dataset Percent identities and multiple sequence alignments for *S*. *cerevisiae* Puf3 and orthologs. (ZIP)

S3 Dataset Phylogenetic trees and multiple sequence alignments for eukaryotes, fungi, and Pufs. (ZIP)

S4 Dataset Ortholog tables for *S*. *cerevisiae* and *N*. *crassa*. (ZIP)

S5 Dataset Position frequency matrices for motifs identified in affinity purifications and from orthologs of conserved Puf3 targets. (ZIP)

S6 Dataset Results of GO term searches using *S*. *cerevisiae* annotations. (ZIP)

S7 Dataset Matrices of results for motif searches across ortholog sets. (ZIP)

S8 Dataset Conservation scores and FDRs for each motif search and lineage. (ZIP)

S9 Dataset Results of testing for conservation of Puf3 RNA targets in eukaryotes. (ZIP)

S10 Dataset Results of motif searches in histone 3' UTR sequences. (ZIP)

S1 Fig Distribution of Puf proteins and events in their history across eukaryotes. Barplots display the number and types of Puf proteins identified for each eukaryote species. Events in Puf protein history are noted if supported by the number of Puf proteins or an absence in three or more species. "Sacch-" refers to Saccharomycotina fungi, "Peziz-" to Pezizomycotina fungi, and "C.g." to the *Caenorhabditis* genus of worms. The break noted in red removed 0.5 from the branch to *G*. *lamblia*. The full list of species represented is shown in S20 Fig. S1 Table contains information about the Puf proteins in each species.
(PDF)

S2 Fig Prevalence of Puf3 ortholog and conservation of its RNA binding specificity across eukaryotes. (A) Cataloging Puf genes in each of 99 different eukaryotic genomes. The number of Puf3 orthologs with perfectly conserved RNA-base-contacting amino acids is indicated in green, the number of all other Puf3 orthologs is indicated in dark gray, and the number of other Puf proteins is indicated in white. Species are ordered based on the phylogeny in Fig 2. Colored boxes below the *x*-axis also correspond to clades as labeled in Fig 2. S1 Table contains information about the Puf proteins in each species. (B) Percent identity of each amino acid in each Puf repeat, in Puf3 orthologs. Amino acids in green denote RNA-base-contacting residues. Percent identity was calculated based on multiple sequence alignment of orthologs defined as reciprocal best BLAST hits to *S*. *cerevisiae* Puf3 (*n* = 74). This approach avoids paralogs of Puf3 in a species that may have diverged following a duplication event. We calculated the percent identity of each set of residues that aligned to a *S*. *cerevisiae* Puf3 residue. Percent identities and conserved residues can be found in S2 Dataset. (C) Same as (B) but with percent identities plotted for the full-length *S*. *cerevisiae* Puf3. Residues in the Puf repeats are noted in red, and RNA-contacting residues are in green. (D) Amino acid percent identity displayed on structure of *S*. *cerevisiae* Puf3's RNA-binding domain. Structure displayed represents two different views of a space-filling model from PDB entry 3k49 [49]. The RNA molecule with sequence CCUGUAAAUA is indicated in green. The structures are oriented with the 5' end of the RNA and the C-terminus of the protein at the top as in Fig 1.
(PDF)

S3 Fig Conservation of Puf3 RNA targets using individual pairwise comparisons. (A) Histogram representing the distribution of hypergeometric *p*-values for the Puf3 motif and all 1,119 permutations in the comparison of *Homo sapiens* and *Mus musculus*. The *p*-values plotted here were not corrected for multiple hypothesis testing. The bar containing the Puf3 motif is indicated. (B) Same as (A), but comparing *H*. *sapiens* and *Danio rerio*. (C) Same as (A), but comparing *H*. *sapiens* and *D*. *melanogaster*.
(PDF)

S4 Fig Scatterplot of Puf motif frequency versus GC content in 3' UTRs by species. Each point was calculated from data from one of the 80 fungi listed in S19 Fig GC content, and motif frequency was calculated from all 3' UTRs, which are defined as the 500 nucleotides downstream of the stop codon.
(PDF)

S5 Fig Conservation of the Puf3 RNA binding domain across fungi. Part (A) is analogous to S2C Fig. Parts (B) and (C) are analogous to S2B and S2D Fig. These results are from the analysis of fungal Puf3 proteins from species in S19 Fig instead of from the eukaryotes listed in S20 Fig. Percent identities and conserved residues can be found in S2 Dataset.
(PDF)

S6 Fig Overlap of Puf3 RNA target sets and enrichment of the Puf3 motif in target RNAs. (A) The intersection of Puf3 target sets from experimental association data in this study and in Gerber et al. [25]. (B) The number of RNAs with a match to the Puf3 motif within or outside of (i.e., in coding sequence or 5’ UTR) the 3' UTR. UTRs were defined using annotations from Nagalakshmi et al. [80].
(PDF)

S7 Fig Conservation of preference for upstream cytosine in Puf3 binding specificity. (A) The fraction of Puf3 motif matches (UGUA[ACU]AUA) that have a −2C residue. *S*. *cerevisiae* Puf3 has a preference for cytosine two nucleotides upstream of its core motif (conventionally called −2C). We tested whether cytosine is enriched at the −2 position by comparing the prevalence of cytosine at motif matches within the conserved Saccharomycotina Puf3 targets to the prevalence at motif matches found within those not defined as conserved. The 5' most motif match for each ortholog was used. Fisher's exact test *p*-values are represented by the color scale and are corrected for multiple hypothesis testing. The preference for the −2C is observed for Puf3 within Saccharomycetaceae and the CTG clade (a lineage of yeast that use CTG to specify the amino acid serine instead of leucine). In contrast, it is not observed for Puf3 in the basal Saccharomycotina species *Yarrowia lipolytica* or for Puf3 in *A*. *oligospora* and *T*. *melanosporum*. We therefore infer that the −2C preference was gained after Puf3 began interacting with RNAs encoding mitochondrial proteins. (B) Summary and sequence logos for analysis of the −2C preference of Puf3. The target groups consist of conserved Saccharomycotina Puf3 targets; all others RNAs are placed in the nontarget group. A sequence logo was made from sequences matching the UGUA[ACU]AUA Puf3 motif and sequences two nucleotides upstream. The odds-ratio and *p*-values from Fisher's exact test for comparing prevalence of the −2C in targets versus nontargets are reported. The column on the right contains four amino acid residues that interact with the −2C in *S*. *cerevisiae* Puf3 crystal structures [49]. Amino acids were extracted from a multiple sequence alignment of Puf3 proteins made using MUSCLE. For *C*. *ablicans* strain WO1, the predicted Puf3 protein was truncated at the C-terminus, so we used a tBLASTn search to identify the corresponding residues. A Puf3 ortholog was not found within the assembled genomic sequence for *S*. *cerevisiae* RM11-1a or *S*. *kudriavzevii*.
(PDF)

S8 Fig Significant motifs identified in orthologs of Saccharomycotina Puf3 targets. Sequence logos of significant motifs identified by REFINE. The left column contains motifs similar to Puf3. The second and third columns contain the Puf-like motifs that provide evidence for recognition by Puf4. The fourth column contains all other significant motifs identified. The *Coprinopsis cinerea* motif resembles the motifs obtained from *S*. *cerevisiae* Puf1 and Puf2 targets (Hogan et al. Puf1 and Puf2 motifs are UAAUAAUUAAU and UAAUAAU[AU], respectively, and Yosefzon et al. Puf2 motif is UAAUnnnUAAU [15,145], raising the possibility that a Puf protein related to Puf1 (and Puf2) could be regulating these RNAs in *Co*. *cinerea* instead of Puf3 or Puf4. The identification of *Co*. *cinerea* motif additionally suggests that post-transcriptional regulation of these RNAs may not be unique to Saccharomycotina and Pezizomycotina fungi.
(PDF)

S9 Fig Distribution of Puf proteins and events in their evolutionary history across fungi. This figure is analogous to S1 Fig but with results from analysis of the fungal Puf proteins. The question mark represents uncertainty in the assignment of an *S*. *kluyveri* Puf as a Puf8 ortholog (Materials and Methods), with the uncertainty stemming from the inference that Puf8 was deleted in an ancestor to *S*. *kluyveri* and that the RNA-contacting amino acids differ from those in other Puf8 proteins. We hypothesize that this additional Puf in *S*. *kluyveri* is the result of a recent Puf3 duplication that has undergone significant divergence. S2 Table contains information about the Puf proteins in each species.
(PDF)

S10 Fig Types and properties of Puf proteins in representative fungi. (A) Puf proteins present in representative fungi. The adjacent cladogram represents the relationships of the Pufs to each other (based on phylogeny in S22 Fig). Red numbers are references to a deletion or duplication event inferred using parsimony (also presented in Fig 8). (B) Protein domain structure of *S*. *cerevisiae* and *N*. *crassa* Puf proteins. A red box indicates a Puf repeat, and a blue box indicates an RNA Recognition Motif (RRM) identified using the SMART annotation [114,115]. Closely related Pufs are grouped together. (C) Pattern of amino acids predicted to contact RNA bases within each Puf repeat. The sequence at the top represents the RNA base preferred to interact with each Puf3 repeat. The amino acids were extracted from a multiple sequence alignment of Puf proteins. The repeats are shown in reverse, starting with the C-terminal repeat on the left. Amino acids that differ from Puf3 are shown in purple. *N*. *crassa* (Nc) Puf1 has a few differences from its orthologs Puf1 and Puf2 in *S*. *cerevisiae* (Sc), so each is shown separately.
(PDF)

S11 Fig Sequence similarity networks for finding Puf motifs. At the top of each panel is a sequence similarity network made from 10mer sequences found to be enriched in the target set compared to the nontarget set (Materials and Methods). The sequence similarity network was used to identify subsets of 10mers that could be grouped together based on a common feature. A node represents a 10mer, and an edge (gray line) is present between nodes that differ by only one nucleotide. 10mers with two or more nucleotides different from all other 10mers are not shown. The remainder of the panel contains a key and summary for groups made from the sequence similarity network. Each panel is formatted the same way. For "# of UTRs matching 10mers", each UTR can only be assigned one 10mer, and the assigned 10mer is the first match found in the iterative search. (A) Network for Puf3 in post-whole-genome-duplication (post-WGD) species. In-group consists of 1,534 *S*. *cerevisiae* Puf3 targets and orthologs in post-WGD species (*n* = 8). The top 48 10mers are represented one or more times in 71.1% of UTRs (1090/1534), compared to 9.7% of other UTRs. (B) Network for Puf4 in post-WGD species. In-group consists of 1,399 *S*. *cerevisiae* Puf4 targets and orthologs in post-WGD species (*n* = 8). The top 66 10mers are represented one or more times in 53.5% of UTRs (748/1399), compared to 7.2% of other UTRs. (C) Network for Puf5 in post-WGD species. In-group consists of 1343 *S*. *cerevisiae* Puf5 targets and orthologs in post-WGD species (*n* = 8). The top 46 10mers are represented one or more times in 48.7% of UTRs (654/1343), compared to 5.6% of other UTRs. (D) Network for Pezizomycotina Puf4. In-group consists of 8,418 orthologs of conserved Saccharomycotina Puf3 targets in Leotiomyceta species (*n* = 42). The top 100 10mers are represented one or more times in 57% of UTRs (4817/8418), compared to 11.2% of other UTRs.
(PDF)

S12 Fig Evidence that Pezizomycotina Puf4 binds its own transcripts. To further explore whether the Puf binding sites found in the ancestral Puf3 targets are used by Puf4, we took advantage of the observation that RNA binding proteins often associate with their own transcript, presumably as a form of autoregulation [15]. For example, 76% of Puf3 (16/21) and 73% of Puf4 (16/22) transcript 3' UTRs in Saccharomycotina species contain a putative binding site for the protein they encode, whereas Puf3 (3/21, 14%) and Puf4 (4/22, 18%) transcript 3' UTRs do not tend to contain a putative binding site for each other's protein (S6 Table). This figure indicates the presence or absence of a Pezizomycotina Puf4 motif in Puf protein transcripts. The Pezizomycotina Puf4 motif from Fig 5B was used to search the 3' UTRs of Puf transcripts in Pezizomycotina species. The FDRs listed below the figure were calculated from conservation scores and comparison to scores derived from permuted motifs (Materials and Methods). Putative Pezizomycotina Puf binding sites are found in the 3' UTRs of transcripts encoding the Puf4 ortholog in a majority of the Pezizomycotina species analyzed (29/44 species, 66%, FDR = 0.08%) but not in transcripts encoding other Puf orthologs (i.e., for Puf1, Puf3, Puf6, Puf8, or Nop9 transcripts, binding sites found in <25% of Pezizomycotina species each with a FDR of >5%).
(PDF)

S13 Fig Linear growth assays of *N*. *crassa* Puf knockouts. Barplots of linear growth rates (left) and relative growth rates (right; relative to wild-type) for *N*. *crassa* wild-type and Puf mutant strains. Error bars represent two times the standard error of the mean. The Puf1 knockout strain used displayed a severe growth defect and a defect in producing conidia. S7 Table lists growth rates and results of *t* tests.
(PDF)

S14 Fig Species enriched with Pezizomycotina Puf4 motifs in the orthologs of Saccharomycotina Puf3 targets. Barplot representing enrichment of Pezizomycotina Puf4 motifs in orthologs of Saccharomycotina Puf3 targets. The *p*-value represents the significance that Pezizomycotina Puf4 motif matches are found in 3' UTRs of Saccharomycotina Puf3 target relative to all other 3' UTRs. The *p*-value was computed using Fisher's exact test and is corrected for multiple hypothesis testing using the Bonferroni method. Values are listed in S1 Dataset.
(PDF)

S15 Fig Comparison of Leotiomyceta Puf4 targets and Saccharomycotina Puf3 targets. (A) Comparison of conserved Saccharomycotina Puf3 and Leotiomyceta Puf4 targets. Each set was broken into three groups based on overlap with each other and whether an ortholog was found (n/a denotes subset without orthologs). The conserved Saccharomycotina Puf3 targets were defined using orthologs of *S*. *cerevisiae* proteins, and the conserved Leotiomyceta Puf4 targets were defined using orthologs of *N*. *crassa* proteins. Leotiomyceta Puf3 targets within each group are reported for comparison and were defined by conservation of Puf3 recognition sequences. The annotations for mitochondrial proteins is based on GO term GO:0005739 for Saccharomycotina and *N*. *crassa* annotations from Keeping et al. [83] for Leotiomyceta. (B) Heatmaps show the prevalence of Puf3 or Pezizomycotina Puf4 motif matches across species (rows) and ortholog sets (columns). Complete motif search results can be found in S8 Dataset.
(PDF)

S16 Fig Puf3 and Puf4 binding sites show no evidence for mutual dependence within ancestral Puf3 targets in Orbiliomycetes. (A) The number of ancestral Puf3 targets that contain a Puf3 or Puf4 binding site. As one of the Pezizomycotina Puf4 motifs can overlap with the Puf3 motif, we removed this Pezizomycotina Puf4 submotif to prevent the overlap (see Table 1 for an additional test that does not remove the Puf4 submotif). Here, Fisher's exact tests were used to test for dependence between the ancestral Puf3 targets bound by Puf3 or Puf4—i.e., whether Puf3 and Puf4 are likely to bind the same subset of ancestral Puf3 targets (overlap more than expected), a different subset (overlap less than expected), or show no relationship. Ancestral Puf3 targets were defined as targets bound by Puf3 prior to the transfer and bound by Puf4 after the transfer (i.e., the intersection of Leotiomyceta Puf4 targets and Saccharomycotina Puf3 targets). (B) Phylogeny for the three Orbiliomycetes species analyzed herein. The tree was extracted from a larger phylogeny inferred using a maximum-likelihood approach from a concatenated alignment of 18 protein sequences. The inferred relationship for these Orbiliomycetes is consistent with previous work [86]. (C) Gain and loss rates for Puf3 and Puf4 binding sites within ancestral Puf3 targets in Orbiliomycetes. We used Bayesian inference to derive gain and loss rates under two models of binding site changes: one "dependence" model in which Puf binding site changes are dependent on the current state of Puf3 and Puf4 sites and an "independence" model in which Puf binding site change is not dependent on the presence of the other's site. Both models have four states and permit single-step transitions between the states. The "dependence" model is unrestricted and has eight free parameters, a gain and loss rate between each state. In the "independence" model, parallel gain or loss rates were restricted to be equal to each other (e.g., the rate of Puf3 gain is the same whether or not a Puf4 site is already present), so this model has four free parameters. Bayesian inference was performed using Multistate in the BayesTraits program and using the conventional Markov chain Monte Carlo (MCMC) method [146]. The rates displayed are the median rate obtained from iterations when the Markov chain was in its stationary phase (uniform priors, 100,000 iterations used as burn-in, subsampled every 1,000 iterations up to iteration 5,000,000). The rates were inferred using the phylogeny in (B) and data from ancestral Puf3 targets, as defined in (A), that have an ortholog in at least two of the three species (*n* = 153). As in (A), the Puf4 submotif that overlaps with Puf3 was removed from the analysis. The results show that the gain and loss rates for Puf3 are largely independent of Puf4 binding site status, and the gains and loss rates for Puf4 are also largely independent of Puf3 binding site status. We compared the models using the Bayes factor, where log Bayes Factor = 2(log[harmonic mean(dependence model)]–log[harmonic mean(independence model)]). The log Bayes Factor is negative, making the simpler "independence" model the favored model.
(PDF)

S17 Fig Change in Puf-RNA interactions revealed from experimental association data. (A) Cumulative distributions of Puf3 immunopurification (IP) enrichment in *S*. *cerevisiae* for different sets of RNAs based on whether the *S*. *cerevisiae* 3' UTR has a Puf3 motif match and whether it is a conserved Saccharomycotina Puf3 target. This analysis compares data from all microarray features (black, *n* = 6,991), RNAs with motif matches (blue, *n* = 1,011), and conserved RNA targets with a motif match (red, *n* = 227) or without a motif match (green, *n* = 44). IP enrichment data are from Gerber et al. [25]. (B) Same as (A), except using conserved Saccharomycotina Puf4 targets, Puf4 IP data, and the Saccharomycotina Puf4 motif. This analysis compares data from all microarray features (black, *n* = 6,834), RNAs with motif matches (blue, *n* = 698), and conserved RNA targets with a motif match (red, *n* = 46) or without a motif match (green, *n* = 81). (C) Same as (A), except using conserved Saccharomycotina Puf5 targets, Puf5 IP data, and the Saccharomycotina Puf5 motif. This analysis compares data from all microarray features (black, *n* = 7,044), RNAs with motif matches (blue, *n* = 437), and conserved RNA targets with a motif match (red, *n* = 14) or without a motif match (green, *n* = 25).
(PDF)

S18 Fig Novel targets of *S*. *cerevisiae* identified through conservation analysis. (A) Heatmap displaying presence of Puf3 motif matches in the 3' UTRs of conserved Saccharomycotina Puf3 targets. The columns of the heatmap are ordered by the conservation score (highest on the left). *S*. *cerevisiae* Puf3 targets as defined by Gerber et al. [25] are noted with green boxes, and RNAs whose encoded protein products are annotated to the mitochondrion are noted with magenta boxes (GO term GO:0005739). (B) Enrichment of RNAs from Puf3 IP experiments. Kernel density plots showing all RNAs (black), RNAs whose protein is annotated as mitochondrion (magenta, GO term GO:0005739), RNAs that are conserved targets of Puf3 and known targets of *S*. *cerevisiae* Puf3 (green), and conserved targets of Puf3 that were not called *S*. *cerevisiae* Puf3 targets (orange). Immunopurification data are from Gerber et al. [25]. (C) Same as (B), except using RNA abundance data from a *puf3*Δ and wild-type *S*. *cerevisiae* profiled using glycerol as a carbon source. Data are from Gerber et al. [25]. (D) Same as (B), except using RNA enrichment data obtained in a comparison of isolated mitochondria to total RNA. Data are from Saint-Georges et al. [44]. (E) Same as (B), except using data from a change in mitochondrion-associated RNA enrichment resulting from *puf3*Δ. Data are from Saint-Georges et al. [44].
(PDF)

S19 Fig Phylogenetic model for changes in Pufs and their interactions with RNA in fungi. Same as Fig 8 but including all species names.
(PDF)

S20 Fig Phylogeny for eukaryotes with species names. This phylogeny includes species names for reference to Fig 2. See Fig 2 legend and Materials and Methods for more information about the phylogeny.
(PDF)

S21 Fig Parsimony-based models for Puf3 and Puf4 binding to the same RNAs. Each panel (A)–(C) presents a comparison of two models using different possible phylogenies for the placement of Orbiliomycetes and Pezizomycetes. Left: Models that Puf3 gained interaction with the RNAs related to Saccharomycotina Puf3 targets in an ancestor to both the Saccharomycotina and Pezizomycotina lineages. Right: Models that Puf3 gained interaction with these RNAs through two independent series of events. For both sets of models, we propose that the Puf3 binding site sequences observed in *T*. *melanosporum* have been gained recently within Pezizomycetes history as this is the most parsimonious explanation (due to the difference of *T*. *melanosporum* from the other two Pezizomycetes species) and considering that the enrichment of Puf3 sites in *T*. *melanosporum* (~20%) is modest. (A) The phylogeny and model on the left are the same as shown in Fig 7B, and are reproduced here for comparison. This phylogeny places Orbiliomycetes as diverging the earliest in Pezizomycotina, followed by Pezizomycetes. If the gain or loss of a target set is considered a single "event", then this model evokes four events in each model to explain the data. (B) Models for the phylogeny in which Pezizomycetes diverged the earliest in Pezizomycotina, followed by Orbiliomycetes. The parallel or convergent evolution model on the right becomes more parsimonious in this phylogeny but is equally parsimonious to both models in (A). (C) Models for the phylogeny in which both Orbiliomycetes and Pezizomycetes diverged from the rest of Pezizomycotina and then diverged from each other. The parallel/convergent evolution model on the right becomes more parsimonious in this phylogeny but is equally parsimonious to both models in (A).
(PDF)

S22 Fig Relationship of *S*. *cerevisiae* and *N*. *crassa* Puf proteins. Puf protein sequences were aligned using MUSCLE (default settings in Geneious), and the resulting multiple alignment was used to build a maximum likelihood tree using PhyML implemented through Geneious (WAG [Whelan And Goldman] substitution model, 8 substitution rate categories, best of NNI and SPR search, 100 bootstraps). Nodes with less than 75% bootstrap support were collapsed. Alignment and newick-formatted tree can be found in S3 Dataset.
(PDF)

S23 Fig Summary and comparison of functionally related groups of proteins encoded by conserved Puf RNA targets. "Saccharomycotina Puf3" refers to the set of conserved Puf3 targets found among Saccharomycotina species, and "Leotiomyceta Puf3" and "Leotiomyceta Puf4" refer to the set of conserved Puf3 and Puf4 targets, respectively, found among Leotiomyceta species. The color in each box represents the Bonferroni-corrected *p*-value from Fisher's exact test, comparing enrichment among the target set to the fraction found among all other ortholog sets. To compare these distantly related fungi, we used annotations from *S*. *cerevisiae* and *N*. *crassa* and included manual assignments in several cases (see S8 Table for details).
(PDF)

S24 Fig Conserved targets of Puf4 and Puf5 and inferring timing of changes. (A) Heatmaps displaying whether respective consensus sequences are found in 3' UTRs of conserved targets of Saccharomycotina Puf4 (top left) or Puf5 (top right). For comparison, an equal number of randomly selected ortholog sets are displayed in the heatmaps at the bottom. The rows represent species data, and the columns represent ortholog sets. The columns of each heatmap are ordered by the conservation score (highest on the left) calculated from Saccharomycotina species data (Materials and Methods). Dark gray bars below each heatmap denote ortholog sets that fall within the respective group as defined by the legend in the middle. Complete motif search results can be found in S8 Dataset. (B) Presence of Puf sequence motif matches in 3' UTRs of histone mRNAs. For Saccharomycotina species, we searched for sequences matching the Saccharomycotina Puf4 or Puf5 motifs; for all other species, we searched for matches to the Pezizomycotina Puf4 motifs, under a model that Pezizomycotina Puf4 motifs represent the ancestral and conserved binding specificity of Puf4. Enrichment of sequences matching each Puf motif within the indicated lineages and for each type of histone was tested, and a conservation score for the real Puf motifs was calculated from comparison to searches with permuted versions of the motifs (Materials and Methods). (Transcripts encoding for the same histone protein are collapsed into one set.) The result of statistical testing is indicated for each lineage. Motif search results and histone 3' UTR sequences can be found in S10 Dataset.
(PDF)

S25 Fig Timing of changes in Puf RNA targets and transcription factor targets. Phylogenetic model for events in the history of Puf protein targets and related transcription factor targets. The timing of changes for transcription factors were inferred from existing literature data. The transcription factor studies that this analysis is based on are cited in S13 Text, which also provides a discussion of these changes.
(PDF)

S1 Table Pufs identified and classified in eukaryotes. (XLSX)

S2 Table Pufs identified and classified in fungi. (XLSX)

S3 Table Sources of genome sequences retrieved for eukaryote species represented within InParanoid. (TXT)

S4 Table Sources of genome and protein sequences retrieved for fungi species. (TXT)

S5 Table Summary of results of mitochondrial groups enriched in different conserved Puf target sets. (TXT)

S6 Table Presence or absence of matches to different Puf motifs in transcripts of Puf proteins. (TXT)

S7 Table Summary of *N*. *crassa* knockout growth experiments. (TXT)

S8 Table Information about group annotation and results of significance testing for types of proteins encoded by conserved Puf targets (for S23 Fig). (TXT)

S9 Table *N*. *crassa* and *S*. *cerevisiae* Puf3 IP data, SAM (Significance Analysis of Microarrays) output, and Puf3 motif matches. (TXT)

S10 Table *N*. *crassa* gene expression data comparing knockouts to wild-type. (TXT)

S11 Table Summary of statistical tests for motifs identified by REFINE in orthologs of Saccharomycotina Puf3 targets. (TXT)

S12 Table Analogous but distinct changes in the evolution of transcription factors and RNA binding proteins. The conservation and changes in Puf-RNA evolution draw several parallels to observations made within the extensively studied evolution of transcription factors and their targets. In the table, we summarize the analogous changes and cite examples from the transcription factor literature [11,64,123,147–159] for comparison.
(PPTX)

S1 Text Delineating the evolutionary history of Puf proteins. (DOCX)

S2 Text Orthologs of Puf3 proteins share a conserved RNA binding interface. (DOCX)

S3 Text Characterization of the binding specificity of *N*. *crassa* Puf3. (DOCX)

S4 Text Analysis of conserved binding sites identifies novel targets of *S*. *cerevisiae* Puf3, Puf4, and Puf5. (DOCX)

S5 Text Predicting which Pezizomycotina Puf proteins bind UGUA-based motifs. (DOCX)

S6 Text The diversification of Puf4 and its targets in Pezizomycotina. (DOCX)

S7 Text Saccharomycotina Puf4 targets diverged after the Puf4 and Puf5 duplication. (DOCX)

S8 Text Histone mRNAs are ancient targets of Puf4 and Puf5. (DOCX)

S9 Text Evidence that Puf4 and Puf5 maintained a small number of ancestral targets after the Puf4 duplication. (DOCX)

S10 Text Puf proteins linked to diverse functions in different species. (DOCX)

S11 Text Puf3 and Puf4 target evolution in Orbiliomycetes consistent with independent selective advantages. (DOCX)

S12 Text Finer scale changes in binding site sequences of conserved Puf targets. (DOCX)

S13 Text Changes at the post-transcriptional level compared to changes in transcriptional regulation. (DOCX)

S14 Text Models for the history of Puf3 and Puf4 interacting with a common set of more than 150 mRNAs. (DOCX)

S15 Text Conservation of RNA-interacting residues after Puf4 duplication suggest possible changes in shape of Puf domain as the origin of binding specificity divergence. (DOCX)

S16 Text Inferring the history of acquisition, loss, and changes in the regulatory specificity of Puf proteins in fungal evolution. (DOCX)

S17 Text Dating the Puf4 and Puf5 duplication. (DOCX)

## Abbreviations

ETC: electron transport chain
FDR: false discovery rate
GO: gene ontology
IP: immunopurification
MCMC: Markov chain Monte Carlo
NNI: Nearest Neighbor Interchange
PDB: Protein Data Bank
Puf: Pumilio–Fem-3-binding factor
RRM: RNA Recognition Motif
SAM: Significance Analysis of Microarrays
SPR: Subtree Pruning and Regrafting
TAP-tag: tandem affinity purification tag
TCA: tricarboxylic acid
UTR: untranslated region
WAG: Whelan and Goldman
