# Supplementary figures and images for: Evolutionary Conservation and Diversification of Puf RNA Binding Proteins and Their mRNA Targets
Source: PLoS Biol. 2015 Nov 20;13(11):e1002307. doi: 10.1371/journal.pbio.1002307 (PMC4654594; doi:10.1371/journal.pbio.1002307)

# Ortholog Classification

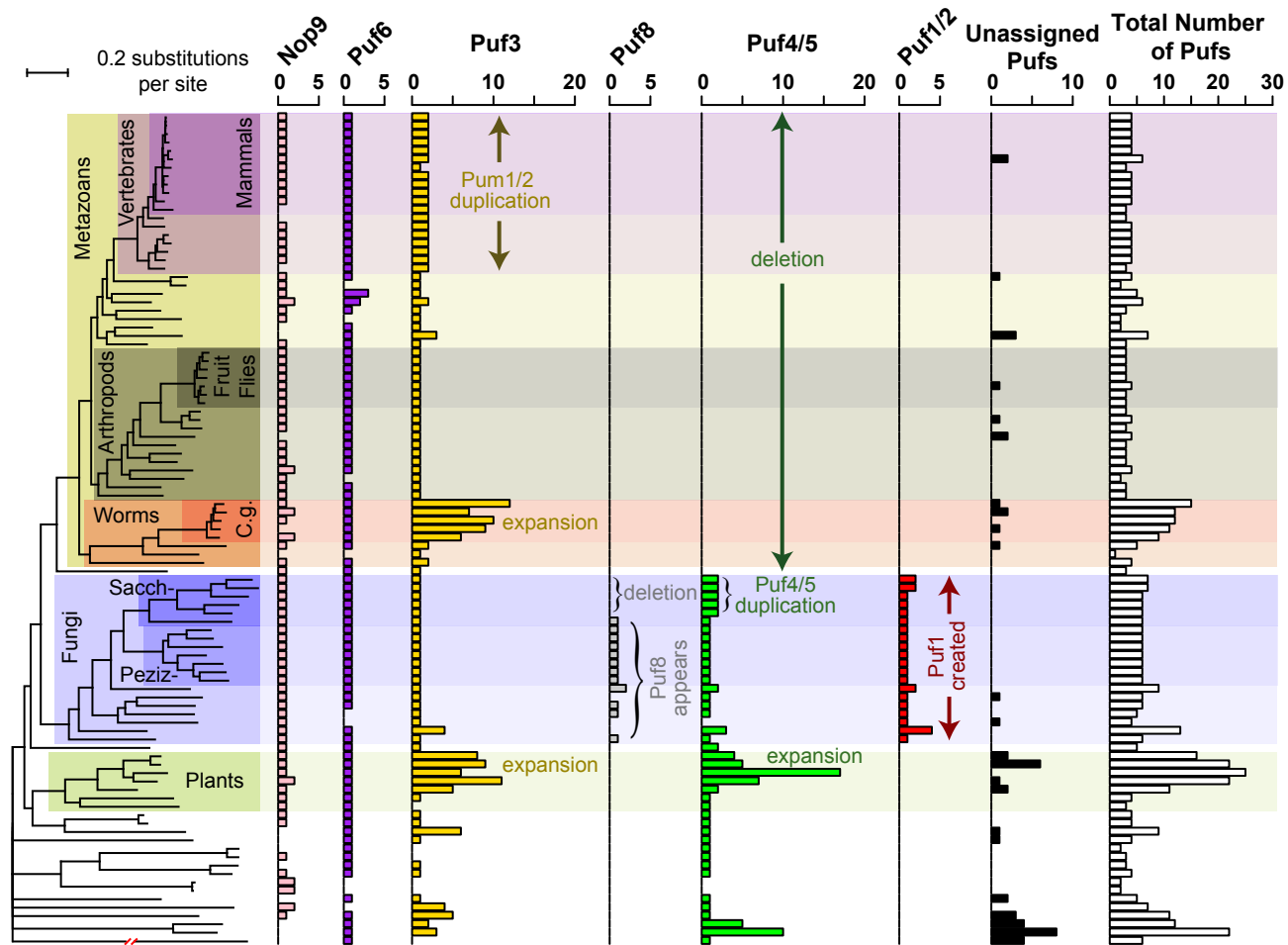

Supp. Figure 1

Supplement: S1 Fig — Barplots display the number and types of Puf proteins identified for each eukaryote species. Events in Puf protein history are noted if supported by the number of Puf proteins or an absence in three or more species. "Sacch-" refers to Saccharomycotina fungi, "Peziz-" to Pezizomycotina fungi, and "C.g." to the Caenorhabditis genus of worms. The break noted in red removed 0.5 from the branch to G. lamblia. The full list of species represented is shown in S20 Fig. S1 Table contains information about the Puf proteins in each species. (PDF) [file pbio.1002307.s011.pdf]

**A**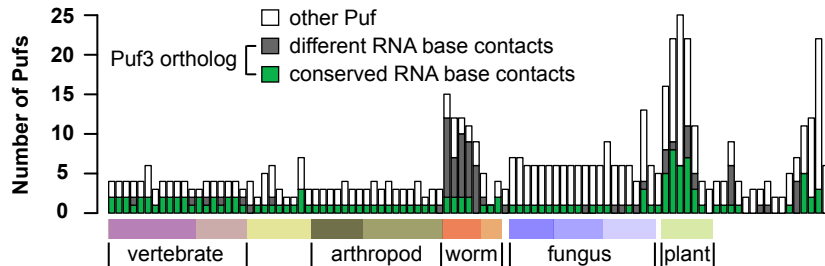**B**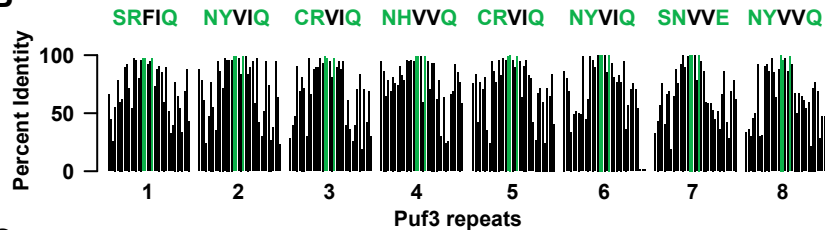**C**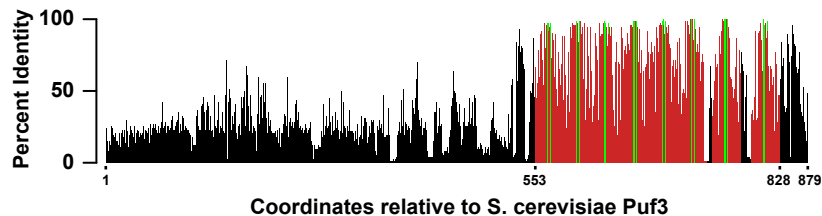**D**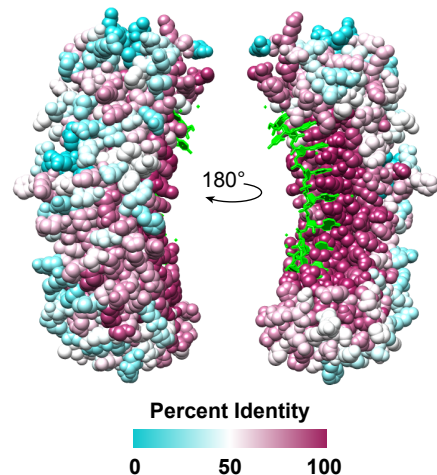

Supplement: S2 Fig — (A) Cataloging Puf genes in each of 99 different eukaryotic genomes. The number of Puf3 orthologs with perfectly conserved RNA-base-contacting amino acids is indicated in green, the number of all other Puf3 orthologs is indicated in dark gray, and the number of other Puf proteins is indicated in white. Species are ordered based on the phylogeny in Fig 2. Colored boxes below the x-axis also correspond to clades as labeled in Fig 2. S1 Table contains information about the Puf proteins in each species. (B) Percent identity of each amino acid in each Puf repeat, in Puf3 orthologs. Amino acids in green denote RNA-base-contacting residues. Percent identity was calculated based on multiple sequence alignment of orthologs defined as reciprocal best BLAST hits to S. cerevisiae Puf3 (n = 74). This approach avoids paralogs of Puf3 in a species that may have diverged following a duplication event. We calculated the percent identity of each set of residues that aligned to a S. cerevisiae Puf3 residue. Percent identities and conserved residues can be found in S2 Dataset. (C) Same as (B) but with percent identities plotted for the full-length S. cerevisiae Puf3. Residues in the Puf repeats are noted in red, and RNA-contacting residues are in green. (D) Amino acid percent identity displayed on structure of S. cerevisiae Puf3's RNA-binding domain. Structure displayed represents two different views of a space-filling model from PDB entry 3k49 [49]. The RNA molecule with sequence CCUGUAAAUA is indicated in green. The structures are oriented with the 5' end of the RNA and the C-terminus of the protein at the top as in Fig 1. (PDF) [file pbio.1002307.s012.pdf]

**Puf3 motif**

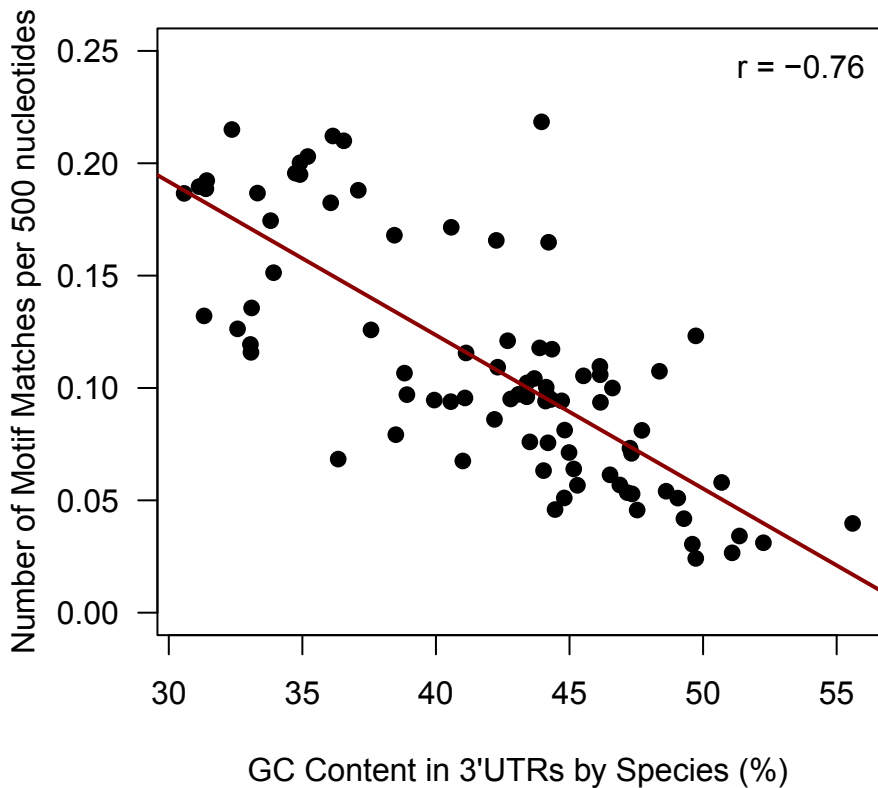

**Pezizomycotina Puf4 motif**

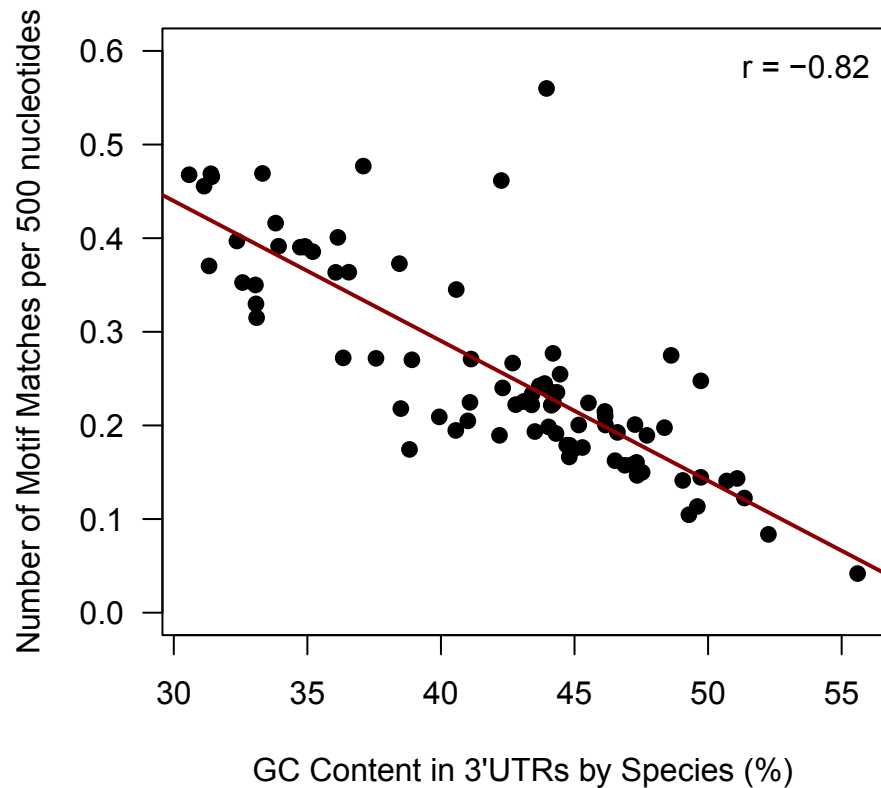

Supplement: S4 Fig — Each point was calculated from data from one of the 80 fungi listed in S19 Fig GC content, and motif frequency was calculated from all 3' UTRs, which are defined as the 500 nucleotides downstream of the stop codon. (PDF) [file pbio.1002307.s014.pdf]

**A**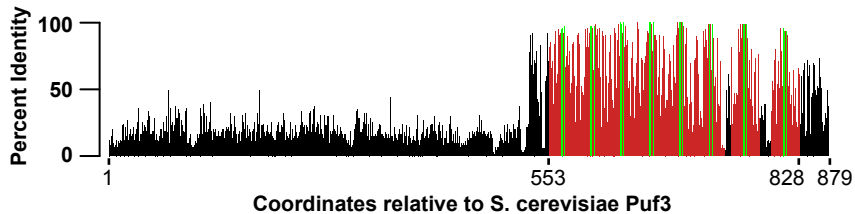**B**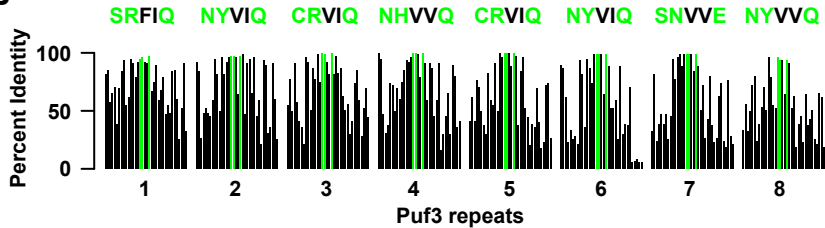**C**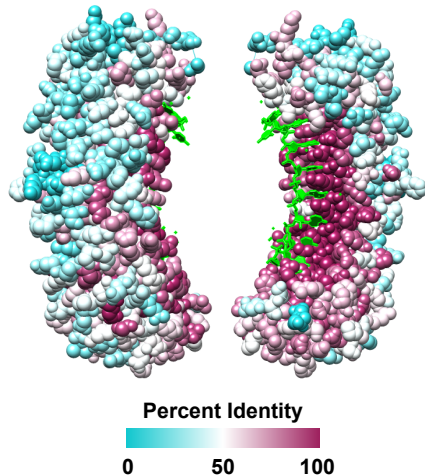

Supplement: S5 Fig — Part (A) is analogous to S2C Fig. Parts (B) and (C) are analogous to S2B and S2D Fig. These results are from the analysis of fungal Puf3 proteins from species in S19 Fig instead of from the eukaryotes listed in S20 Fig. Percent identities and conserved residues can be found in S2 Dataset. (PDF) [file pbio.1002307.s015.pdf]

## Puf3 Motifs

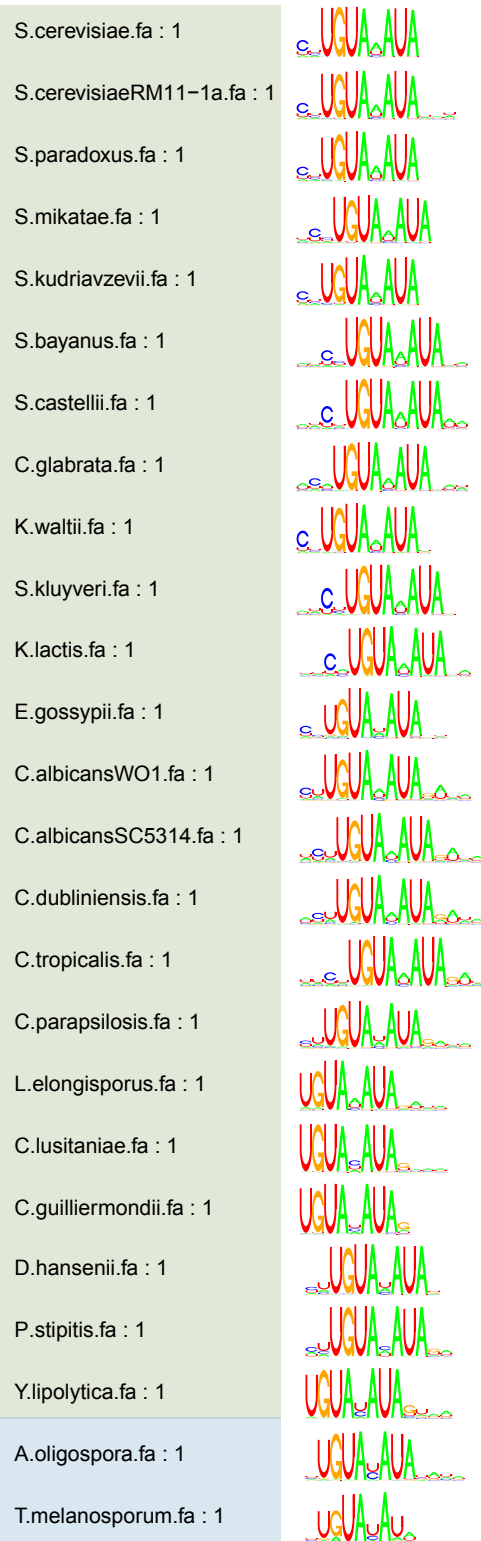

## Puf4 Motifs

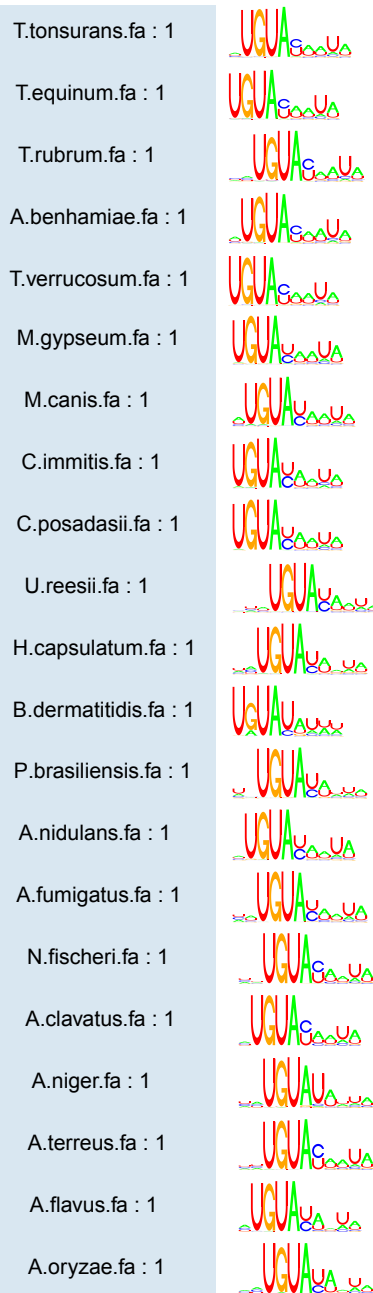

## Other Motifs

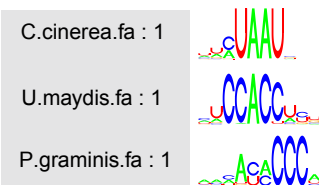

Supplement: S8 Fig — Sequence logos of significant motifs identified by REFINE. The left column contains motifs similar to Puf3. The second and third columns contain the Puf-like motifs that provide evidence for recognition by Puf4. The fourth column contains all other significant motifs identified. The Coprinopsis cinerea motif resembles the motifs obtained from S. cerevisiae Puf1 and Puf2 targets (Hogan et al. Puf1 and Puf2 motifs are UAAUAAUUAAU and UAAUAAU[AU], respectively, and Yosefzon et al. Puf2 motif is UAAUnnnUAAU [15,145], raising the possibility that a Puf protein related to Puf1 (and Puf2) could be regulating these RNAs in Co. cinerea instead of Puf3 or Puf4. The identification of Co. cinerea motif additionally suggests that post-transcriptional regulation of these RNAs may not be unique to Saccharomycotina and Pezizomycotina fungi. (PDF) [file pbio.1002307.s018.pdf]

Ortholog Classification

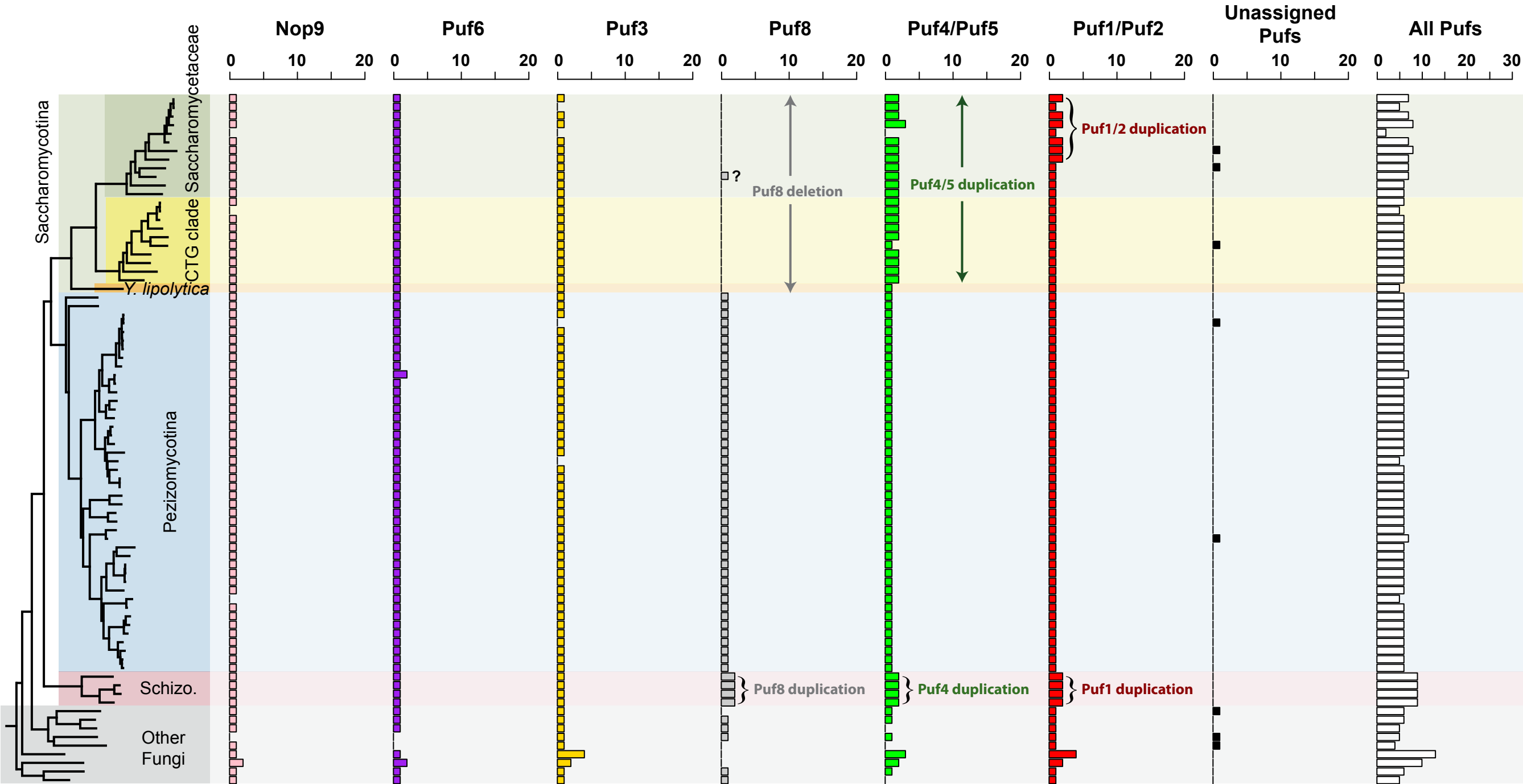

Supplement: S9 Fig — This figure is analogous to S1 Fig but with results from analysis of the fungal Puf proteins. The question mark represents uncertainty in the assignment of an S. kluyveri Puf as a Puf8 ortholog (Materials and Methods), with the uncertainty stemming from the inference that Puf8 was deleted in an ancestor to S. kluyveri and that the RNA-contacting amino acids differ from those in other Puf8 proteins. We hypothesize that this additional Puf in S. kluyveri is the result of a recent Puf3 duplication that has undergone significant divergence. S2 Table contains information about the Puf proteins in each species. (PDF) [file pbio.1002307.s019.pdf]

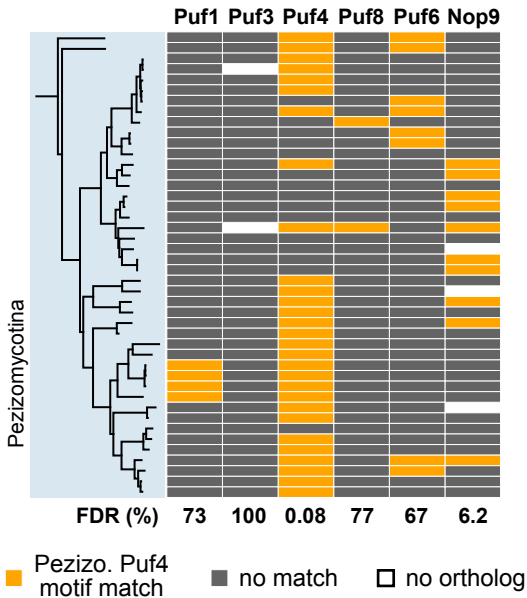

**Supp. Figure 12**

Supplement: S12 Fig — To further explore whether the Puf binding sites found in the ancestral Puf3 targets are used by Puf4, we took advantage of the observation that RNA binding proteins often associate with their own transcript, presumably as a form of autoregulation [15]. For example, 76% of Puf3 (16/21) and 73% of Puf4 (16/22) transcript 3' UTRs in Saccharomycotina species contain a putative binding site for the protein they encode, whereas Puf3 (3/21, 14%) and Puf4 (4/22, 18%) transcript 3' UTRs do not tend to contain a putative binding site for each other's protein (S6 Table). This figure indicates the presence or absence of a Pezizomycotina Puf4 motif in Puf protein transcripts. The Pezizomycotina Puf4 motif from Fig 5B was used to search the 3' UTRs of Puf transcripts in Pezizomycotina species. The FDRs listed below the figure were calculated from conservation scores and comparison to scores derived from permuted motifs (Materials and Methods). Putative Pezizomycotina Puf binding sites are found in the 3' UTRs of transcripts encoding the Puf4 ortholog in a majority of the Pezizomycotina species analyzed (29/44 species, 66%, FDR = 0.08%) but not in transcripts encoding other Puf orthologs (i.e., for Puf1, Puf3, Puf6, Puf8, or Nop9 transcripts, binding sites found in <25% of Pezizomycotina species each with a FDR of >5%). (PDF) [file pbio.1002307.s022.pdf]

**A**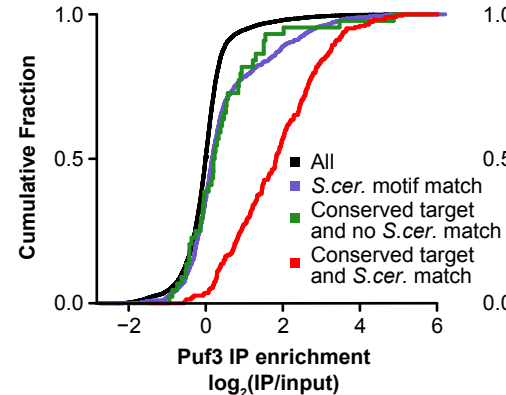**B**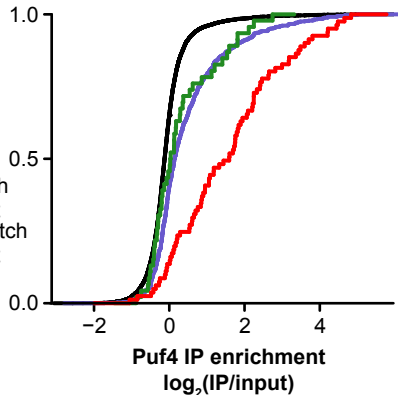**C**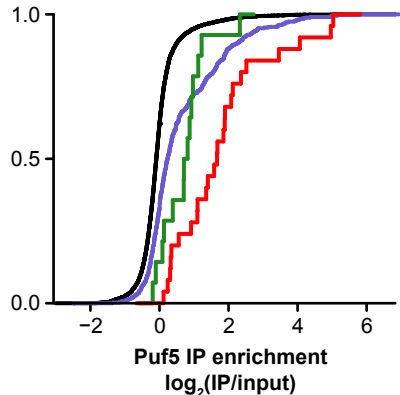

Supplement: S17 Fig — (A) Cumulative distributions of Puf3 immunopurification (IP) enrichment in S. cerevisiae for different sets of RNAs based on whether the S. cerevisiae 3' UTR has a Puf3 motif match and whether it is a conserved Saccharomycotina Puf3 target. This analysis compares data from all microarray features (black, n = 6,991), RNAs with motif matches (blue, n = 1,011), and conserved RNA targets with a motif match (red, n = 227) or without a motif match (green, n = 44). IP enrichment data are from Gerber et al. [25]. (B) Same as (A), except using conserved Saccharomycotina Puf4 targets, Puf4 IP data, and the Saccharomycotina Puf4 motif. This analysis compares data from all microarray features (black, n = 6,834), RNAs with motif matches (blue, n = 698), and conserved RNA targets with a motif match (red, n = 46) or without a motif match (green, n = 81). (C) Same as (A), except using conserved Saccharomycotina Puf5 targets, Puf5 IP data, and the Saccharomycotina Puf5 motif. This analysis compares data from all microarray features (black, n = 7,044), RNAs with motif matches (blue, n = 437), and conserved RNA targets with a motif match (red, n = 14) or without a motif match (green, n = 25). (PDF) [file pbio.1002307.s027.pdf]

**A****Conserved Targets of Saccharomycotina Puf3 (n = 276)**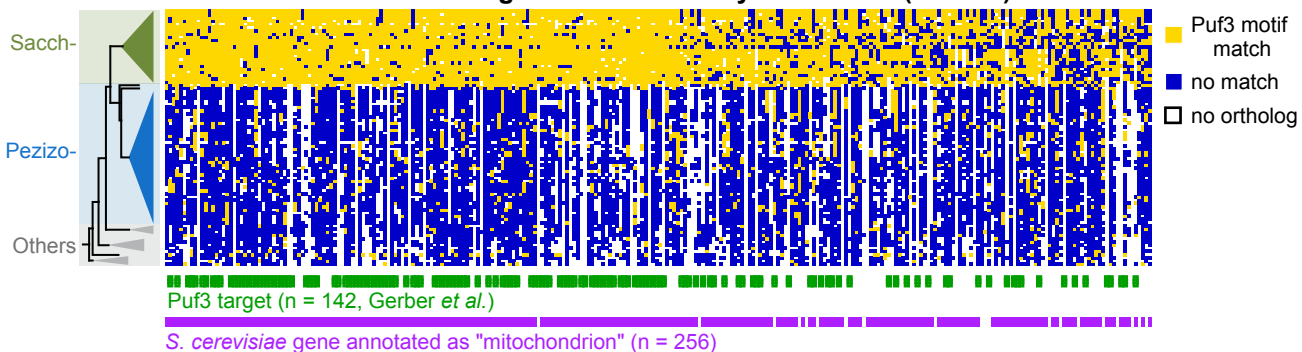**B**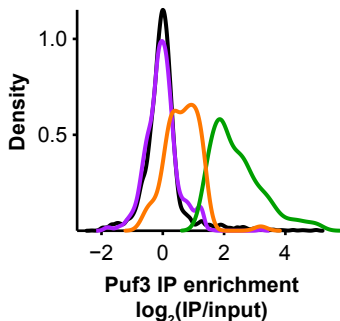**C**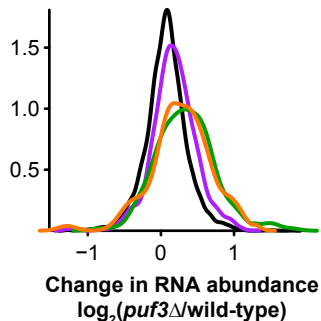**D**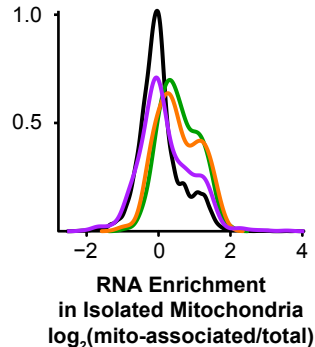**E**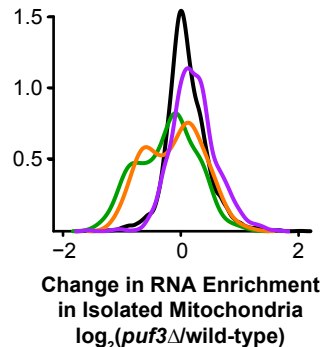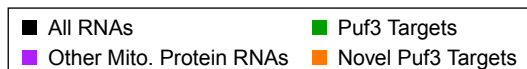

Supplement: S18 Fig — (A) Heatmap displaying presence of Puf3 motif matches in the 3' UTRs of conserved Saccharomycotina Puf3 targets. The columns of the heatmap are ordered by the conservation score (highest on the left). S. cerevisiae Puf3 targets as defined by Gerber et al. [25] are noted with green boxes, and RNAs whose encoded protein products are annotated to the mitochondrion are noted with magenta boxes (GO term GO:0005739). (B) Enrichment of RNAs from Puf3 IP experiments. Kernel density plots showing all RNAs (black), RNAs whose protein is annotated as mitochondrion (magenta, GO term GO:0005739), RNAs that are conserved targets of Puf3 and known targets of S. cerevisiae Puf3 (green), and conserved targets of Puf3 that were not called S. cerevisiae Puf3 targets (orange). Immunopurification data are from Gerber et al. [25]. (C) Same as (B), except using RNA abundance data from a puf3Δ and wild-type S. cerevisiae profiled using glycerol as a carbon source. Data are from Gerber et al. [25]. (D) Same as (B), except using RNA enrichment data obtained in a comparison of isolated mitochondria to total RNA. Data are from Saint-Georges et al. [44]. (E) Same as (B), except using data from a change in mitochondrion-associated RNA enrichment resulting from puf3Δ. Data are from Saint-Georges et al. [44]. (PDF) [file pbio.1002307.s028.pdf]

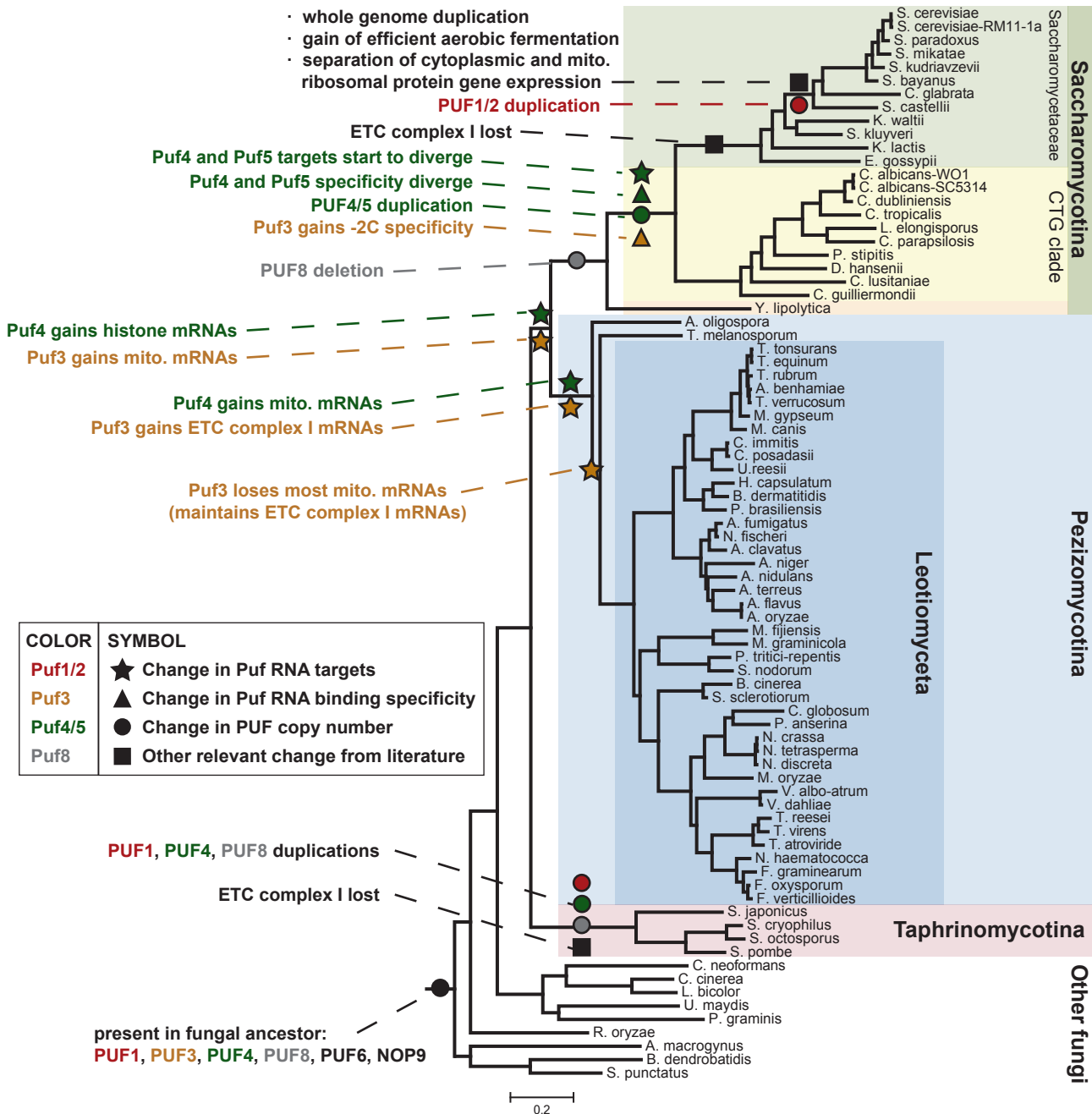

Supplement: S19 Fig — Same as Fig 8 but including all species names. (PDF) [file pbio.1002307.s029.pdf]

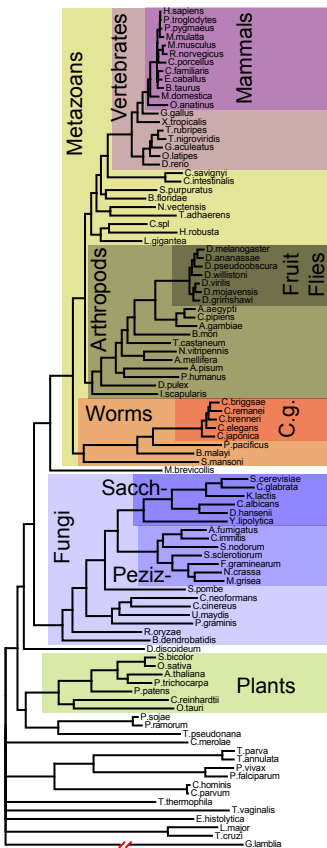

Sacch- = Saccharomycotina fungi  
 Peziz- = Pezizomycotina fungi  
 C.g. = Caenorhabditis genus of worms

Supplement: S20 Fig — This phylogeny includes species names for reference to Fig 2. See Fig 2 legend and Materials and Methods for more information about the phylogeny. (PDF) [file pbio.1002307.s030.pdf]

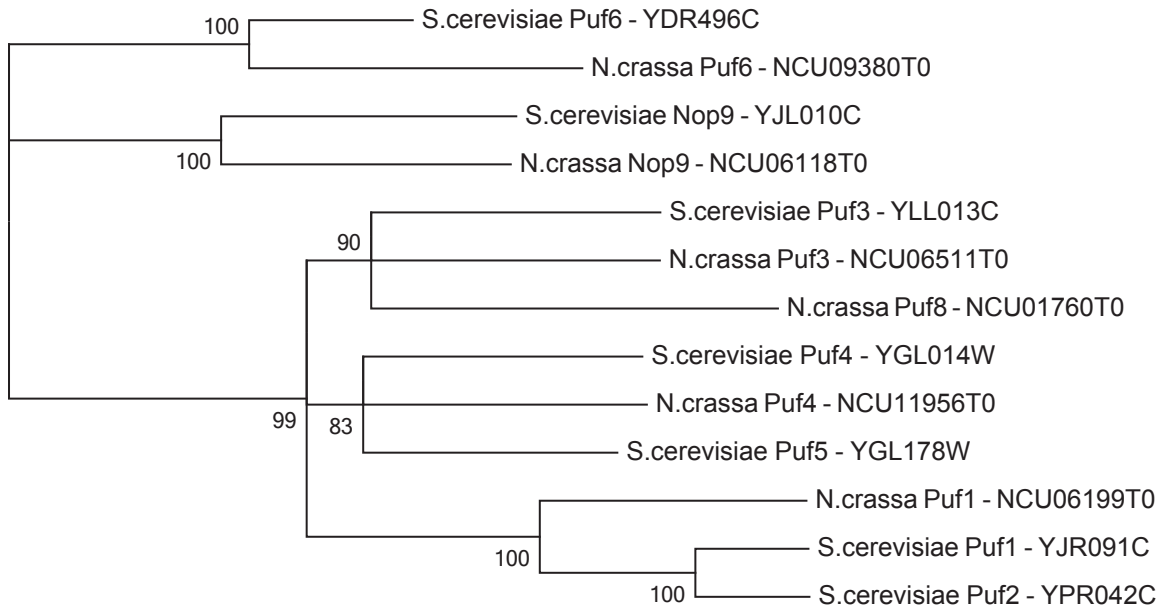

0.5

Supplement: S22 Fig — Puf protein sequences were aligned using MUSCLE (default settings in Geneious), and the resulting multiple alignment was used to build a maximum likelihood tree using PhyML implemented through Geneious (WAG [Whelan And Goldman] substitution model, 8 substitution rate categories, best of NNI and SPR search, 100 bootstraps). Nodes with less than 75% bootstrap support were collapsed. Alignment and newick-formatted tree can be found in S3 Dataset. (PDF) [file pbio.1002307.s032.pdf]

# Fraction That Are Targets

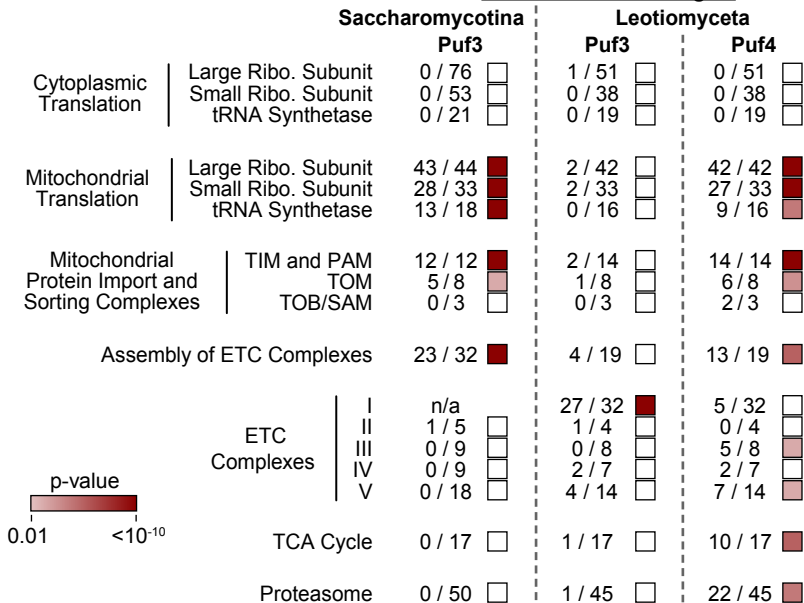

Supplement: S23 Fig — "Saccharomycotina Puf3" refers to the set of conserved Puf3 targets found among Saccharomycotina species, and "Leotiomyceta Puf3" and "Leotiomyceta Puf4" refer to the set of conserved Puf3 and Puf4 targets, respectively, found among Leotiomyceta species. The color in each box represents the Bonferroni-corrected p-value from Fisher's exact test, comparing enrichment among the target set to the fraction found among all other ortholog sets. To compare these distantly related fungi, we used annotations from S. cerevisiae and N. crassa and included manual assignments in several cases (see S8 Table for details). (PDF) [file pbio.1002307.s033.pdf]

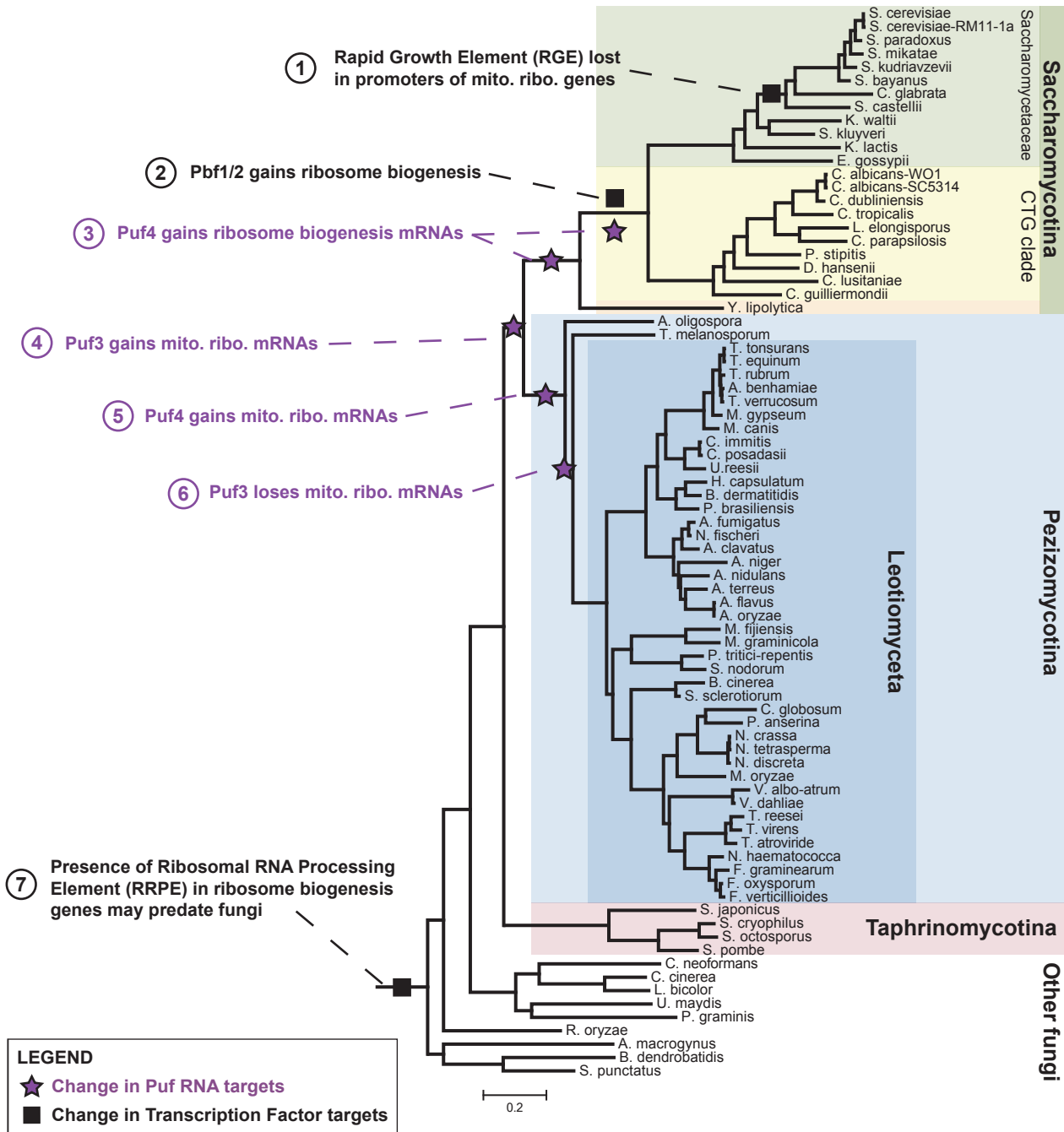

Supplement: S25 Fig — Phylogenetic model for events in the history of Puf protein targets and related transcription factor targets. The timing of changes for transcription factors were inferred from existing literature data. The transcription factor studies that this analysis is based on are cited in S13 Text, which also provides a discussion of these changes. (PDF) [file pbio.1002307.s035.pdf]
