# Supplementary material for: Evolutionary Conservation and Diversification of Puf RNA Binding Proteins and Their mRNA Targets
Source: PLoS Biol. 2015 Nov 20;13(11):e1002307. doi: 10.1371/journal.pbio.1002307 (PMC4654594; doi:10.1371/journal.pbio.1002307)
Supplement: S3 Fig — (A) Histogram representing the distribution of hypergeometric p-values for the Puf3 motif and all 1,119 permutations in the comparison of Homo sapiens and Mus musculus. The p-values plotted here were not corrected for multiple hypothesis testing. The bar containing the Puf3 motif is indicated. (B) Same as (A), but comparing H. sapiens and Danio rerio. (C) Same as (A), but comparing H. sapiens and D. melanogaster. (PDF) [file pbio.1002307.s013.pdf]

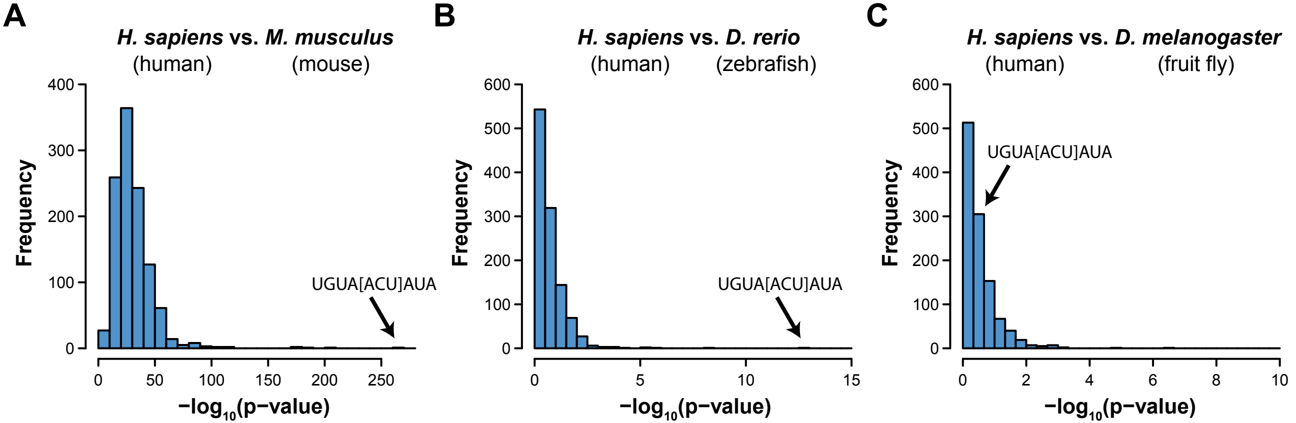

Supp. Figure 3
