# Supplementary material for: Evolutionary Conservation and Diversification of Puf RNA Binding Proteins and Their mRNA Targets
Source: PLoS Biol. 2015 Nov 20;13(11):e1002307. doi: 10.1371/journal.pbio.1002307 (PMC4654594; doi:10.1371/journal.pbio.1002307)
Supplement: S6 Fig — (A) The intersection of Puf3 target sets from experimental association data in this study and in Gerber et al. [25]. (B) The number of RNAs with a match to the Puf3 motif within or outside of (i.e., in coding sequence or 5’ UTR) the 3' UTR. UTRs were defined using annotations from Nagalakshmi et al. [80]. (PDF) [file pbio.1002307.s016.pdf]

A

	Overlap of Puf3 Target Sets (from IP data)		
	N. crassa (this study)*	*S. cerevisiae* (this study)	*S. cerevisiae* (Gerber *et al.*)
N. crassa **(this study)***	(250)	83	48
S. cerevisiae **(this study)**	83	(392)	194
S. cerevisiae **(Gerber *et al.*)**	48	194	(220)

B

		**# with Motif Match**	
	# RNAs	Non-3' UTR	3' UTR
N. crassa **Puf3 Targets (this study)***	250	47 (19%)	83 (33%)
S. cerevisiae **Puf3 Targets (this study)**	392	99 (25%)	241 (61%)
S. cerevisiae **Puf3 Targets (Gerber *et al.*)**	220	50 (23%)	159 (73%)
**All RNAs**	6228	518 (8%)	710 (11%)

* - "targets" are RNAs bound by *N. crassa* Puf3 when ectopically expressed in *S. cerevisiae*

Supp. Figure 6
