# Supplementary material for: Evolutionary Conservation and Diversification of Puf RNA Binding Proteins and Their mRNA Targets
Source: PLoS Biol. 2015 Nov 20;13(11):e1002307. doi: 10.1371/journal.pbio.1002307 (PMC4654594; doi:10.1371/journal.pbio.1002307)
Supplement: S7 Fig — (A) The fraction of Puf3 motif matches (UGUA[ACU]AUA) that have a −2C residue. S. cerevisiae Puf3 has a preference for cytosine two nucleotides upstream of its core motif (conventionally called −2C). We tested whether cytosine is enriched at the −2 position by comparing the prevalence of cytosine at motif matches within the conserved Saccharomycotina Puf3 targets to the prevalence at motif matches found within those not defined as conserved. The 5' most motif match for each ortholog was used. Fisher's exact test p-values are represented by the color scale and are corrected for multiple hypothesis testing. The preference for the −2C is observed for Puf3 within Saccharomycetaceae and the CTG clade (a lineage of yeast that use CTG to specify the amino acid serine instead of leucine). In contrast, it is not observed for Puf3 in the basal Saccharomycotina species Yarrowia lipolytica or for Puf3 in A. oligospora and T. melanosporum. We therefore infer that the −2C preference was gained after Puf3 began interacting with RNAs encoding mitochondrial proteins. (B) Summary and sequence logos for analysis of the −2C preference of Puf3. The target groups consist of conserved Saccharomycotina Puf3 targets; all others RNAs are placed in the nontarget group. A sequence logo was made from sequences matching the UGUA[ACU]AUA Puf3 motif and sequences two nucleotides upstream. The odds-ratio and p-values from Fisher's exact test for comparing prevalence of the −2C in targets versus nontargets are reported. The column on the right contains four amino acid residues that interact with the −2C in S. cerevisiae Puf3 crystal structures [49]. Amino acids were extracted from a multiple sequence alignment of Puf3 proteins made using MUSCLE. For C. ablicans strain WO1, the predicted Puf3 protein was truncated at the C-terminus, so we used a tBLASTn search to identify the corresponding residues. A Puf3 ortholog was not found within the assembled genomic sequence for S. cerevisiae RM11-1a or S. ku [file pbio.1002307.s017.pdf]

A

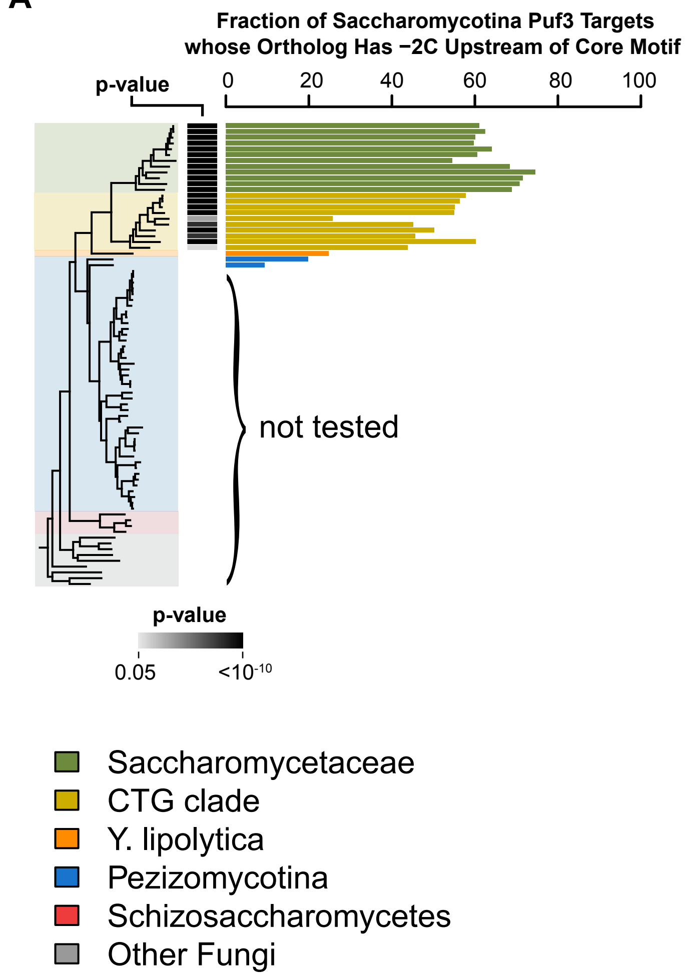

B

Species	Target Group	Non-target Group	-2C enrichment Odds-ratio	p-value*	Residues Aligned to *S. cerevisiae* -2C Contacts#
S.cerevisiae	141/231 (61%)	135/801 (17%)	7.7	8e−36	K,LAS
S.cerevisiaeRM11.1a	140/224 (62%)	129/730 (18%)	7.7	1.8e−34	?
S.paradoxus	128/213 (60%)	121/723 (17%)	7.5	9.5e−32	K,LAS
S.mikatae	116/194 (60%)	96/612 (16%)	8	8.1e−30	K,LAS
S.kudriavzevii	109/170 (64%)	101/511 (20%)	7.2	2.4e−24	?
S.bayanus	129/213 (61%)	110/741 (15%)	8.8	2.1e−36	K,LAS
C.glabrata	121/222 (55%)	80/477 (17%)	5.9	3e−22	R,LAS
S.castellii	132/193 (68%)	78/476 (16%)	11	1.4e−36	R,LAS
K.waltii	135/181 (75%)	69/285 (24%)	9.1	7.4e−26	R,LAS
S.kluyveri	161/225 (72%)	103/489 (21%)	9.4	1.1e−36	R,LAS
K.lactis	160/226 (71%)	108/559 (19%)	10	9.1e−41	R,LAS
E.gossypii	113/164 (69%)	64/339 (19%)	9.5	2.1e−26	R,LAS
C.albicansWO1	130/225 (58%)	60/461 (13%)	9.1	3.7e−32	K,LAS
C.albicansSC5314	129/229 (56%)	64/486 (13%)	8.5	7.3e−31	K,LAS
C.dubliniensis	127/230 (55%)	61/467 (13%)	8.2	1.9e−29	K,LAS
C.tropicalis	126/229 (55%)	64/455 (14%)	7.4	5.1e−27	K,LAS
L.elongisporus	51/198 (26%)	54/483 (11%)	2.8	1e−04	K,LAS
C.parapsilosis	88/195 (45%)	52/288 (18%)	3.7	6.4e−09	G,--S or G,KKS
D.hansenii	105/230 (46%)	106/480 (22%)	3	7.7e−09	K,LAS
P.stipitis	122/243 (50%)	68/447 (15%)	5.6	1.2e−20	K,LAS
C.guilliermondii	68/155 (44%)	39/156 (25%)	2.3	0.013	K,LAS
C.lusitaniae	118/196 (60%)	41/179 (23%)	5.1	6.5e−12	K,LAS
Y.lipolytica	46/186 (25%)	91/522 (17%)	1.6	0.99	K,LAS
A.oligospora	29/146 (20%)	48/312 (15%)	1.4	1	K,LNA
T.melanosporum	4/43 (9.3%)	31/137 (23%)	0.35	1	R,INA

* - Bonferroni corrected
\# - *S. cerevisiae* -2C contacting residues are 819, 864-866

Supp Figure 7
