# Supplementary material for: Evolutionary Conservation and Diversification of Puf RNA Binding Proteins and Their mRNA Targets
Source: PLoS Biol. 2015 Nov 20;13(11):e1002307. doi: 10.1371/journal.pbio.1002307 (PMC4654594; doi:10.1371/journal.pbio.1002307)
Supplement: S10 Fig — (A) Puf proteins present in representative fungi. The adjacent cladogram represents the relationships of the Pufs to each other (based on phylogeny in S22 Fig). Red numbers are references to a deletion or duplication event inferred using parsimony (also presented in Fig 8). (B) Protein domain structure of S. cerevisiae and N. crassa Puf proteins. A red box indicates a Puf repeat, and a blue box indicates an RNA Recognition Motif (RRM) identified using the SMART annotation [114,115]. Closely related Pufs are grouped together. (C) Pattern of amino acids predicted to contact RNA bases within each Puf repeat. The sequence at the top represents the RNA base preferred to interact with each Puf3 repeat. The amino acids were extracted from a multiple sequence alignment of Puf proteins. The repeats are shown in reverse, starting with the C-terminal repeat on the left. Amino acids that differ from Puf3 are shown in purple. N. crassa (Nc) Puf1 has a few differences from its orthologs Puf1 and Puf2 in S. cerevisiae (Sc), so each is shown separately. (PDF) [file pbio.1002307.s020.pdf]

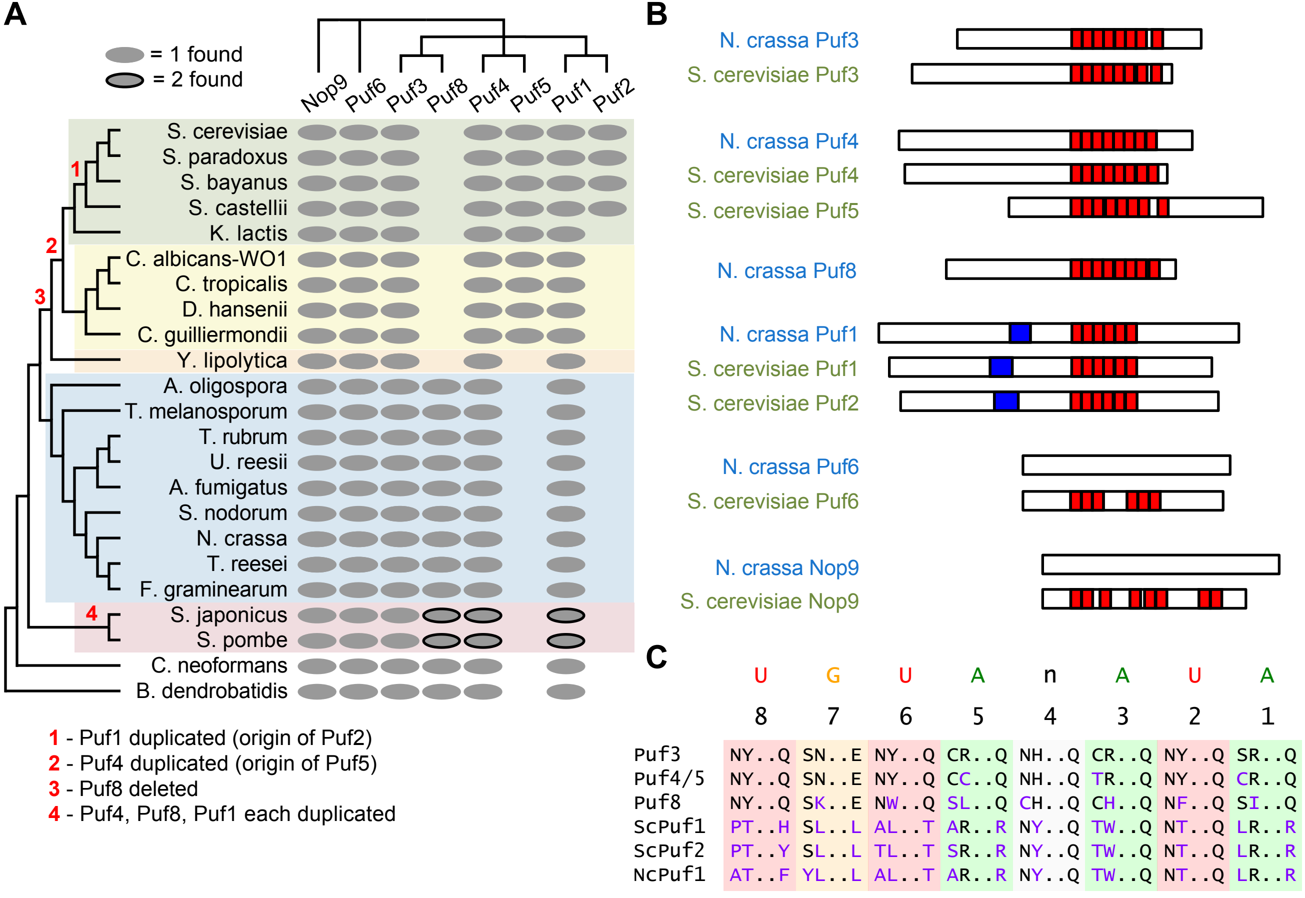

Supp. Figure 10
