# Supplementary material for: Evolutionary Conservation and Diversification of Puf RNA Binding Proteins and Their mRNA Targets
Source: PLoS Biol. 2015 Nov 20;13(11):e1002307. doi: 10.1371/journal.pbio.1002307 (PMC4654594; doi:10.1371/journal.pbio.1002307)
Supplement: S13 Fig — Barplots of linear growth rates (left) and relative growth rates (right; relative to wild-type) for N. crassa wild-type and Puf mutant strains. Error bars represent two times the standard error of the mean. The Puf1 knockout strain used displayed a severe growth defect and a defect in producing conidia. S7 Table lists growth rates and results of t tests. (PDF) [file pbio.1002307.s023.pdf]

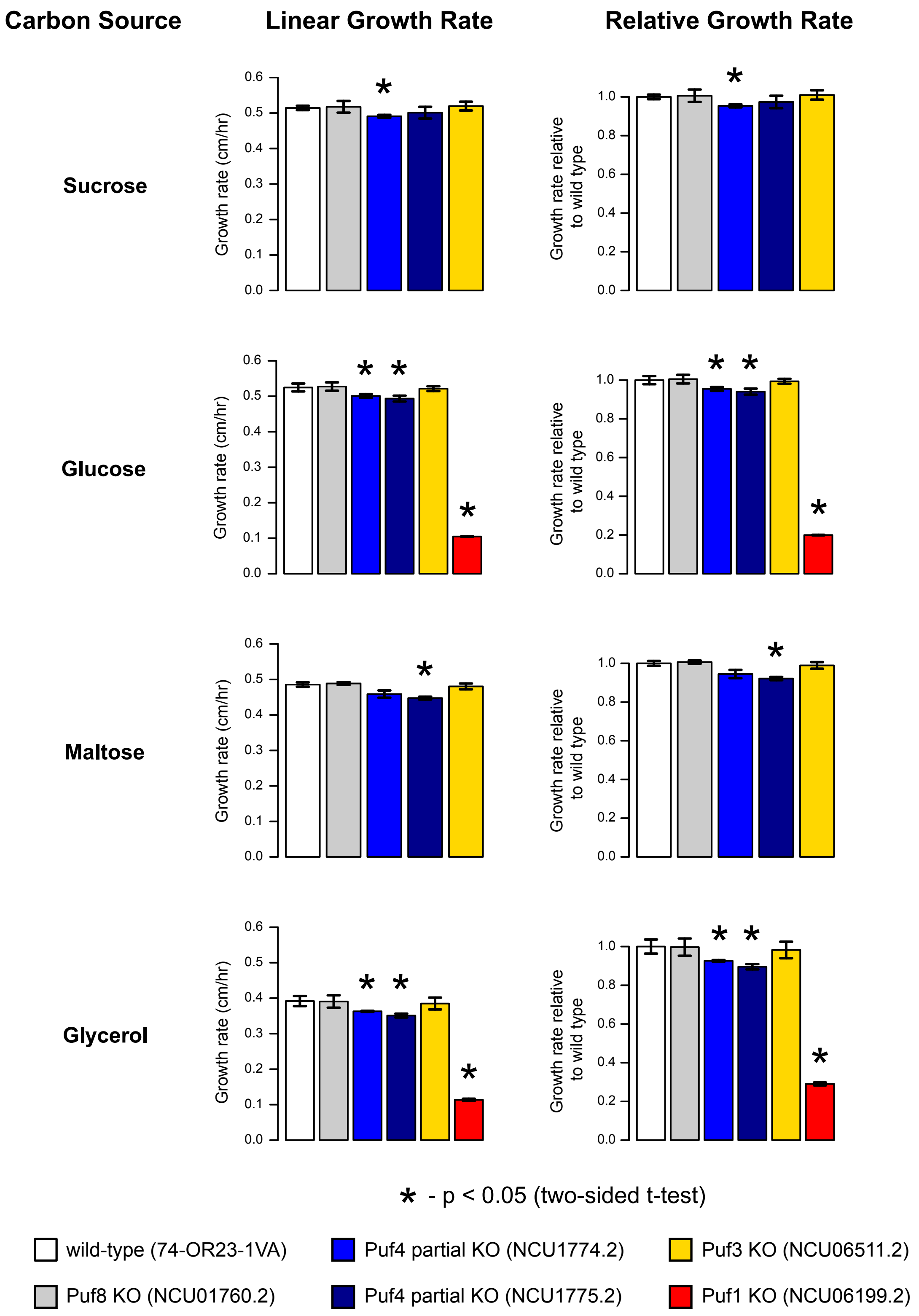

Supp. Figure 13
