# Supplementary material for: Evolutionary Conservation and Diversification of Puf RNA Binding Proteins and Their mRNA Targets
Source: PLoS Biol. 2015 Nov 20;13(11):e1002307. doi: 10.1371/journal.pbio.1002307 (PMC4654594; doi:10.1371/journal.pbio.1002307)
Supplement: S14 Fig — Barplot representing enrichment of Pezizomycotina Puf4 motifs in orthologs of Saccharomycotina Puf3 targets. The p-value represents the significance that Pezizomycotina Puf4 motif matches are found in 3' UTRs of Saccharomycotina Puf3 target relative to all other 3' UTRs. The p-value was computed using Fisher's exact test and is corrected for multiple hypothesis testing using the Bonferroni method. Values are listed in S1 Dataset. (PDF) [file pbio.1002307.s024.pdf]

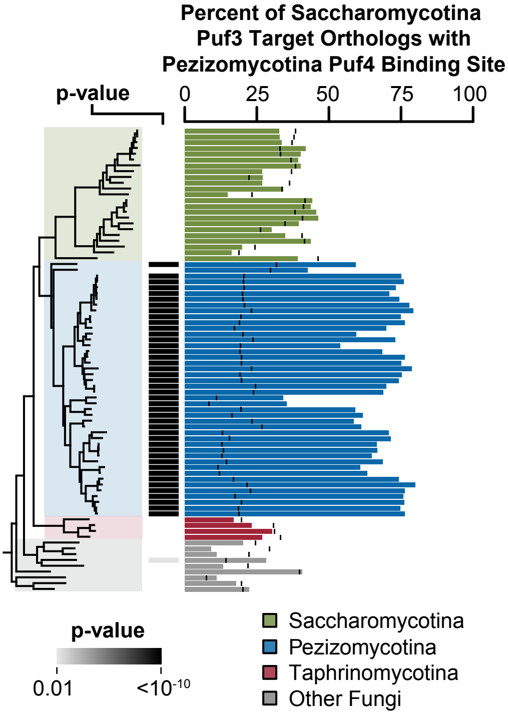

Supp. Figure 14
