# Supplementary material for: Evolutionary Conservation and Diversification of Puf RNA Binding Proteins and Their mRNA Targets
Source: PLoS Biol. 2015 Nov 20;13(11):e1002307. doi: 10.1371/journal.pbio.1002307 (PMC4654594; doi:10.1371/journal.pbio.1002307)
Supplement: S15 Fig — (A) Comparison of conserved Saccharomycotina Puf3 and Leotiomyceta Puf4 targets. Each set was broken into three groups based on overlap with each other and whether an ortholog was found (n/a denotes subset without orthologs). The conserved Saccharomycotina Puf3 targets were defined using orthologs of S. cerevisiae proteins, and the conserved Leotiomyceta Puf4 targets were defined using orthologs of N. crassa proteins. Leotiomyceta Puf3 targets within each group are reported for comparison and were defined by conservation of Puf3 recognition sequences. The annotations for mitochondrial proteins is based on GO term GO:0005739 for Saccharomycotina and N. crassa annotations from Keeping et al. [83] for Leotiomyceta. (B) Heatmaps show the prevalence of Puf3 or Pezizomycotina Puf4 motif matches across species (rows) and ortholog sets (columns). Complete motif search results can be found in S8 Dataset. (PDF) [file pbio.1002307.s025.pdf]

A

Subsets of Saccharomycotina Puf3 (S) or Leotiomyceta Puf4 (L) Targets

Targets divided into subsets based on overlap with each other.

	S-I	S-II	S-III	S-total	L-I	L-II	L-III	L-total
Sacch. Puf3 Targets	+	+	+	+	+	-	n/a	+/-
Leotio. Puf4 Targets	+	-	n/a	+/-	+	+	+	+
Puf3 Targets (Gerber *et al.*)	95	9	43	147	95	8	n/a	103
Leotio. Puf3 Targets	24	2	n/a	26	24	25	23	72
Mitochondrial Protein	162	27	67	256	150	69	78	297
Size	164	38	74	276	164	243	198	605

B

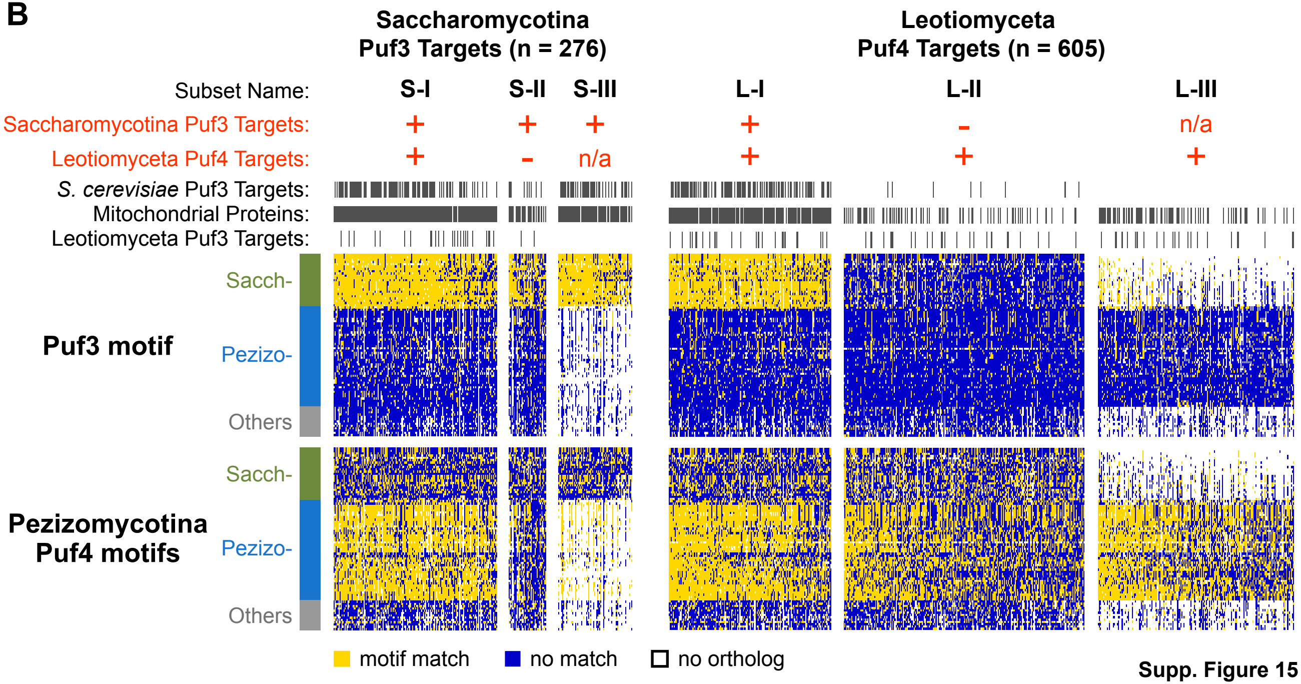

Supp. Figure 15
