# Supplementary material for: Evolutionary Conservation and Diversification of Puf RNA Binding Proteins and Their mRNA Targets
Source: PLoS Biol. 2015 Nov 20;13(11):e1002307. doi: 10.1371/journal.pbio.1002307 (PMC4654594; doi:10.1371/journal.pbio.1002307)
Supplement: S16 Fig — (A) The number of ancestral Puf3 targets that contain a Puf3 or Puf4 binding site. As one of the Pezizomycotina Puf4 motifs can overlap with the Puf3 motif, we removed this Pezizomycotina Puf4 submotif to prevent the overlap (see Table 1 for an additional test that does not remove the Puf4 submotif). Here, Fisher's exact tests were used to test for dependence between the ancestral Puf3 targets bound by Puf3 or Puf4—i.e., whether Puf3 and Puf4 are likely to bind the same subset of ancestral Puf3 targets (overlap more than expected), a different subset (overlap less than expected), or show no relationship. Ancestral Puf3 targets were defined as targets bound by Puf3 prior to the transfer and bound by Puf4 after the transfer (i.e., the intersection of Leotiomyceta Puf4 targets and Saccharomycotina Puf3 targets). (B) Phylogeny for the three Orbiliomycetes species analyzed herein. The tree was extracted from a larger phylogeny inferred using a maximum-likelihood approach from a concatenated alignment of 18 protein sequences. The inferred relationship for these Orbiliomycetes is consistent with previous work [86]. (C) Gain and loss rates for Puf3 and Puf4 binding sites within ancestral Puf3 targets in Orbiliomycetes. We used Bayesian inference to derive gain and loss rates under two models of binding site changes: one "dependence" model in which Puf binding site changes are dependent on the current state of Puf3 and Puf4 sites and an "independence" model in which Puf binding site change is not dependent on the presence of the other's site. Both models have four states and permit single-step transitions between the states. The "dependence" model is unrestricted and has eight free parameters, a gain and loss rate between each state. In the "independence" model, parallel gain or loss rates were restricted to be equal to each other (e.g., the rate of Puf3 gain is the same whether or not a Puf4 site is already present), so this model has four free parameters. Bayesian inferenc [file pbio.1002307.s026.pdf]

A

Number of RNAs with motif matches in "ancestral Puf3 targets"

	Puf3 and Puf4*	Puf3 only	Puf4 only*	neither	odds-ratio (95% CI)	p-value
D. stenobrocha	38	71	10	15	0.80 (0.32 - 2.01)	0.65
D. haptotyla	46	63	16	24	1.09 (0.51 - 2.32)	0.85
A. oligospora	49	74	9	19	1.39 (0.56 - 3.58)	0.52

*** The Puf4 submotif UGUA[ACU].[AU].UA was removed from this analysis to prevent overlap with the Puf3 motif.**

B

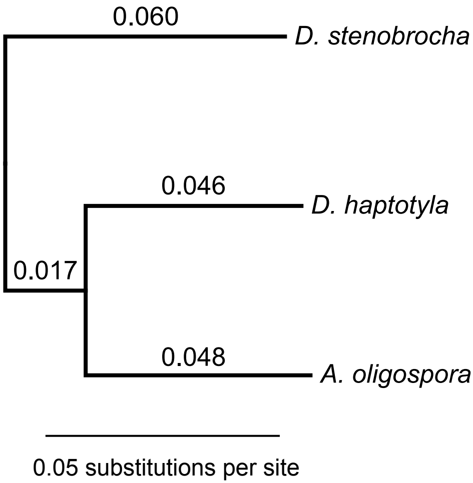

C

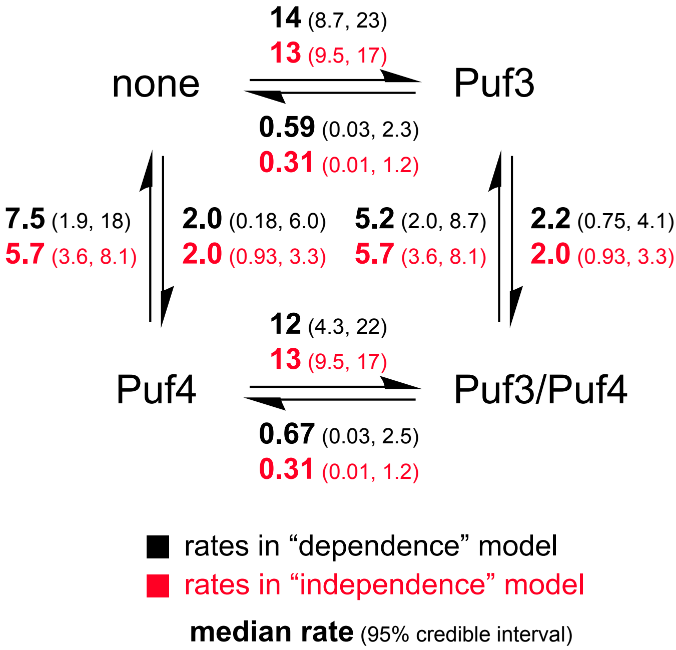

Comparing Model Likelihoods:

log[harmonic mean(**dependence model**)] = -242.2

log[harmonic mean(**independence model**)] = -234.5

log Bayes Factor = 2 * (-242.2 --234.5) = -15

log Bayes Factor < 0, favoring **independence model**

Supp. Figure 16
