# Supplementary material for: Evolutionary Conservation and Diversification of Puf RNA Binding Proteins and Their mRNA Targets
Source: PLoS Biol. 2015 Nov 20;13(11):e1002307. doi: 10.1371/journal.pbio.1002307 (PMC4654594; doi:10.1371/journal.pbio.1002307)

Supp. Figure 21

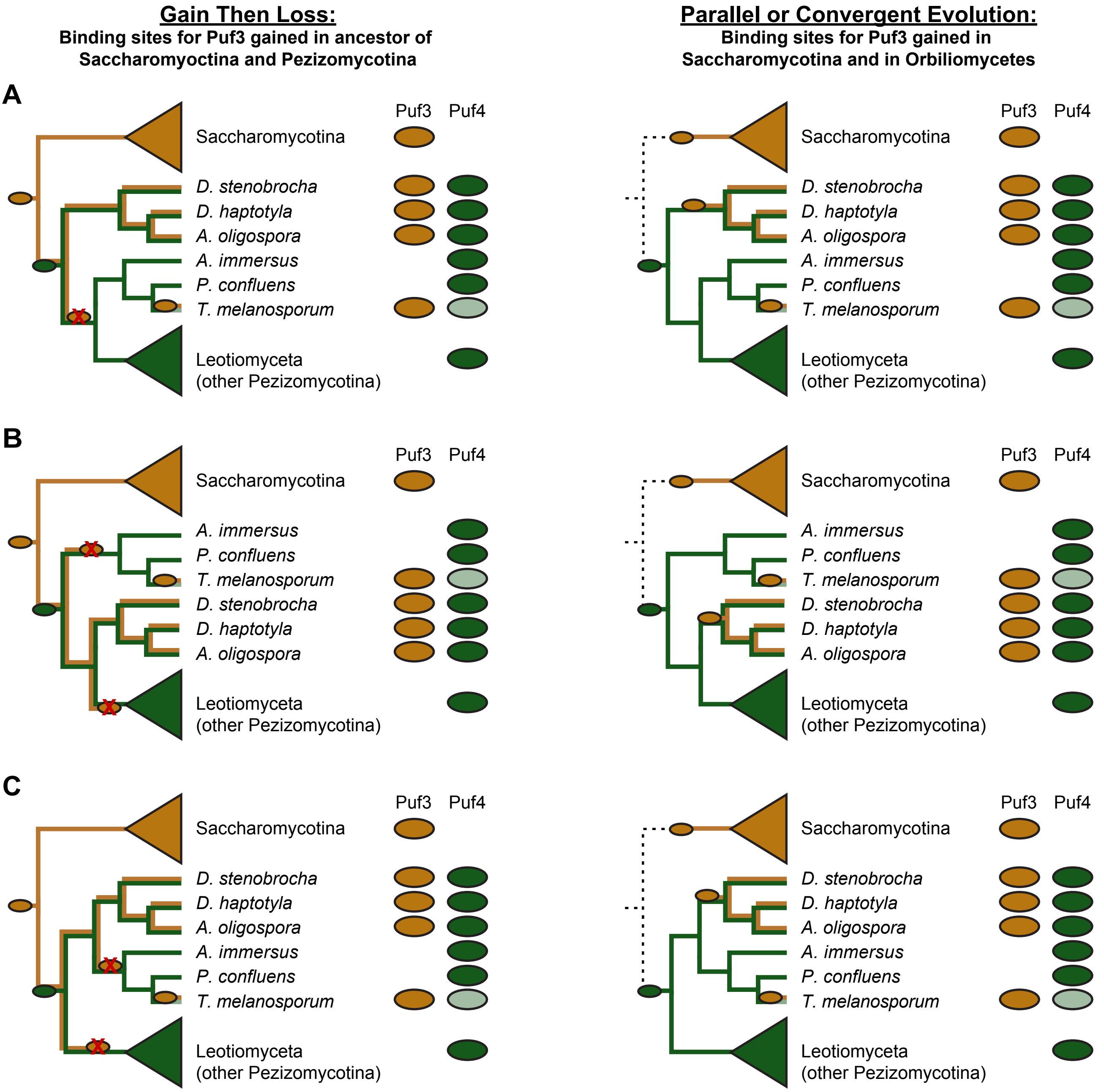

Supplement: S21 Fig — Each panel (A)–(C) presents a comparison of two models using different possible phylogenies for the placement of Orbiliomycetes and Pezizomycetes. Left: Models that Puf3 gained interaction with the RNAs related to Saccharomycotina Puf3 targets in an ancestor to both the Saccharomycotina and Pezizomycotina lineages. Right: Models that Puf3 gained interaction with these RNAs through two independent series of events. For both sets of models, we propose that the Puf3 binding site sequences observed in T. melanosporum have been gained recently within Pezizomycetes history as this is the most parsimonious explanation (due to the difference of T. melanosporum from the other two Pezizomycetes species) and considering that the enrichment of Puf3 sites in T. melanosporum (~20%) is modest. (A) The phylogeny and model on the left are the same as shown in Fig 7B, and are reproduced here for comparison. This phylogeny places Orbiliomycetes as diverging the earliest in Pezizomycotina, followed by Pezizomycetes. If the gain or loss of a target set is considered a single "event", then this model evokes four events in each model to explain the data. (B) Models for the phylogeny in which Pezizomycetes diverged the earliest in Pezizomycotina, followed by Orbiliomycetes. The parallel or convergent evolution model on the right becomes more parsimonious in this phylogeny but is equally parsimonious to both models in (A). (C) Models for the phylogeny in which both Orbiliomycetes and Pezizomycetes diverged from the rest of Pezizomycotina and then diverged from each other. The parallel/convergent evolution model on the right becomes more parsimonious in this phylogeny but is equally parsimonious to both models in (A). (PDF) [file pbio.1002307.s031.pdf]
