# Supplementary material for: Evolutionary Conservation and Diversification of Puf RNA Binding Proteins and Their mRNA Targets
Source: PLoS Biol. 2015 Nov 20;13(11):e1002307. doi: 10.1371/journal.pbio.1002307 (PMC4654594; doi:10.1371/journal.pbio.1002307)
Supplement: S24 Fig — (A) Heatmaps displaying whether respective consensus sequences are found in 3' UTRs of conserved targets of Saccharomycotina Puf4 (top left) or Puf5 (top right). For comparison, an equal number of randomly selected ortholog sets are displayed in the heatmaps at the bottom. The rows represent species data, and the columns represent ortholog sets. The columns of each heatmap are ordered by the conservation score (highest on the left) calculated from Saccharomycotina species data (Materials and Methods). Dark gray bars below each heatmap denote ortholog sets that fall within the respective group as defined by the legend in the middle. Complete motif search results can be found in S8 Dataset. (B) Presence of Puf sequence motif matches in 3' UTRs of histone mRNAs. For Saccharomycotina species, we searched for sequences matching the Saccharomycotina Puf4 or Puf5 motifs; for all other species, we searched for matches to the Pezizomycotina Puf4 motifs, under a model that Pezizomycotina Puf4 motifs represent the ancestral and conserved binding specificity of Puf4. Enrichment of sequences matching each Puf motif within the indicated lineages and for each type of histone was tested, and a conservation score for the real Puf motifs was calculated from comparison to searches with permuted versions of the motifs (Materials and Methods). (Transcripts encoding for the same histone protein are collapsed into one set.) The result of statistical testing is indicated for each lineage. Motif search results and histone 3' UTR sequences can be found in S10 Dataset. (PDF) [file pbio.1002307.s034.pdf]

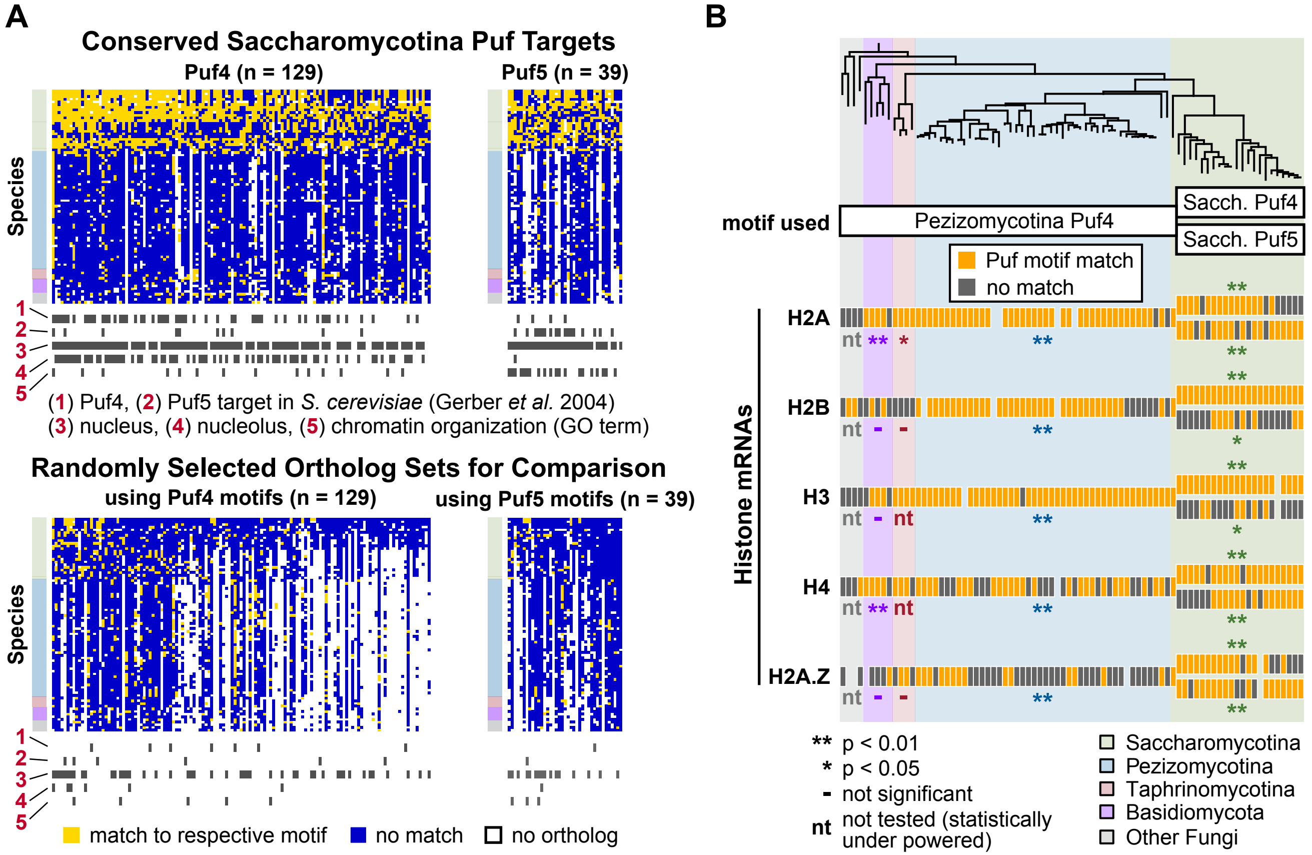

Supp. Figure 24
