# Supplementary material for: Evolutionary Conservation and Diversification of Puf RNA Binding Proteins and Their mRNA Targets
Source: PLoS Biol. 2015 Nov 20;13(11):e1002307. doi: 10.1371/journal.pbio.1002307 (PMC4654594; doi:10.1371/journal.pbio.1002307)
Supplement: S12 Table — The conservation and changes in Puf-RNA evolution draw several parallels to observations made within the extensively studied evolution of transcription factors and their targets. In the table, we summarize the analogous changes and cite examples from the transcription factor literature [11,64,123,147–159] for comparison. (PPTX) [file pbio.1002307.s047.pptx]

## Slide 1
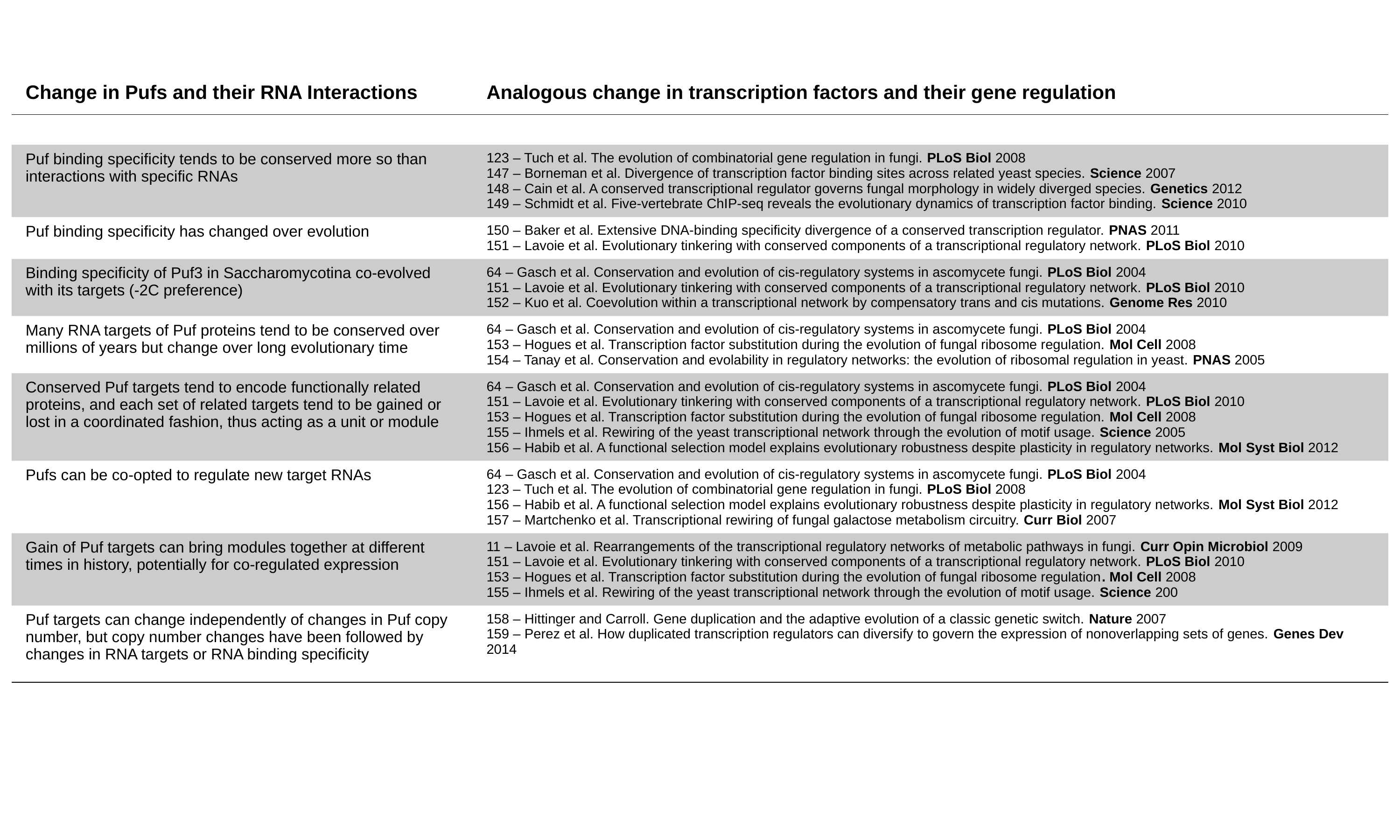

| Change in Pufs and their RNA Interactions | Analogous change in transcription factors and their gene regulation |
| --- | --- |
| | |
| Puf binding specificity tends to be conserved more so than interactions with specific RNAs | 123 – Tuch et al. The evolution of combinatorial gene regulation in fungi. PLoS Biol 2008 147 – Borneman et al. Divergence of transcription factor binding sites across related yeast species. Science 2007 148 – Cain et al. A conserved transcriptional regulator governs fungal morphology in widely diverged species. Genetics 2012 149 – Schmidt et al. Five-vertebrate ChIP-seq reveals the evolutionary dynamics of transcription factor binding. Science 2010 |
| Puf binding specificity has changed over evolution | 150 – Baker et al. Extensive DNA-binding specificity divergence of a conserved transcription regulator. PNAS 2011 151 – Lavoie et al. Evolutionary tinkering with conserved components of a transcriptional regulatory network. PLoS Biol 2010 |
| Binding specificity of Puf3 in Saccharomycotina co-evolved with its targets (-2C preference) | 64 – Gasch et al. Conservation and evolution of cis-regulatory systems in ascomycete fungi. PLoS Biol 2004 151 – Lavoie et al. Evolutionary tinkering with conserved components of a transcriptional regulatory network. PLoS Biol 2010 152 – Kuo et al. Coevolution within a transcriptional network by compensatory trans and cis mutations. Genome Res 2010 |
| Many RNA targets of Puf proteins tend to be conserved over millions of years but change over long evolutionary time | 64 – Gasch et al. Conservation and evolution of cis-regulatory systems in ascomycete fungi. PLoS Biol 2004 153 – Hogues et al. Transcription factor substitution during the evolution of fungal ribosome regulation. Mol Cell 2008 154 – Tanay et al. Conservation and evolability in regulatory networks: the evolution of ribosomal regulation in yeast. PNAS 2005 |
| Conserved Puf targets tend to encode functionally related proteins, and each set of related targets tend to be gained or lost in a coordinated fashion, thus acting as a unit or module | 64 – Gasch et al. Conservation and evolution of cis-regulatory systems in ascomycete fungi. PLoS Biol 2004 151 – Lavoie et al. Evolutionary tinkering with conserved components of a transcriptional regulatory network. PLoS Biol 2010 153 – Hogues et al. Transcription factor substitution during the evolution of fungal ribosome regulation. Mol Cell 2008 155 – Ihmels et al. Rewiring of the yeast transcriptional network through the evolution of motif usage. Science 2005 156 – Habib et al. A functional selection model explains evolutionary robustness despite plasticity in regulatory networks. Mol Syst Biol 2012 |
| Pufs can be co-opted to regulate new target RNAs | 64 – Gasch et al. Conservation and evolution of cis-regulatory systems in ascomycete fungi. PLoS Biol 2004 123 – Tuch et al. The evolution of combinatorial gene regulation in fungi. PLoS Biol 2008 156 – Habib et al. A functional selection model explains evolutionary robustness despite plasticity in regulatory networks. Mol Syst Biol 2012 157 – Martchenko et al. Transcriptional rewiring of fungal galactose metabolism circuitry. Curr Biol 2007 |
| Gain of Puf targets can bring modules together at different times in history, potentially for co-regulated expression | 11 – Lavoie et al. Rearrangements of the transcriptional regulatory networks of metabolic pathways in fungi. Curr Opin Microbiol 2009 151 – Lavoie et al. Evolutionary tinkering with conserved components of a transcriptional regulatory network. PLoS Biol 2010 153 – Hogues et al. Transcription factor substitution during the evolution of fungal ribosome regulation. Mol Cell 2008 155 – Ihmels et al. Rewiring of the yeast transcriptional network through the evolution of motif usage. Science 200 |
| Puf targets can change independently of changes in Puf copy number, but copy number changes have been followed by changes in RNA targets or RNA binding specificity | 158 – Hittinger and Carroll. Gene duplication and the adaptive evolution of a classic genetic switch. Nature 2007 159 – Perez et al. How duplicated transcription regulators can diversify to govern the expression of nonoverlapping sets of genes. Genes Dev 2014 |
| | |
