# Supplementary material for: Evolutionary Conservation and Diversification of Puf RNA Binding Proteins and Their mRNA Targets
Source: PLoS Biol. 2015 Nov 20;13(11):e1002307. doi: 10.1371/journal.pbio.1002307 (PMC4654594; doi:10.1371/journal.pbio.1002307)
Supplement: S1 Text — (DOCX) [file pbio.1002307.s048.docx]

**S1 Text. Delineating the evolutionary history of Puf proteins.**

We identified and classified Puf proteins from the 99 eukaryote species present in the InParanoid 7 database [1]. The InParanoid database conveniently contains predicted protein sequences for all included species, and species selected to be in the database have genome sequences of high quality and completeness [1].

To identify Puf proteins from each of the 99 eukaryote species, we performed an iterative BLASTP search using the known diverse Puf proteins from *S. cerevisiae* as the starting query and an E-value cutoff of 10^-5^. Sequences from hits in the first step were then used as the query for a second search for additional Puf proteins (see Materials and Methods for details). Puf proteins are unique to eukaryotes, and our search did not yield a hit when *E. coli* protein sequences were searched, providing one of several pieces of evidence that our search was specific. To further evaluate the performance of our search, we compared our results to annotated Pufs from species known to contain many Puf proteins. Our list of putative Puf proteins contained 12/12, 25/26, and 19/19 of the Pufs annotated in *C. elegans*, *A. thaliana*, and *O. sativa*, respectively [2-5]. The protein missed from *A. thaliana* was not found among the protein sequences retrieved from InParanoid. Our list contained three additional putative Pufs all from *O. sativa* out of the more than 10^5^ protein sequences searched for the three species. Based on the comparisons in these three species, our search had an estimated sensitivity of 100% and specificity of at least 99.9% (three possible false positives out of 10^5^). Overall, this search yielded 573 putative Puf proteins. Each species had at least one Puf protein and an average of six Puf proteins (S1 Fig., S1 Table).

Puf proteins were then classified as to whether they are orthologous to *S. cerevisiae* Puf proteins and to *N. crassa* Puf8 (S1 Fig., S1 Table). We chose *S. cerevisiae* as we planned to focus on fungi later in this work. As it turns out, *S. cerevisiae* Pufs well represent the diversity of Puf proteins found across eukaryotes. *S. cerevisiae* has seven Puf proteins: Puf1-6 and Nop9 (relationships shown in S22 Fig.). Puf1 and Puf2 are paralogs that arose from a whole genome duplication in yeast [6-7]. Puf4 and Puf5 are paralogs that arose from a duplication after the origin of Saccharomycotina (S1 Fig., S9 Fig., S10 FigA.). We also classified proteins as orthologs to *N. crassa* Puf8 as our initial phylogenetic analysis uncovered that its gene was deleted in a yeast ancestor of *S. cerevisiae* (S1 Fig.). Puf8 appears to have been derived from a duplication of Puf3 (S22 Fig.).

We classified Puf proteins as orthologs based on a series of information: reciprocal best BLAST hits, the pattern of amino acids predicted to contact RNA bases, and phylogenetic analysis (see Materials and Methods for details). Of the full set of 573 Puf proteins, 522 (91.1%) were classified, leaving 51 proteins unassigned (S1 Fig., S1 Table). The unassigned proteins are composed of proteins that we could not confidently assign as an ortholog to a specific Puf and proteins that bear little resemblance to any of the Pufs used as a reference (*i.e*., do not appear to be orthologs).

The number of Puf proteins within each class and species varies, indicating Puf copy number has changed (S1 Fig.). Based on the types and number of Puf proteins in each species, we can identify events in the history of Puf proteins. We inferred over the phylogeny when a duplication or deletion has occurred based on parsimony (S1 Fig.). We note only events that are supported by three or more species, so the map presented is not expected to be comprehensive.

According to our Puf classification, proteins related to each of four Pufs (Puf3, Puf4, Puf6, Nop9) existed at a time close to the origin of eukaryotes (S1 Fig.). A protein related to Puf3, Puf6, or Nop9 is present in most species, including those that diverged early (are basal) in the eukaryote phylogeny. A protein related to Puf4 is also found among most of the basal eukaryote species. While a Puf4 protein is found in basal eukaryote species, plants, and fungi, Puf4 is not found in metazoans and the choanoflagellate *M. brevicollis*, suggesting that Puf4 was deleted in an ancestor of metazoans before their origin. Due to uncertainty in the root of eukaryotes, we did not attempt to resolve the origin of Puf proteins more precisely.

Plant, fungal, and worm lineages experienced an expansion in the number of Pufs (S1 Fig.). Duplication of a Puf protein provides an opportunity for changes in the relationship between the proteins and the RNAs they bind. In general, duplication could allow a change RNA binding specificity of one copy, a split in the ancestral target set or shared binding, and/or a change in the regulation of the Puf protein such as expression under a different condition or in a different cell type. The patterns of expansion of Puf proteins in these three lineages are different from each other with the exception that Puf6 and Nop9 have not expanded in any of the lineages. Others have studied the duplicated Pufs in these lineages [2-4,8], and we focus on what was previously not known.

An ancestor to fungi contained Puf3, Puf4, Puf6, and Nop9, and around the time of the origin of fungi, Puf1 and Puf8 appeared (S1 Fig., S8 FigA.). Puf1 is more similar to Puf3 and Puf4 than to Puf6 or Nop9 (S22 Fig.). Puf1 and its orthologs contain a second RNA-binding domain, an RNA recognition motif (RRM) (S8 FigB, S1 Table and S2 Table), suggesting that the RRM was present at or close to the time the Puf1 gene was created. Puf1 may have originated from the fusion of two existing genes, one of which was Puf3 or Puf4.

Puf8 may be a product of a duplication of Puf3 (S22 Fig.). The pattern of amino acids predicted to contact RNA bases is conserved among Puf8 proteins and is different from Puf3 (S10 FigC, S1 Table and S2 Table), suggesting that the binding specificity of Puf8 diverged shortly after its creation to a specificity that has since been conserved. Puf8 was deleted in an ancestor to most of the Saccharomycotina species. We found an ortholog of Puf8 in *L. starkeyi* (S2 Table), a species basal to the Saccharomycotina species used here. *Y. lipolytica* also contains an ortholog to Puf8, but what would be the C-terminal portion of the Puf RNA binding domain of *Y. lipolytica* Puf8 differs substantially from other Puf8 proteins. This suggests that the RNA binding specificity of *Y. lipolytica* Puf8 has diverged from its orthologs.

The nematode *C. elegans* contains 12 Puf proteins [4-5]. In addition to Puf6 and Nop9 orthologs, *C. elegans* has two Puf3 orthologs Puf8 and Puf9 that share the same pattern of amino acids that contact RNA (S1 Table). Puf8 has been shown to have a similar binding specificity as *S. cerevisiae* Puf3 and the Pumilio proteins of higher eukaryotes [9-11]. *C. elegans* also contains seven Puf proteins (Fbf-1/2, Puf-3/4/5/6/7/11) that are distinct from Pufs outside the worm lineage. Of the Pufs predicted to be present in the ancestor of worms, these Pufs bear the highest resemblance to Puf3, so we assigned these worm Pufs as orthologs to Puf3 under a model that the expansion originated with a duplication of Puf3 (S1 Fig.). Previous work demonstrated that these seven Puf proteins in *C. elegans* have substantially diverged from Puf3 in terms of primary sequence and binding specificity [9-14].

**References**

1. Ostlund G, Schmitt T, Forslund K, Kostler T, Messina DN, Roopra S, et al. InParanoid 7: new algorithms and tools for eukaryotic orthology analysis. Nucleic Acids Res. 2010 Jan;38(Database issue):D196-203.

2. Francischini CW, Quaggio RB. Molecular characterization of Arabidopsis thaliana PUF proteins--binding specificity and target candidates. FEBS J. 2009 Oct;276(19):5456-70.

3. Tam PP, Barrette-Ng IH, Simon DM, Tam MW, Ang AL, Muench DG. The Puf family of RNA-binding proteins in plants: phylogeny, structural modeling, activity and subcellular localization. BMC Plant Biol. 2010;10:44.

4. Wickens M, Bernstein DS, Kimble J, Parker R. A PUF family portrait: 3'UTR regulation as a way of life. Trends Genet. 2002 Mar;18(3):150-7.

5. Liu Q, Stumpf C, Thomas C, Wickens M, Haag ES. Context-dependent function of a conserved translational regulatory module. Development. 2012 Apr;139(8):1509-21.

6. Byrne KP, Wolfe KH. The Yeast Gene Order Browser: combining curated homology and syntenic context reveals gene fate in polyploid species. Genome Res. 2005 Oct;15(10):1456-61.

7. Kellis M, Birren BW, Lander ES. Proof and evolutionary analysis of ancient genome duplication in the yeast Saccharomyces cerevisiae. Nature. 2004 Apr 8;428(6983):617-24.

8. Abbasi N, Park YI, Choi SB. Pumilio Puf domain RNA-binding proteins in Arabidopsis. Plant Signal Behav. 2011 Mar;6(3):364-8.

9. Opperman L, Hook B, DeFino M, Bernstein DS, Wickens M. A single spacer nucleotide determines the specificities of two mRNA regulatory proteins. Nat Struct Mol Biol. 2005 Nov;12(11):945-51.

10. Stumpf CR, Kimble J, Wickens M. A Caenorhabditis elegans PUF protein family with distinct RNA binding specificity. RNA. 2008 Aug;14(8):1550-7.

11. Campbell ZT, Bhimsaria D, Valley CT, Rodriguez-Martinez JA, Menichelli E, Williamson JR, et al. Cooperativity in RNA-protein interactions: global analysis of RNA binding specificity. Cell Rep. 2012 May 31;1(5):570-81.

12. Kershner AM, Kimble J. Genome-wide analysis of mRNA targets for Caenorhabditis elegans FBF, a conserved stem cell regulator. Proc Natl Acad Sci U S A. 2010 Feb 23;107(8):3936-41.

13. Bernstein D, Hook B, Hajarnavis A, Opperman L, Wickens M. Binding specificity and mRNA targets of a C. elegans PUF protein, FBF-1. RNA. 2005 Apr;11(4):447-58.

14. Koh YY, Opperman L, Stumpf C, Mandan A, Keles S, Wickens M. A single C. elegans PUF protein binds RNA in multiple modes. RNA. 2009 Jun;15(6):1090-9.
