# Supplementary material for: Evolutionary Conservation and Diversification of Puf RNA Binding Proteins and Their mRNA Targets
Source: PLoS Biol. 2015 Nov 20;13(11):e1002307. doi: 10.1371/journal.pbio.1002307 (PMC4654594; doi:10.1371/journal.pbio.1002307)
Supplement: S2 Text — (DOCX) [file pbio.1002307.s049.docx]

**S2 Text. Orthologs of Puf3 proteins share a conserved RNA binding interface.**

We assessed whether Puf3 orthologs have these conserved RNA-contacting residues and whether the amino acids that surround these residues and form the RNA binding pocket are also conserved, and we used this information to infer whether binding specificity was conserved.

Puf3 orthologs were identified in 93 of 99 species (green or gray in S2 FigA); no Puf3 ortholog could be identified from protein or genome sequences in the six remaining species (white-only bars in S2 FigA). A multiple sequence alignment of Puf3 orthologs revealed that 82 of the 93 species had a Puf3 ortholog with all 24 of the RNA-base-contacting amino acids identical to those of *S. cerevisiae* Puf3 (in green in S2 FigA). In five of the eleven remaining species, only one of these 24 amino acids diverged from *S. cerevisiae* Puf3.

To compare the conservation of each Puf3 residue and particularly those within the RNA binding domain, we visualized residue conservation in the context of domain annotations and the three-dimensional structure of the Puf RNA binding domain. Residues within the Puf repeats are more conserved than the rest of the protein; repeat residues are on average 71.1% percent identical while residues outside of the Puf repeats average 27.2% identity (S2 FigC). Fifty-four out of the 287 residues comprising the Puf repeats, including the 24 RNA-contacting residues, were identical in more than 95% of the Puf3s in this sample; 62 of the Puf-repeat residues were shared in less than 50% of species (S2 FigB). Mapping the conservation of residues onto a published structure of *S. cerevisiae* Puf3 [1] reveals that residues on the inner surface are most highly conserved and form a distinctly conserved pocket around the bound RNA, whereas residues outside of the RNA binding pocket are more variable (S2 FigD).

**References**

1. Zhu D, Stumpf CR, Krahn JM, Wickens M, Hall TM. A 5' cytosine binding pocket in Puf3p specifies regulation of mitochondrial mRNAs. Proc Natl Acad Sci U S A. 2009 Dec 1;106(48):20192-7.
