# Supplementary material for: Evolutionary Conservation and Diversification of Puf RNA Binding Proteins and Their mRNA Targets
Source: PLoS Biol. 2015 Nov 20;13(11):e1002307. doi: 10.1371/journal.pbio.1002307 (PMC4654594; doi:10.1371/journal.pbio.1002307)
Supplement: S3 Text — (DOCX) [file pbio.1002307.s050.docx]

**S3 Text. Characterization of the binding specificity of *N. crassa* Puf3.**

We expressed the Puf3 protein from the Pezizomycotina species *Neurospora crassa* fused with a tandem affinity purification (TAP) tag in a *S. cerevisiae* strain missing five endogenous Puf proteins, Puf1-5 (5Δ*pufs* strain [1], Materials and Methods). The absence of endogenous *S. cerevisiae* Puf proteins eliminated their competition for shared RNA binding sites. We affinity purified *N. crassa* Puf3-TAP from extracts of exponentially growing *S. cerevisiae* cells and identified the RNAs enriched in association with the Puf3 fusion protein, compared to a mock control, following protocols that have been used routinely to identify targets of RNA binding proteins (Materials and Methods) [2-4]. We performed the *N. crassa* Puf3 purifications in parallel with *S. cerevisiae* Puf3 purifications for direct comparison.

We found 250 RNAs significantly associated with *N. crassa* Puf3 and 392 with *S. cerevisiae* Puf3 using a Significance Analysis of Microarrays (SAM) test with a 1% local false discovery rate (FDR) cutoff. *N. crassa* Puf3 *and S. cerevisiae* Puf3 proteins bound to 83 RNAs in common, much more than expected by chance (odds-ratio = 8.4, p = 10^-37^ by Fisher's exact test) and suggesting that *N. crassa* Puf3 might recognize sequences similar to those recognized by *S. cerevisiae* Puf3. We used the programs FIRE [5] and REFINE [3,6] to search for sequence patterns enriched in the 3' UTRs of RNAs associated with each of the two Puf3 proteins relative to 3' UTRs of all RNAs. RNAs associated with *N. crassa* Puf3 were enriched for sequences matching the core eight-nucleotide motif preferred by *S. cerevisiae* Puf3 and its orthologs (Fig. 3A, S6 Fig.).

As expected, the mRNA targets of *S. cerevisiae* Puf3 were enriched with sequences that include the conserved core sequence and a preference for C two positions upstream of the UGUA, matching the characterized Puf3 binding specificity (Fig. 4A). In contrast, *N. crassa* Puf3 did not display a preference for particular nucleotides outside of the core eight-nucleotide sequence. Further analysis suggests that the preference for an upstream C was acquired within the history of Saccharomycotina (S7 Fig.). As this additional feature of RNA recognition was gained after Saccharomycotina diverged from Pezizomycotina (S7 Fig.), it cannot account for Puf3’s concerted acquisition of RNAs encoding mitochondrial proteins, which occurred before the origin of Saccharomycotina.

**References**

1. Olivas W, Parker R. The Puf3 protein is a transcript-specific regulator of mRNA degradation in yeast. EMBO J. 2000 Dec 1;19(23):6602-11.

2. Gerber AP, Herschlag D, Brown PO. Extensive association of functionally and cytotopically related mRNAs with Puf family RNA-binding proteins in yeast. PLoS Biol. 2004 Mar;2(3):E79.

3. Hogan DJ, Riordan DP, Gerber AP, Herschlag D, Brown PO. Diverse RNA-binding proteins interact with functionally related sets of RNAs, suggesting an extensive regulatory system. PLoS Biol. 2008 Oct 28;6(10):e255.

4. Tsvetanova NG, Klass DM, Salzman J, Brown PO. Proteome-wide search reveals unexpected RNA-binding proteins in Saccharomyces cerevisiae. PLoS One. 2010;5(9).

5. Elemento O, Slonim N, Tavazoie S. A universal framework for regulatory element discovery across all genomes and data types. Mol Cell. 2007 Oct 26;28(2):337-50.

6. Riordan DP, Herschlag D, Brown PO. Identification of RNA recognition elements in the Saccharomyces cerevisiae transcriptome. Nucleic Acids Res. 2011 Mar;39(4):1501-9.
