# Supplementary material for: Evolutionary Conservation and Diversification of Puf RNA Binding Proteins and Their mRNA Targets
Source: PLoS Biol. 2015 Nov 20;13(11):e1002307. doi: 10.1371/journal.pbio.1002307 (PMC4654594; doi:10.1371/journal.pbio.1002307)
Supplement: S4 Text — (DOCX) [file pbio.1002307.s051.docx]

**S4 Text. Analysis of conserved binding sites identifies novel targets of *S. cerevisiae* Puf3, Puf4, and Puf5.**

As *S. cerevisiae* Puf3 targets have been studied using several, different genome-wide assays, we can test if members of the conserved Saccharomycotina Puf3 targets not included in the Gerber *et al.* *S. cerevisiae* Puf3 target set represent *bona fide* targets. The analysis described below supports the conclusion that putative Puf3 targets identified through binding sequence conservation are indeed Puf3 targets.

As described in the Results and Materials and Methods, we identified 276 ortholog sets whose mRNAs tend to contain Puf3 binding sites in Saccharomycotina. For these 276 ortholog sets, 231 (84%) mRNAs had motif matches in their 3' UTRs in *S. cerevisiae* (S18 FigA). Of these 231 mRNAs, 142 (61%, odds-ratio = 100, p = 10^-162^) were experimentally identified as Puf3 targets by Gerber *et al*. [1], revealing 89 mRNAs as putative, novel Puf3 targets in *S. cerevisiae* (S18 FigA). Known Puf3 targets tend to encode for proteins that function in the mitochondrion. Of the 276 conserved Saccharomycotina Puf3 targets, 256 are annotated to "mitochondrion" (93%, odds-ratio = 72, p = 10^-171^), including 81 out of the 89 putative, novel targets (91%, odds-ratio = 45, p = 10^-49^, S18 FigA).

Three additional, independent pieces of evidence support the conclusion that the putative, novel Puf3 targets represent *bona fide* targets. Although each novel target on its own did not meet the cutoff for being called a target by Gerber *et al.*, the novel targets were enriched as a group in the Gerber *et al.* affinity purification data [1] used to identify the already known targets (p = 10^-25^, S18 FigB). In line with the observation that Puf3 interacts with components of the decay machinery to promote the decay of its targets, the novel Puf3 targets increase in RNA abundance in Puf3 knockout cells (p = 10^-7^) and to the same extent as known Puf3 targets (p = 0.41, S18 FigC). Finally, the novel Puf3 target RNAs are enriched when mitochondria are isolated from yeast cells (p = 10^-14^, S18 FigD), and the extent of this enrichment decreases in Puf3 knockout cells (p = 10^-4^, S18 FigE).

We also show in S7 Text that conserved targets of Puf4 and Puf5 in Saccharomycotina overlap extensively with those defined experimentally but include many additional members. We identified novel targets of *S. cerevisiae* Puf4 or Puf5 using the same approach as described above for Puf3. Of the 129 conserved Saccharomycotina Puf4 targets, 38 contain a Puf4 motif match in *S. cerevisiae* but were not experimentally identified as a Puf4 target. Like the experimentally identified *S. cerevisiae* Puf4 targets, these novel targets were highly likely to encode for nucleolar proteins (26/38, 68%, odds-ratio = 48, p = 10^-22^) and proteins involved in ribosome biogenesis (18/38, 47%, odds-ratio = 15, p = 10^-9^). Of the 39 conserved Saccharomycotina Puf5 targets, nine contain a Puf5 motif match in *S. cerevisiae* but were not experimentally identified as a Puf5 target. Like the experimentally identified *S. cerevisiae* Puf5 targets, the majority of these targets encode nuclear proteins (8/9, 89%, statistically underpowered) and proteins involved in chromatin organization (6/9, 67%, odds-ratio = 47, p = 0.003).

**References**

1. Gerber AP, Herschlag D, Brown PO. Extensive association of functionally and cytotopically related mRNAs with Puf family RNA-binding proteins in yeast. PLoS Biol. 2004 Mar;2(3):E79.
