# Supplementary material for: Evolutionary Conservation and Diversification of Puf RNA Binding Proteins and Their mRNA Targets
Source: PLoS Biol. 2015 Nov 20;13(11):e1002307. doi: 10.1371/journal.pbio.1002307 (PMC4654594; doi:10.1371/journal.pbio.1002307)
Supplement: S5 Text — (DOCX) [file pbio.1002307.s052.docx]

**S5 Text. Predicting which Pezizomycotina Puf proteins bind UGUA-based motifs.**

Puf proteins whose binding specificity has been characterized and found to bind UGUA-based motifs contain eight Puf repeats and particular amino acids that interact with the UGUA bases (S10 FigB-C). Pezizomycotina species contain three Puf proteins (Puf3, Puf4, and Puf8) that could recognize UGUA-based motifs based on established patterns relating specific amino acid residues in Puf proteins to their binding specificity. The Pezizomycotina Puf3 and Puf4 proteins contain the same conserved RNA-contacting amino acids as their orthologs in non-Pezizomycotina species (S10 FigC, S2 Table). We have named an uncharacterized protein “Puf8”, which is found in Pezizomycotina species among other fungi (S9 Fig.). Puf8 contains eight Puf repeats, is closest in overall sequence identity to Puf3 (S22 Fig.), and also contains some of the amino acids observed in other Pufs that interact with UGUA-based sequences (S10 FigC). Puf8 also has several amino acids predicted to interact with RNA that have not been observed in other characterized Puf proteins: a lysine (K), tryptophan (W), leucine (L), phenylalanine (F), and isoleucine (I) at the position in each repeat predicted to form stacking interactions with the RNA bases (S10 FigC); tryptophan and phenylalanine were identified in a previous screen for amino acids that could be substituted for a known stacking amino acid and still permit RNA binding [1].

Pezizomycotina species also contain several Puf proteins that are unlikely to bind UGUA-based motifs (Puf1, Puf6, Nop9). These proteins contain fewer than eight canonical Puf repeats (S10 FigB) and lack the specific constellation of amino acid residues known to interact with UGUA nucleotides (S10 FigC, S2 Table). Orthologs of Pezizomycotina Puf1 in *S. cerevisiae* recognize a different sequence motif; *S. cerevisiae* Puf1 and its paralog Puf2 recognize sequences containing UAAU [2-4]. Puf1 proteins also contain an RRM domain, and the nature of the RNA-protein interactions is unknown. *S. cerevisiae* Puf6 displays degenerate binding and little preference for a site containing UGUA [5]. The binding specificity of Nop9 is not known for any species. The localization and function of Nop9 in distantly related eukaryotes suggests it has a conserved function in the nucleolus, as *S. cerevisiae* Nop9 and its orthologs in the plant *A. thaliana* (APUM23) and trypanosome *T. brucei* (TbPUF7) are nucleolar proteins that function in rRNA biogenesis [6-8].

**References**

1. Koh YY, Wang Y, Qiu C, Opperman L, Gross L, Tanaka Hall TM, et al. Stacking interactions in PUF-RNA complexes. RNA. 2011 Apr;17(4):718-27.

2. Hogan DJ, Riordan DP, Gerber AP, Herschlag D, Brown PO. Diverse RNA-binding proteins interact with functionally related sets of RNAs, suggesting an extensive regulatory system. PLoS Biol. 2008 Oct 28;6(10):e255.

3. Riordan DP, Herschlag D, Brown PO. Identification of RNA recognition elements in the Saccharomyces cerevisiae transcriptome. Nucleic Acids Res. 2011 Mar;39(4):1501-9.

4. Yosefzon Y, Koh YY, Chritton JJ, Lande A, Leibovich L, Barziv L, et al. Divergent RNA binding specificity of yeast Puf2p. RNA. 2011 Aug;17(8):1479-88.

5. Muller M, Heym RG, Mayer A, Kramer K, Schmid M, Cramer P, et al. A cytoplasmic complex mediates specific mRNA recognition and localization in yeast. PLoS Biol. 2011 Apr;9(4):e1000611.

6. Abbasi N, Kim HB, Park NI, Kim HS, Kim YK, Park YI, et al. APUM23, a nucleolar Puf domain protein, is involved in pre-ribosomal RNA processing and normal growth patterning in Arabidopsis. Plant J. 2010 Dec;64(6):960-76.

7. Droll D, Archer S, Fenn K, Delhi P, Matthews K, Clayton C. The trypanosome Pumilio-domain protein PUF7 associates with a nuclear cyclophilin and is involved in ribosomal RNA maturation. FEBS Lett. 2010 Mar 19;584(6):1156-62.

8. Thomson E, Rappsilber J, Tollervey D. Nop9 is an RNA binding protein present in pre-40S ribosomes and required for 18S rRNA synthesis in yeast. RNA. 2007 Dec;13(12):2165-74.
