# Supplementary material for: Evolutionary Conservation and Diversification of Puf RNA Binding Proteins and Their mRNA Targets
Source: PLoS Biol. 2015 Nov 20;13(11):e1002307. doi: 10.1371/journal.pbio.1002307 (PMC4654594; doi:10.1371/journal.pbio.1002307)
Supplement: S6 Text — (DOCX) [file pbio.1002307.s053.docx]

**S6 Text. The Diversification of Puf4 and its targets in Pezizomycotina.**

Puf4 in the Leotiomyceta has taken on 441 conserved binding partners that are not shared by Puf3 in the Saccharomycotina lineage (S15 Fig.), an observation that further supports selected reprogramming of the Puf4 targets in Leotiomyceta as opposed to changes resulting solely from drift. Many of these 441 RNAs encode proteins linked to the mitochondrion in *N. crassa* (147 of the 441, 33%), including RNAs that encode components of the tricarboxylic acid (TCA) cycle and electron transport chain complexes III and V (S23 Fig.). Thus, Pezizomycotina Puf4 appears to play an additional mitochondrial role. The targets unique to Leotiomyceta Puf4 also included RNAs apparently unrelated to mitochondrial functions. For example, almost half of the RNAs encoding subunits of the proteasome are putative conserved Leotiomyceta Puf4 targets, whereas the Saccharomycotina Puf3 targets do not include any RNAs encoding proteasome proteins (S23 Fig.).
