# Supplementary material for: Evolutionary Conservation and Diversification of Puf RNA Binding Proteins and Their mRNA Targets
Source: PLoS Biol. 2015 Nov 20;13(11):e1002307. doi: 10.1371/journal.pbio.1002307 (PMC4654594; doi:10.1371/journal.pbio.1002307)
Supplement: S7 Text — (DOCX) [file pbio.1002307.s054.docx]

**S7 Text. Saccharomycotina Puf4 targets diverged after the Puf4/Puf5 duplication.**

In *S. cerevisiae,* the paralogs Puf4 and Puf5 bind RNA target sets that are almost entirely distinct [1]; these targets sets are also distinct from the inferred targets of Puf4 in Pezizomycotina fungi. Puf4 and Puf5 arose from a gene duplication early in the Saccharomycotina lineage (S17 Text, Fig. 5B and S8 Fig.). We wanted to know when in the Saccharomycotina lineage the targets of Puf4 and Puf5 began to diverge. To infer the targets of Puf4 and Puf5, respectively, that are conserved among the Saccharomycotina, we used the set of motifs that represent each protein's RNA binding specificity in *S. cerevisiae* (Fig. 5B, Materials and Methods). By this criterion, we found significant conservation of targets for both proteins in the Saccharomycotina lineage.

Puf4 sites were conserved (≤1% FDR) in the 3' UTRs of 129 Saccharomycotina orthologous gene sets (S24 FigA). Conserved Saccharomycotina Puf4 targets inferred based on the Puf4 recognition motif overlapped those identified experimentally in *S. cerevisiae* (53/129, 41%, odds-ratio = 27, p = 10^-45^). Puf5 sites were also conserved (≤1% FDR) in the 3' UTRs of 39 Saccharomycotina ortholog sets; among this set of orthologous genes inferred based on the Puf5-recognition motif, 17 correspond to known Puf5 targets in *S. cerevisiae* (44% of conserved targets, odds-ratio = 25, p = 10^-15^, S24 FigA).

The conserved Saccharomycotina Puf4 and Puf5 targets recapitulate functional themes observed for *S. cerevisiae* Puf4 and Puf5 targets. The majority of conserved Saccharomycotina Puf4 targets encode proteins that function in the nucleus (109/129, 84%, odds-ratio = 9.5, p = 10^-24^) and more specifically in the nucleolus (70/129, 54%, odds-ratio = 29, p = 10^-54^, S24 FigA). This result extends and strengthens a theme observed for *S. cerevisiae* Puf4 targets (49/205 nucleolar proteins, 24%). The nucleolar proteins encoded by putative Puf4 targets are largely involved in ribosome biogenesis, including ribosomal RNA production and processing.

The majority of conserved Saccharomycotina Puf5 targets also encode proteins that function in the nucleus (36/39, 92%, odds-ratio = 20, p = 10^-8^), but unlike Puf4's conserved targets most are involved in chromatin organization (20/39, 51%, odds-ratio = 24, p = 10^-12^, S24 FigA). All three of the RNAs conserved as Puf5 targets that do not encode nuclear proteins instead encode proteins that localize, at least in part, to the bud neck or tip in *S. cerevisiae* (Bud2, Lrg1, Sac6, see references at *Saccharomyces* Genome Database [2]).

The evidence for conserved Puf4 and Puf5 RNA targets throughout the Saccharomycotina lineage indicates that these targets were gained in an ancestor of *S. cerevisiae*. To learn about the timing of the emergence of these RNA targets, we evaluated whether putative binding sites for Puf4 or Puf5 were found in related sets of RNAs in species spanning Saccharomycotina that contain Puf4 and Puf5. The observation of statistical significance across all of these species (S24 FigA) suggests that Puf4 and Puf5 started binding many of their respective RNA targets at or near the time Puf4 duplicated to give Puf4 and Puf5 (Fig. 8, star at #3).

The observed distinction between Puf4 and Puf5 targets raises the question of whether the diversification in RNA targets resulted from splitting a larger pool of RNAs originally bound by the ancestral Puf4 protein or, alternatively, one of the duplicated Puf proteins acquired a novel set of targets after the duplication event. Supporting the model that the ancestral Puf4 had a larger set of target genes, which were partitioned between Puf4 and Puf5 after its duplication, we identified modest commonalities between the conserved Puf4 or Puf5 targets and the putative Puf4 targets in *Yarrowia lipolytica*, the basal Saccharomycotina species used here (S9 Text), and an additional striking commonality is described in the next section. Data from more species that diverged early in the expansion of the Saccharomycotina lineage will be needed to fully evaluate this aspect of Puf/RNA evolutionary history.

**References**

1. Gerber AP, Herschlag D, Brown PO. Extensive association of functionally and cytotopically related mRNAs with Puf family RNA-binding proteins in yeast. PLoS Biol. 2004 Mar;2(3):E79.

2. Cherry JM, Hong EL, Amundsen C, Balakrishnan R, Binkley G, Chan ET, et al. Saccharomyces Genome Database: the genomics resource of budding yeast. Nucleic Acids Res. 2012 Jan;40(Database issue):D700-5.
