# Supplementary material for: Evolutionary Conservation and Diversification of Puf RNA Binding Proteins and Their mRNA Targets
Source: PLoS Biol. 2015 Nov 20;13(11):e1002307. doi: 10.1371/journal.pbio.1002307 (PMC4654594; doi:10.1371/journal.pbio.1002307)
Supplement: S8 Text — (DOCX) [file pbio.1002307.s055.docx]

**S8 Text. Histone mRNAs are ancient targets of Puf4 and Puf5.**

*S. cerevisiae* Puf4 and Puf5 bind a small set of RNAs in common, including the RNAs encoding the four core histone proteins (H2A, H2B, H3, H4) [1], and three of the five common conserved Puf4 and Puf5 targets in Saccharomycotina encode histone proteins (H2A, H4, H2A.Z; the other two RNAs common to the inferred Puf4 and Puf5 target sets are RPB8 and STH1). The conserved Pezizomycotina Puf4 targets also include the RNAs coding for the four core histones, suggesting that Puf4's interactions with histone mRNAs dates back at least to the common ancestor of these lineages (S8 Dataset).

To identify whether the histone RNA/Puf4 interactions date back even earlier, we tested whether RNAs encoding histones in Saccharomycotina, Pezizomycotina, and two additional fungal lineages contained a Puf4 motif match more often than expected by chance. For Saccharomycotina we tested for enrichment with either the Puf4 or Puf5 motifs, whereas for the other lineages we tested for enrichment of the Pezizomycotina Puf4 motif (see S24 FigB legend and Materials and Methods). Both Puf4 and Puf5 motifs were enriched among all five sets of histones transcripts (H2A, H2B, H3, H4, H2A.Z) present in Saccharomycotina fungi (S24 FigB), extending our results above and implicating Puf4 and Puf5 in the conserved regulation of all of these histone RNAs. The Pezizomycotina Puf4 motif was significantly enriched in RNAs encoding all five histones in Pezizomycotina (S24 FigB). Despite sparse sampling of Taphrinomycotina and Basidiomycota genomes, we found that the Pezizomycotina Puf4 motif was significantly enriched in H2A transcripts in both Taphrinomycotina and Basidiomycota fungi and in H4 transcripts in Basidiomycota (S24 FigB); additional histone RNAs may be revealed as Puf4 targets when more genome sequences become available in these clades.

**References**

1. Gerber AP, Herschlag D, Brown PO. Extensive association of functionally and cytotopically related mRNAs with Puf family RNA-binding proteins in yeast. PLoS Biol. 2004 Mar;2(3):E79.
