# Supplementary material for: Evolutionary Conservation and Diversification of Puf RNA Binding Proteins and Their mRNA Targets
Source: PLoS Biol. 2015 Nov 20;13(11):e1002307. doi: 10.1371/journal.pbio.1002307 (PMC4654594; doi:10.1371/journal.pbio.1002307)
Supplement: S9 Text — (DOCX) [file pbio.1002307.s056.docx]

**S9 Text. Evidence that Puf4 and Puf5 maintained a small number of ancestral targets after the Puf4 duplication.**

Puf4 or Puf5 targets in post-Puf4-duplication species may have interacted with the ancestral Puf4 protein prior to its duplication. We provide evidence in the Results and Discussion that Puf4’s interaction with histone mRNAs is broadly shared across fungi and likely was gained prior to the duplication of Puf4 and subsequently conserved. If additional targets of ancestral Puf4 have since been conserved, then putative targets in *Yarrowia lipolytica*, which is basal in Saccharomycotina and only contains one copy of Puf4, will share commonalities with the targets of Puf4 or Puf5 in post-duplication species. We explored this possibility using two approaches as described below and found evidence to support a model that Puf4 maintained some targets after its duplication.

In our first approach we identified conserved targets using Saccharomycotina fungi excluding *Y. lipolytica*. Then, assuming *Y. lipolytica* Puf4's binding specificity matches that of Pezizomycotina Puf4 (under the model that Pezizomycotina Puf4 motifs represent the ancestral binding specificity), we tested whether motif matches are found more likely than expected in *Y. lipolytica* RNAs related to Saccharomycotina Puf4 or Puf5 targets. Motif matches were modestly enriched in RNAs related to the conserved Puf4 (odds-ratio = 1.65, p = 0.01) or Puf5 targets (odds-ratio = 3.9, p = 0.007). We also searched for over-represented gene ontology categories among *Y. lipolytica* RNAs with a motif match in their 3' UTRs (S6 Dataset); nucleus (odds-ratio = 1.46, p = 0.0006) and chromatin organization (odds-ratio = 2.5, p = 0.0002) were among categories significantly enriched but nucleolus (odds-ratio = 1.28, p > 0.05) was not.

The results from the two analyses suggest *Y. lipolytica* Puf4 binds some RNAs related to targets of Puf4 and Puf5 in other Saccharomycotina species and that Puf4 bound a fraction of these RNAs in an ancestor to the Saccharomycotina species that predates the Puf4/5 duplication event.
