# Supplementary material for: Evolutionary Conservation and Diversification of Puf RNA Binding Proteins and Their mRNA Targets
Source: PLoS Biol. 2015 Nov 20;13(11):e1002307. doi: 10.1371/journal.pbio.1002307 (PMC4654594; doi:10.1371/journal.pbio.1002307)
Supplement: S10 Text — (DOCX) [file pbio.1002307.s057.docx]

**S10 Text. Puf proteins linked to diverse functions in different species.**

RNA binding proteins play important and diverse roles as evidenced at the organismal level by the phenotypes observed in mutants. The Puf proteins are involved in different processes across distantly related eukaryotes. In higher eukaryotes Puf proteins have been implicated in embryonic development [1-5], germ cell development and function [6-12], nerve cell growth and function [13-20], and additional roles [21-27]. In yeast the Puf proteins are involved in lifespan [28-30], mating [31-35], cell wall integrity [35-38], mitochondrial function and morphology [39-41], and other processes [38,42-44].

**References**

1. Barker DD, Wang C, Moore J, Dickinson LK, Lehmann R. Pumilio is essential for function but not for distribution of the Drosophila abdominal determinant Nanos. Genes Dev. 1992 Dec;6(12A):2312-26.

2. Gamberi C, Peterson DS, He L, Gottlieb E. An anterior function for the Drosophila posterior determinant Pumilio. Development. 2002 Jun;129(11):2699-710.

3. Lehmann R, Nussleinvolhard C. Involvement of the Pumilio Gene in the Transport of an Abdominal Signal in the Drosophila Embryo. Nature. 1987 Sep 10;329(6135):167-70.

4. Murata Y, Wharton RP. Binding of pumilio to maternal hunchback mRNA is required for posterior patterning in Drosophila embryos. Cell. 1995 Mar 10;80(5):747-56.

5. Wreden C, Verrotti AC, Schisa JA, Lieberfarb ME, Strickland S. Nanos and pumilio establish embryonic polarity in Drosophila by promoting posterior deadenylation of hunchback mRNA. Development. 1997 Aug;124(15):3015-23.

6. Asaoka-Taguchi M, Yamada M, Nakamura A, Hanyu K, Kobayashi S. Maternal Pumilio acts together with Nanos in germline development in Drosophila embryos. Nat Cell Biol. 1999 Nov;1(7):431-7.

7. Forbes A, Lehmann R. Nanos and Pumilio have critical roles in the development and function of Drosophila germline stem cells. Development. 1998 Feb;125(4):679-90.

8. Lin H, Spradling AC. A novel group of pumilio mutations affects the asymmetric division of germline stem cells in the Drosophila ovary. Development. 1997 Jun;124(12):2463-76.

9. Parisi M, Lin H. The Drosophila pumilio gene encodes two functional protein isoforms that play multiple roles in germline development, gonadogenesis, oogenesis and embryogenesis. Genetics. 1999 Sep;153(1):235-50.

10. Szakmary A, Cox DN, Wang Z, Lin H. Regulatory relationship among piwi, pumilio, and bag-of-marbles in Drosophila germline stem cell self-renewal and differentiation. Curr Biol. 2005 Jan 26;15(2):171-8.

11. Zhang B, Gallegos M, Puoti A, Durkin E, Fields S, Kimble J, et al. A conserved RNA-binding protein that regulates sexual fates in the C. elegans hermaphrodite germ line. Nature. 1997 Dec 4;390(6659):477-84.

12. Crittenden SL, Bernstein DS, Bachorik JL, Thompson BE, Gallegos M, Petcherski AG, et al. A conserved RNA-binding protein controls germline stem cells in Caenorhabditis elegans. Nature. 2002 Jun 6;417(6889):660-3.

13. Dubnau J, Chiang AS, Grady L, Barditch J, Gossweiler S, McNeil J, et al. The staufen/pumilio pathway is involved in Drosophila long-term memory. Curr Biol. 2003 Feb 18;13(4):286-96.

14. Mee CJ, Pym EC, Moffat KG, Baines RA. Regulation of neuronal excitability through pumilio-dependent control of a sodium channel gene. J Neurosci. 2004 Oct 6;24(40):8695-703.

15. Menon KP, Sanyal S, Habara Y, Sanchez R, Wharton RP, Ramaswami M, et al. The translational repressor Pumilio regulates presynaptic morphology and controls postsynaptic accumulation of translation factor eIF-4E. Neuron. 2004 Nov 18;44(4):663-76.

16. Muraro NI, Weston AJ, Gerber AP, Luschnig S, Moffat KG, Baines RA. Pumilio binds para mRNA and requires Nanos and Brat to regulate sodium current in Drosophila motoneurons. J Neurosci. 2008 Feb 27;28(9):2099-109.

17. Schweers BA, Walters KJ, Stern M. The Drosophila melanogaster translational repressor pumilio regulates neuronal excitability. Genetics. 2002 Jul;161(3):1177-85.

18. Siemen H, Colas D, Heller HC, Brustle O, Pera RA. Pumilio-2 function in the mouse nervous system. PLoS One. 2011;6(10):e25932.

19. Ye B, Petritsch C, Clark IE, Gavis ER, Jan LY, Jan YN. Nanos and Pumilio are essential for dendrite morphogenesis in Drosophila peripheral neurons. Curr Biol. 2004 Feb 17;14(4):314-21.

20. Kaye JA, Rose NC, Goldsworthy B, Goga A, L'Etoile ND. A 3'UTR pumilio-binding element directs translational activation in olfactory sensory neurons. Neuron. 2009 Jan 15;61(1):57-70.

21. Kuo MW, Wang SH, Chang JC, Chang CH, Huang LJ, Lin HH, et al. A novel puf-A gene predicted from evolutionary analysis is involved in the development of eyes and primordial germ-cells. PLoS One. 2009;4(3):e4980.

22. Fan CC, Lee LY, Yu MY, Tzen CY, Chou C, Chang MS. Upregulated hPuf-A promotes breast cancer tumorigenesis. Tumour Biol. 2013 Apr 28.

23. Kim SY, Kim JY, Malik S, Son W, Kwon KS, Kim C. Negative regulation of EGFR/MAPK pathway by Pumilio in Drosophila melanogaster. PLoS One. 2012;7(4):e34016.

24. Xu EY, Chang R, Salmon NA, Reijo Pera RA. A gene trap mutation of a murine homolog of the Drosophila stem cell factor Pumilio results in smaller testes but does not affect litter size or fertility. Mol Reprod Dev. 2007 Jul;74(7):912-21.

25. Wickens M, Bernstein DS, Kimble J, Parker R. A PUF family portrait: 3'UTR regulation as a way of life. Trends Genet. 2002 Mar;18(3):150-7.

26. Moore FL, Jaruzelska J, Fox MS, Urano J, Firpo MT, Turek PJ, et al. Human Pumilio-2 is expressed in embryonic stem cells and germ cells and interacts with DAZ (Deleted in AZoospermia) and DAZ-like proteins. Proc Natl Acad Sci U S A. 2003 Jan 21;100(2):538-43.

27. Salvetti A, Rossi L, Lena A, Batistoni R, Deri P, Rainaldi G, et al. DjPum, a homologue of Drosophila Pumilio, is essential to planarian stem cell maintenance. Development. 2005 Apr;132(8):1863-74.

28. Gotta M, Strahl-Bolsinger S, Renauld H, Laroche T, Kennedy BK, Grunstein M, et al. Localization of Sir2p: the nucleolus as a compartment for silent information regulators. EMBO J. 1997 Jun 2;16(11):3243-55.

29. Kennedy BK, Gotta M, Sinclair DA, Mills K, McNabb DS, Murthy M, et al. Redistribution of silencing proteins from telomeres to the nucleolus is associated with extension of life span in S. cerevisiae. Cell. 1997 May 2;89(3):381-91.

30. Stewart MS, Krause SA, McGhie J, Gray JV. Mpt5p, a stress tolerance- and lifespan-promoting PUF protein in Saccharomyces cerevisiae, acts upstream of the cell wall integrity pathway. Eukaryot Cell. 2007 Feb;6(2):262-70.

31. Blewett NH, Goldstrohm AC. A eukaryotic translation initiation factor 4E-binding protein promotes mRNA decapping and is required for PUF repression. Mol Cell Biol. 2012 Oct;32(20):4181-94.

32. Chen T, Kurjan J. Saccharomyces cerevisiae Mpt5p interacts with Sst2p and plays roles in pheromone sensitivity and recovery from pheromone arrest. Mol Cell Biol. 1997 Jun;17(6):3429-39.

33. Tadauchi T, Matsumoto K, Herskowitz I, Irie K. Post-transcriptional regulation through the HO 3'-UTR by Mpt5, a yeast homolog of Pumilio and FBF. EMBO J. 2001 Feb 1;20(3):552-61.

34. Xu BE, Skowronek KR, Kurjan J. The N terminus of Saccharomyces cerevisiae Sst2p plays an RGS-domain-independent, Mpt5p-dependent role in recovery from pheromone arrest. Genetics. 2001 Dec;159(4):1559-71.

35. Bourens M, Panozzo C, Nowacka A, Imbeaud S, Mucchielli MH, Herbert CJ. Mutations in the Saccharomyces cerevisiae kinase Cbk1p lead to a fertility defect that can be suppressed by the absence of Brr1p or Mpt5p (Puf5p), proteins involved in RNA metabolism. Genetics. 2009 Sep;183(1):161-73.

36. Kaeberlein M, Guarente L. Saccharomyces cerevisiae MPT5 and SSD1 function in parallel pathways to promote cell wall integrity. Genetics. 2002 Jan;160(1):83-95.

37. Ohkuni K, Kikuchi Y, Hara K, Taneda T, Hayashi N, Kikuchi A. Suppressor analysis of the mpt5/htr1/uth4/puf5 deletion in Saccharomyces cerevisiae. Mol Genet Genomics. 2006 Jan;275(1):81-8.

38. Traven A, Lo TL, Lithgow T, Heierhorst J. The yeast PUF protein Puf5 has Pop2-independent roles in response to DNA replication stress. PLoS One. 2010;5(5):e10651.

39. Fehrenbacher KL, Boldogh IR, Pon LA. A role for Jsn1p in recruiting the Arp2/3 complex to mitochondria in budding yeast. Mol Biol Cell. 2005 Nov;16(11):5094-102.

40. Garcia-Rodriguez LJ, Gay AC, Pon LA. Puf3p, a Pumilio family RNA binding protein, localizes to mitochondria and regulates mitochondrial biogenesis and motility in budding yeast. J Cell Biol. 2007 Jan 15;176(2):197-207.

41. Gerber AP, Herschlag D, Brown PO. Extensive association of functionally and cytotopically related mRNAs with Puf family RNA-binding proteins in yeast. PLoS Biol. 2004 Mar;2(3):E79.

42. Burston HE, Maldonado-Baez L, Davey M, Montpetit B, Schluter C, Wendland B, et al. Regulators of yeast endocytosis identified by systematic quantitative analysis. J Cell Biol. 2009 Jun 15;185(6):1097-110.

43. Machin NA, Lee JM, Barnes G. Microtubule stability in budding yeast: characterization and dosage suppression of a benomyl-dependent tubulin mutant. Mol Biol Cell. 1995 Sep;6(9):1241-59.

44. Sopko R, Huang D, Preston N, Chua G, Papp B, Kafadar K, et al. Mapping pathways and phenotypes by systematic gene overexpression. Mol Cell. 2006 Feb 3;21(3):319-30.
