# Supplementary material for: Evolutionary Conservation and Diversification of Puf RNA Binding Proteins and Their mRNA Targets
Source: PLoS Biol. 2015 Nov 20;13(11):e1002307. doi: 10.1371/journal.pbio.1002307 (PMC4654594; doi:10.1371/journal.pbio.1002307)
Supplement: S11 Text — (DOCX) [file pbio.1002307.s058.docx]

**S11 Text. Puf3 and Puf4 target evolution in Orbiliomycetes consistent with independent selective advantages.**

Saccharomycotina Puf3, Orbiliomycetes Puf3 and Puf4, and Leotiomyceta Puf4 interact with more than 150 RNAs encoding mitochondrial proteins (S15 Fig.), implying an extensive history of sequence changes in these RNAs since these species shared an ancestor. This history also raises many intriguing questions including the following: Was selective pressure involved in these changes? If so, what changes in the environment or other aspects of gene regulation led to changes in Puf protein targets? What function does (or did) each Puf play in the regulation of their target RNAs?

Models explaining how Puf3 and Puf4 targets evolved can be classified into two types, depending on whether an evolutionary pressure was involved. In one class of models an interaction with Puf4 confers a redundant fitness advantage relative to an interaction with Puf3, and interactions diversified through neutral drift. In the alternative models Puf3 and Puf4 provide fitness advantages that are not redundant, and diversification resulted from adaptive changes.

*Differences between* S. cerevisiae *Puf3 and* N. crassa *Puf4 regulatory networks suggest these proteins are not functionally redundant*

Regulatory programs can diversify through mutation and neutral drift when the change maintains fitness. In the simplest scenario of neutral drift, Puf3 and Puf4 are predicted to be functionally redundant and would regulate or affect their target RNAs in the same way. However, the results from gene expression profiling suggest the nature of regulation by Puf3 and Puf4 is different as the removal of Puf3 from *S. cerevisiae* cells led to an increase in the abundance of Puf3 targets whereas the removal of Puf4 in *N. crassa* cells led to a decrease in target abundance (compare Fig. 7 to S18 FigC). These observations suggest Puf3 and Puf4 are not functionally redundant with respect to the regulation of ancestral Puf3 targets, and this difference in a measurable consequence of Puf-RNA interactions argues against the simplest form of neutral drift model.

*Neutral drift alone is unlikely to account for the distinctions in Leotiomyceta Puf3 and Puf4 target sets*

By definition the neutral drift model states that over time the targets of Puf3 and Puf4 will be stochastically exchanged without a fitness cost. Given sufficient time for exchange, the targets for Puf3 or Puf4 would eventually compose a random subset of the targets for which their interaction with Puf3 or Puf4 confers a fitness advantage. The neutral drift model could account for an apparent exchange of targets if the rate of exchange was biased in direction (*e.g.* if mutations favored Puf4 binding site gains).

The neutral drift model predicts that Puf3 and Puf4 would be linked to the same functionally distinct subsets of RNAs and the relative frequency of Puf3 or Puf4 binding sites would be the same across in each subset. However, the conserved Leotiomyceta Puf3 targets include the majority of RNAs encoding ETC complex I proteins, whereas the majority of ancestral Puf3 targets (*e.g.* those encoding proteins involved in mitochondrial organization) became conserved targets of Leotiomyceta Puf4. Putative binding sites for Puf3, but not Puf4, are commonly found within the RNAs that encode ETC complex I proteins, and this pattern of binding sites is significantly different than the distribution within the ancestral Puf3 target RNAs (p = 10^-25^ using Fisher’s exact test with Leotiomyceta conserved target definitions and ancestral Puf3 targets refers to the intersection of conserved Saccharomycotina Puf3 targets and Leotiomyceta Puf3 or Puf4 targets). If drift were biased towards Puf4 taking over Puf3’s targets then the regulation of ETC complex I RNAs would have to be a separate function of Puf3 (*i.e.*, a function not common to Puf4) to account for its conserved interaction with Puf3 and not Puf4. Furthermore, Pezizomycotina Puf4 tends to interact with RNAs encoding the histone proteins (S24 Fig.) whereas Puf3 does not (no histones are found among conserved Puf3 targets). Thus, Puf3 and Puf4 have each maintained functionally distinct sets of RNA targets, in addition to exchanging many of ancestral Puf3 targets to Puf4 within Leotiomyceta so that the neutral drift model alone cannot account for the observed patterns in all of the Leotiomyceta Puf3 and Puf4 target sets.

*Orbiliomycetes Puf3 and Puf4 binding sequences in the ancestral Puf3 targets evolved independent of the presence of the other binding sequence*

Puf3 and Puf4 are inferred to regulate many of the ancestral Puf3 targets in Orbiliomycetes (S23 Fig.). In the neutral drift model, a Puf3 interaction with an ancestral Puf3 target would be redundant with an interaction with Puf4; in other words, it would not be advantageous to add a Puf4 site in an ancestral Puf3 target when a Puf3 binding site is already present. This predicts that Puf3 and Puf4 binding sites would be more likely to be found in different RNAs within the ancestral Puf3 target set (*i.e*., there would be a dependence in which RNAs have Puf3 and/or Puf4 sites such that they tend not be found in the same RNAs). To test this prediction, we examined the relationship of Puf3 and Puf4 motif matches within ancestral Puf3 targets in the three Orbiliomycetes. There was no evidence that Puf3 and Puf4 binding sites preferentially occurred in different ancestral Puf3 targets (S16 FigA). Furthermore, we used Bayesian inference to estimate the rates of gain and loss for Puf3 and Puf4 sites in the Orbiliomycetes lineage, and model comparison supports that the rates are not different when one or the other Puf site is already present (S16 Fig B-C, see legend for more information). The data are consistent with independence between Puf3 and Puf4 binding sites, implying that Puf3 and Puf4 have separate functions that have been selected for over evolution.
