# Supplementary material for: Evolutionary Conservation and Diversification of Puf RNA Binding Proteins and Their mRNA Targets
Source: PLoS Biol. 2015 Nov 20;13(11):e1002307. doi: 10.1371/journal.pbio.1002307 (PMC4654594; doi:10.1371/journal.pbio.1002307)
Supplement: S12 Text — (DOCX) [file pbio.1002307.s059.docx]

**S12 Text. Finer scale changes in binding site sequences of conserved Puf targets.**

Binding site sequences are not found in every transcript of our defined conserved target sets. Each of these cases could be a true negative if the binding site sequence was in fact lost (or only gained in the ancestor(s) of other species). Alternatively, a case could be a false negative if our definition of binding site sequences is too restrictive and did not fully capture the Puf’s binding interactions.

In general, binding sequence motifs do not fully discriminate targets from non-targets in experimental association data, making it a challenge to infer from bioinformatics alone when an interaction between a Puf protein and a given RNA is present within a single species and when that interaction was gained (and perhaps subsequently lost as well).

Because the association of RNAs with *S. cerevisiae* Pufs has been experimentally measured, we can use these data to test if all of the conserved targets (*i.e.*, those that are enriched with a Puf binding site across Saccharomycotina species) associate with the respective Puf in *S. cerevisiae*. Based on the association data and whether or not the RNA has a Puf motif match, the test could reveal RNAs that are not targets in *S. cerevisiae* or cases where the motif search in 3' UTRs does not account for the interaction with a Puf.

Our analysis indicates that conserved Puf targets that contain a motif match in *S. cerevisiae* are more enriched in the experimental data than those conserved targets that do not have a motif match (p = 10^-12^ for Puf3, p = 10^-7^ for Puf4, p = 0.005 for Puf5, S17 Fig.). Thus, the results suggest that a significant fraction of the conserved targets without a motif match either do not interact with the respective *S. cerevisiae* Puf or do so more transiently or with a weaker affinity. Nevertheless, as the RNAs without a motif match in the 3' UTR are still enriched relative to all other RNAs (p = 0.005 for Puf3, p = 0.01 for Puf4, p = 10^-5^ for Puf5, S17 Fig.), a component in the accounting of Puf-RNA interaction is missing. Thus, Puf proteins likely interact with additional RNA sequences not captured in the motif, and Puf proteins may also interact with the part of the mRNA outside of the 3’ UTR.

Our identification of conserved targets picks out RNAs with an enrichment of putative binding sites across a set of species but does not explicitly identify if and when targets are gained or lost. Our results above suggest that a fraction of the conserved target set have either gained binding site sequences only in part of the Saccharomycotina lineage (*i.e.,* ancestor of *S. cerevisiae* did not have binding site sequence) or were gained then lost in an ancestors of *S. cerevisiae*. Future computational work analyzing more species coupled with experimental data from species other than *S. cerevisiae* will reveal the finer scale dynamics of binding site evolution. It is of fundamental importance to determine if the set of interacting RNAs is the same and is complete for a given functional theme across organisms. If the target set varies from species to species, it would represent drift or selection for subsets of genes and more granular regulation within the sets of genes that we can currently define thematically.
