# Supplementary material for: Evolutionary Conservation and Diversification of Puf RNA Binding Proteins and Their mRNA Targets
Source: PLoS Biol. 2015 Nov 20;13(11):e1002307. doi: 10.1371/journal.pbio.1002307 (PMC4654594; doi:10.1371/journal.pbio.1002307)
Supplement: S13 Text — (DOCX) [file pbio.1002307.s060.docx]

**S13 Text. Changes at the post-transcriptional level compared to changes in transcriptional regulation.**

Transcription factors and RNA binding proteins tend to regulate groups of genes or RNAs that encode related proteins. Several sets of Puf RNA targets are apparently regulated at the transcriptional level through the binding of a common transcription factor. The evolution of transcription factor binding to two of these sets has been studied, allowing us to compare the relationship between evolutionary changes at these different levels of gene expression.

*Non-concurrent changes in transcriptional and post-transcriptional regulatory programs*

We compared changes in Puf evolution to changes in transcription linked to the emergence of respiro-fermentative yeasts (same time as whole genome duplication in Fig. 8, #1 in S25 Fig.), which grow in the absence of oxygen and prefer to ferment glucose even when oxygen is present. Prior to this emergence and in species that cannot ferment aerobically, the expression of transcripts encoding components of the cytoplasmic and mitochondrial ribosome are correlated [1-2]. In respiro-fermentative yeast, expression of mitochondrial ribosome components and the cytoplasmic ribosome components is not correlated [1-2], and the loss of this correlation (relative to the ancestral lineage) is associated with the loss of a common element in the promoters of mitochondrial ribosomal genes that remains in the cytoplasmic ribosomal and ribosome biogenesis genes [1,3]. This single element has been alternatively called the rapid growth element (RGE) [1] or the ribosomal RNA processing element (RRPE; we use this term herein) [4]. The transcription factors that act through this element have been suggested to include Stb3 [5-6] and Sfp1 [6-8]. Almost every RNA encoding a component of the mitochondrial ribosome is conserved as a Puf3 target in Saccharomycotina species (S23 Fig., #4 in S25 Fig.), which include both respirative (*e.g.*, *C. albicans*) and respiro-fermentative yeast (*e.g.* *S. cerevisiae*). This commonality implies that the origin of Puf3 regulation of mitochondrial ribosomal transcripts occurred prior to and distinct from the loss, in a distinct sublineage of the Saccharomycotina, of their regulation at the transcriptional level through the RRPE.

In another example, *S. cerevisiae* Puf4 tends to bind many RNAs involved in ribosome biogenesis, and the evolutionary origin of its interaction with these RNAs appears to have coincided with the divergence of Saccharomycotina. These changes appeared after the origin of presumed transcriptional regulation through the RRPE in the promoters of ribosome biogenesis genes, whereas this transcriptional regulation has been suggested to pre-date the origin of fungi [9] (#7 in S25 Fig.). Thus, changes in gene expression for ribosomal biogenesis involving RNA transcripts in the cytoplasm (where Puf4 is found [10]) occurred after these RNAs began to be regulated in a coordinated fashion at the level of transcription.

*Possible contemporaneous changes in the transcriptional and post-transcriptional regulation of ribosome biogenesis genes*

The transcription factors Pbf1/Tod6 and Pbf2/Dot6 are paralogous transcription factors that regulate genes involved in ribosome biogenesis [4,6,11], a theme observed among RNA targets of *S. cerevisiae* Puf4. Pbf1 and Pbf2 bind to the polymerase A and C (PAC) element, and this element is enriched within promoters of ribosome biogenesis genes in CTG clade and Saccharomycetaceae species but not in *Y. lipolytica* [9,12]. Within the resolution of our phylogenetic tree, the inferred gain of Pbf1/2 regulation (#2 in S25 Fig.) occurred at the time Puf4 duplicated and when Puf4 started binding RNAs encoding ribosome biogenesis proteins (#3 in S25 Fig.). These putative contemporaneous changes in ribosome biogenesis gene expression may reflect an advantage for both transcriptional and post-transcriptional regulation or the need for coordination between the two steps in expression.

**References**

1. Ihmels J, Bergmann S, Gerami-Nejad M, Yanai I, McClellan M, Berman J, et al. Rewiring of the yeast transcriptional network through the evolution of motif usage. Science. 2005 Aug 5;309(5736):938-40.

2. Tsankov AM, Thompson DA, Socha A, Regev A, Rando OJ. The role of nucleosome positioning in the evolution of gene regulation. PLoS Biol. 2010;8(7):e1000414.

3. Hogues H, Lavoie H, Sellam A, Mangos M, Roemer T, Purisima E, et al. Transcription factor substitution during the evolution of fungal ribosome regulation. Mol Cell. 2008 Mar 14;29(5):552-62.

4. Hughes JD, Estep PW, Tavazoie S, Church GM. Computational identification of cis-regulatory elements associated with groups of functionally related genes in Saccharomyces cerevisiae. J Mol Biol. 2000 Mar 10;296(5):1205-14.

5. Liko D, Slattery MG, Heideman W. Stb3 binds to ribosomal RNA processing element motifs that control transcriptional responses to growth in Saccharomyces cerevisiae. J Biol Chem. 2007 Sep 7;282(36):26623-8.

6. Zhu C, Byers KJ, McCord RP, Shi Z, Berger MF, Newburger DE, et al. High-resolution DNA-binding specificity analysis of yeast transcription factors. Genome Res. 2009 Apr;19(4):556-66.

7. Harbison CT, Gordon DB, Lee TI, Rinaldi NJ, Macisaac KD, Danford TW, et al. Transcriptional regulatory code of a eukaryotic genome. Nature. 2004 Sep 2;431(7004):99-104.

8. Marion RM, Regev A, Segal E, Barash Y, Koller D, Friedman N, et al. Sfp1 is a stress- and nutrient-sensitive regulator of ribosomal protein gene expression. Proc Natl Acad Sci U S A. 2004 Oct 5;101(40):14315-22.

9. Brown SJ, Cole MD, Erives AJ. Evolution of the holozoan ribosome biogenesis regulon. BMC Genomics. 2008;9:442.

10. Gerber AP, Herschlag D, Brown PO. Extensive association of functionally and cytotopically related mRNAs with Puf family RNA-binding proteins in yeast. PLoS Biol. 2004 Mar;2(3):E79.

11. Badis G, Chan ET, van Bakel H, Pena-Castillo L, Tillo D, Tsui K, et al. A library of yeast transcription factor motifs reveals a widespread function for Rsc3 in targeting nucleosome exclusion at promoters. Mol Cell. 2008 Dec 26;32(6):878-87.

12. Lavoie H, Hogues H, Mallick J, Sellam A, Nantel A, Whiteway M. Evolutionary tinkering with conserved components of a transcriptional regulatory network. PLoS Biol. 2010 Mar;8(3):e1000329.
