# Supplementary material for: Evolutionary Conservation and Diversification of Puf RNA Binding Proteins and Their mRNA Targets
Source: PLoS Biol. 2015 Nov 20;13(11):e1002307. doi: 10.1371/journal.pbio.1002307 (PMC4654594; doi:10.1371/journal.pbio.1002307)
Supplement: S14 Text — (DOCX) [file pbio.1002307.s061.docx]

**S14 Text. Models for the history of Puf3 and Puf4 interacting with a common set of more than 150 mRNAs.**

Multiple lines of evidence from genomic sequence analysis and experimental data indicate that Pezizomycotina Puf4 binds RNAs encoding mitochondrial proteins, particularly the ones involved in mitochondrial organization and related to the targets of Puf3 in Saccharomycotina. By parsimony, Puf4 gained these RNAs as targets in the ancestor of all Pezizomycotina species used here (Fig. 8).

*Model #1 for Puf3 history: Binding sites gained in ancestor of Saccharomycotina and Pezizomycotina, then lost in an ancestor to Leotiomyceta*

Two parsimonious models account for the sharing of Puf3 targets in Saccharomycotina and Orbiliomycetes, the earliest branching group in the Pezizomycotina lineage, given the phylogenetic relationships of the fungal species derived herein and in related work [83-84,86]**.** In one model, Puf3 binding sites were gained in an ancestor of Saccharomycotina and Pezizomycotina fungi, and subsequently conserved in Saccharomycotina and in the Orbiliomycetes but lost in an ancestor to the Leotiomyceta species (Fig. 8, S21 FigA.). This model implies that both Puf3 and Puf4 in the Pezizomycotina ancestor bound to RNAs encoding mitochondrial proteins, and that this binding was subsequently conserved within species of the basal lineage of Orbiliomycetes (see phylogeny in Fig. 6, Fig. 8). After the divergence of Orbiliomycetes from the rest of Pezizomycotina, Puf3 sites were lost in most RNAs encoding mitochondrial proteins while Puf4 sites remain (Fig. 8).

*Model #2 for Puf3 history: Binding sites gained independently in Saccharomycotina and Orbiliomycetes*

In the second model, Puf3 binding sites were gained through one series of events in the Saccharomycotina lineage and an independent series of events in the Orbiliomycetes lineage, resulting in parallel or convergent evolution (S21 FigA.). This model implies that Puf3 binding sites in Orbiliomycetes were gained after the Puf4 binding sites were already present. In this model the gain of regulation by Puf3 in an ancestor of Saccharomycotina and by Puf4 in an ancestor of Pezizomycotina came after the lineages diverged.

*Model #1 emerges as more likely in the full context of Puf-RNA evolution*

The two proposed models for Puf3 are equally parsimonious as both entail two collective Puf3 target set changes: the first model involves one series of gains and one subsequent series of losses, and the second model involves two series of binding site gains.

We can consider the broad mitochondrial context of Puf-RNA evolution to determine the more likely model. Puf3 is implicated in mitochondrial function in Saccharomycotina, Orbiliomycetes, and Leotiomyceta through interaction with RNAs involved in mitochondrial translation and organization or through the RNAs encoding ETC complex I components. The simplest model, which is consistent with model #1 above, is that Puf3 has maintained a connection to the mitochondrion that originated in an ancestor of Saccharomycotina and Pezizomycotina. In contrast, model #2 implies Puf3 gained a mitochondrial function separately in Saccharomycotina and in Pezizomycotina. Thus, model #1 emerges as more parsimonious when considering the totality of Puf3’s connection to RNAs involved in mitochondrial function.

In our proposed, combined model of Puf3 and Puf4 evolution, the shared features of their interaction with RNA could have enabled the transfer of targets from Puf3 to Puf4, especially as the binding sequences for these proteins are highly similar and thus require fewer evolutionary changes than most specificity switches. Indeed, *in vitro* binding data from *S. cerevisiae* Puf3 and Puf4 show that these Pufs can have nanomolar dissociation constants for each other's preferred binding sites, although the interaction with their preferred target sites is one to two orders of magnitude lower [49,54]. Thus, the transfer of targets from Puf3 to Puf4 may have been facilitated by a promiscuous ability of Puf4 to recognize existing Puf3 sites as well as by the ease of transitioning a Puf3 site into a Puf4 site.
