# Supplementary material for: Evolutionary Conservation and Diversification of Puf RNA Binding Proteins and Their mRNA Targets
Source: PLoS Biol. 2015 Nov 20;13(11):e1002307. doi: 10.1371/journal.pbio.1002307 (PMC4654594; doi:10.1371/journal.pbio.1002307)
Supplement: S15 Text — (DOCX) [file pbio.1002307.s062.docx]

**S15 Text. Conservation of RNA-interacting residues after Puf4 duplication suggest possible changes in shape of Puf domain as the origin of binding specificity divergence.**

Our characterization of the RNA recognition of Pezizomycotina Puf4 revealed binding to multiple motifs (Fig. 5B). After Puf4 duplicated in Saccharomycotina (to give Puf4 and Puf5), the binding specificity of the paralogs became restricted with respect to the ancestral specificity and diverged with respect to each other (Fig. 5B, Fig. 8 #3). Despite the different binding specificities, the RNA-contacting residues of Saccharomycotina Puf4 and Puf5 are identical and have been conserved. Thus, changes outside of the interacting residues determine the differences in binding specificity. Prior work has shown that the curvature of the crescent-shaped RNA binding domain is important for the recognition of different length binding sites of *S. cerevisiae* Puf3 and Puf4 [1], and an analogous change in curvature could explain the binding specificity differences between Puf4 and Puf5. It is possible that Pezizomycotina and ancestral Puf4 proteins exhibited broader specificity by having a more dynamic Puf curvature that could adopt conformations similar to modern-day Saccharomycotina Puf4 and Puf5 and thereby bind to RNA motifs recognized by Saccharomycotina Puf4 and Puf5 (Fig. 5B).

**References**

1. Miller MT, Higgin JJ, Hall TM. Basis of altered RNA-binding specificity by PUF proteins revealed by crystal structures of yeast Puf4p. Nat Struct Mol Biol. 2008 Apr;15(4):397-402.
