# Supplementary material for: Evolutionary Conservation and Diversification of Puf RNA Binding Proteins and Their mRNA Targets
Source: PLoS Biol. 2015 Nov 20;13(11):e1002307. doi: 10.1371/journal.pbio.1002307 (PMC4654594; doi:10.1371/journal.pbio.1002307)
Supplement: S16 Text — (DOCX) [file pbio.1002307.s063.docx]

**S16 Text. Inferring the History of Acquisition, Loss, and Changes in the Regulatory Specificity of Puf Proteins in Fungal Evolution.**

Fig. 8 summarizes events in the evolution of Puf proteins and their targets in fungi, as derived from our analyses and experiments. One of the most straightforward analyses is determining which Puf genes are present in a genome and thus when in the evolutionary history of fungi each Puf gene was acquired or lost. Our phylogenetic comparisons provide strong evidence for six such events in the ~600 million years of evolution spanning Taphrinomycotina, Pezizomycotina, and Saccharomycotina (Fig. 8, circles). The Taphrinomycotina experienced a duplication of Puf1, Puf4, and Puf8; after the divergence of the Saccharomycotina lineage, Puf8 was deleted; after Puf8 was deleted, Puf4 was duplicated to give Puf4 and Puf5; and Puf1 was duplicated as part of a whole genome duplication [1-2] about 100-150 million years ago to give Puf1 and Puf2.

Puf1 and Puf2 bind highly overlapping target sets in *S. cerevisiae* [3]. Although this is consistent with their being the most recently duplicated Puf genes, the persistence of this extensive overlap in targets for 100-150 million years raises the question of what independent selective advantage might be conferred by the respective proteins. One possibility is that they might mediate two distinct regulatory programs acting on the same set of genes.

There have also been changes in sequence specificity throughout Puf evolution in fungi (Fig. 8, triangles). Puf4 and Puf5, as noted above, have diverged in their sequence specificity (Fig. 6B), and bind RNA targets that fall into distinct functional classes (S24 FigA) [3]. Pezizomycotina Puf4 appears to have broader sequence specificity than Puf4 or Puf5 from Saccharomycotina; indeed it appears to recognize RNA sequences that encompass RNA recognition properties of both Saccharomycotina Puf4 and Puf5 (Fig. 5B). Presumably functional specialization of Puf4 and Puf5 following their duplication required corresponding specialization of their recognition motifs. There are also several RNA targets shared between Puf4 and Puf5, including RNAs coding for histones, suggesting that these joint targets form a conserved set distinct from those recognized by Puf4 or Puf5 alone. Although Puf4 and Puf5 binding sequences can overlap, most of the common targets are predicted to contain separate binding sites for Puf4 and Puf5 (Dataset S10). Whether Puf4 and Puf5 provide redundant or differential (*e.g.*, combinatorial or competitive) regulation of the histone transcripts is an interesting question for further research.

Previous functional work has shown that a cytosine two residues upstream of the conserved UGUA motif enhances *S. cerevisiae* Puf3 affinity but not Puf4 affinity and thus can be used to help in binding discrimination [4]. Conservation of this ancillary interaction arose within the Saccharomycotina lineage (S7 Fig.), and thus does not appear to play a role in discriminating between Puf3 and Puf4 targets in Pezizomycotina (Fig. 8, triangle at #3). Perhaps the Puf4/Puf5 duplication, which is coincident within the resolution of our tree, provided a driving force for this change.

The newly identified Puf8 gene is present in most of the fungi represented in this study, the exception being the majority of Saccharomycotina species (S2 Table) leading to a model that it was acquired by an ancestral fungal genome near the origin of fungal lineage and lost in Saccharomycotina. We do not know its recognition motif; the only functional data for this protein of which we aware of is for its two orthologs in *S. pombe* (Mpf1/SPAC4G9.05 and Mcp2/ SPCC1682.08c). The *S. pombe* Puf8 orthologs are both expressed late in meiosis [5], but we do not know if this meiosis-specific expression is conserved in other fungi.

**References**

1. Kellis M, Birren BW, Lander ES. Proof and evolutionary analysis of ancient genome duplication in the yeast Saccharomyces cerevisiae. Nature. 2004 Apr 8;428(6983):617-24.

2. Byrne KP, Wolfe KH. The Yeast Gene Order Browser: combining curated homology and syntenic context reveals gene fate in polyploid species. Genome Res. 2005 Oct;15(10):1456-61.

3. Gerber AP, Herschlag D, Brown PO. Extensive association of functionally and cytotopically related mRNAs with Puf family RNA-binding proteins in yeast. PLoS Biol. 2004 Mar;2(3):E79.

4. Zhu D, Stumpf CR, Krahn JM, Wickens M, Hall TM. A 5' cytosine binding pocket in Puf3p specifies regulation of mitochondrial mRNAs. Proc Natl Acad Sci U S A. 2009 Dec 1;106(48):20192-7.

5. Kasama T, Shigehisa A, Hirata A, Saito TT, Tougan T, Okuzaki D, et al. Spo5/Mug12, a putative meiosis-specific RNA-binding protein, is essential for meiotic progression and forms Mei2 dot-like nuclear foci. Eukaryot Cell. 2006 Aug;5(8):1301-13.
