# Supplementary material for: Evolutionary Conservation and Diversification of Puf RNA Binding Proteins and Their mRNA Targets
Source: PLoS Biol. 2015 Nov 20;13(11):e1002307. doi: 10.1371/journal.pbio.1002307 (PMC4654594; doi:10.1371/journal.pbio.1002307)
Supplement: S17 Text — (DOCX) [file pbio.1002307.s064.docx]

**S17 Text. Dating the Puf4 and Puf5 duplication.**

Based on sequence similarity of the *S. cerevisiae* proteins and the matching and conserved RNA-contacting residues, Puf4 and Puf5 are paralogs (S10 Fig., S22 Fig.). According to our ortholog classification, Puf4 proteins are found throughout the fungal lineage but Puf5 proteins are only found in a subset of Saccharomycotina species (S2 Table, S10 Fig.). From the distribution of Puf5 proteins in Saccharomycotina species, we infer that Puf5 arose from the duplication in the ancestor of Saccharomycotina species that contain a copy of both Puf4 and Puf5. Within the resolution of our phylogenetic tree, the Puf4/5 duplication occurred after *Y. lipolytica* diverged from the rest of the studied species.
