# Supplementary material for: Evolutionary Conservation and Diversification of Puf RNA Binding Proteins and Their mRNA Targets
Source: PLoS Biol. 2015 Nov 20;13(11):e1002307. doi: 10.1371/journal.pbio.1002307 (PMC4654594; doi:10.1371/journal.pbio.1002307)
Supplement: S11 Fig — At the top of each panel is a sequence similarity network made from 10mer sequences found to be enriched in the target set compared to the nontarget set (Materials and Methods). The sequence similarity network was used to identify subsets of 10mers that could be grouped together based on a common feature. A node represents a 10mer, and an edge (gray line) is present between nodes that differ by only one nucleotide. 10mers with two or more nucleotides different from all other 10mers are not shown. The remainder of the panel contains a key and summary for groups made from the sequence similarity network. Each panel is formatted the same way. For "# of UTRs matching 10mers", each UTR can only be assigned one 10mer, and the assigned 10mer is the first match found in the iterative search. (A) Network for Puf3 in post-whole-genome-duplication (post-WGD) species. In-group consists of 1,534 S. cerevisiae Puf3 targets and orthologs in post-WGD species (n = 8). The top 48 10mers are represented one or more times in 71.1% of UTRs (1090/1534), compared to 9.7% of other UTRs. (B) Network for Puf4 in post-WGD species. In-group consists of 1,399 S. cerevisiae Puf4 targets and orthologs in post-WGD species (n = 8). The top 66 10mers are represented one or more times in 53.5% of UTRs (748/1399), compared to 7.2% of other UTRs. (C) Network for Puf5 in post-WGD species. In-group consists of 1343 S. cerevisiae Puf5 targets and orthologs in post-WGD species (n = 8). The top 46 10mers are represented one or more times in 48.7% of UTRs (654/1343), compared to 5.6% of other UTRs. (D) Network for Pezizomycotina Puf4. In-group consists of 8,418 orthologs of conserved Saccharomycotina Puf3 targets in Leotiomyceta species (n = 42). The top 100 10mers are represented one or more times in 57% of UTRs (4817/8418), compared to 11.2% of other UTRs. (PDF) [file pbio.1002307.s021.pdf]

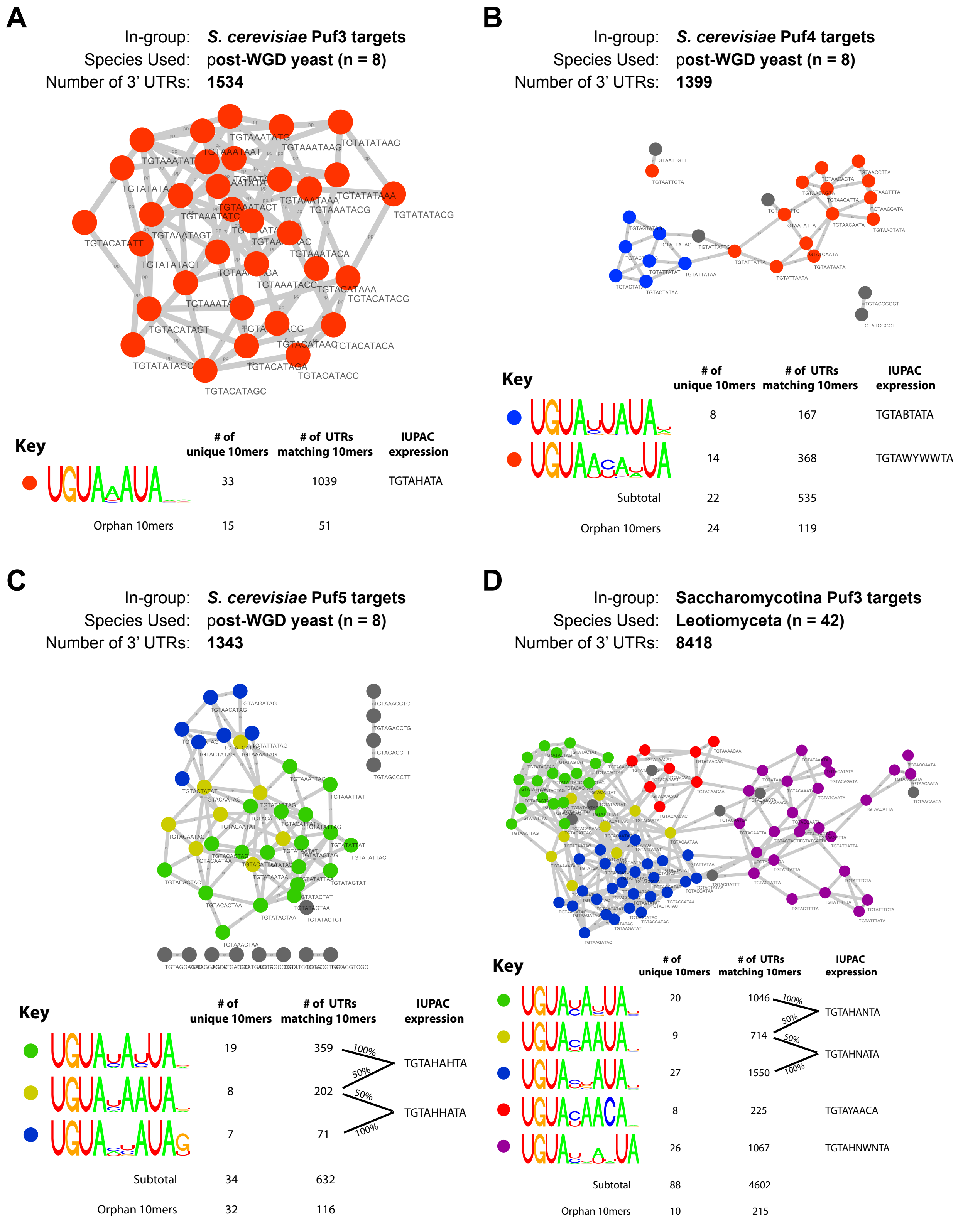

A
In-group: S. cerevisiae Puf3 targets
Species Used: post-WGD yeast (n = 8)
Number of 3' UTRs: 1534
Key
# of unique 10mers
# of UTRs matching 10mers
IUPAC expression
33 1039 TGTAHATA
Orphan 10mers 15 51
B
In-group: S. cerevisiae Puf4 targets
Species Used: post-WGD yeast (n = 8)
Number of 3' UTRs: 1399
Key
# of unique 10mers
# of UTRs matching 10mers
IUPAC expression
8 167 TGTABTATA
14 368 TGTAWYWWTA
Subtotal 22 535
Orphan 10mers 24 119
C
In-group: S. cerevisiae Puf5 targets
Species Used: post-WGD yeast (n = 8)
Number of 3' UTRs: 1343
Key
# of unique 10mers
# of UTRs matching 10mers
IUPAC expression
19 359
8 202
7 71
100% 50% TGTAHAHTA
50% 100% TGTAHHATA
Subtotal 34 632
Orphan 10mers 32 116
D
In-group: Saccharomycotina Puf3 targets
Species Used: Leotiomyceta (n = 42)
Number of 3' UTRs: 8418
Key
# of unique 10mers
# of UTRs matching 10mers
IUPAC expression
20 1046
9 714
27 1550
100% 50% TGTAHANTA
50% 100% TGTAHNATA
8 225 TGTAYAACA
26 1067 TGTAHNWNTA
Subtotal 88 4602
Orphan 10mers 10 215

Figure S11
